# Impact of the Anthryl Linking Mode on the Photophysics and Excited-State Dynamics of Re(I) Complexes [ReCl(CO)_3_(4′-An-terpy-κ^2^N)]

**DOI:** 10.1021/acs.inorgchem.2c02160

**Published:** 2022-09-14

**Authors:** Magdalena Małecka, Agata Szlapa-Kula, Anna M. Maroń, Przemyslaw Ledwon, Mariola Siwy, Ewa Schab-Balcerzak, Karolina Sulowska, Sebastian Maćkowski, Karol Erfurt, Barbara Machura

**Affiliations:** † Institute of Chemistry, University of Silesia, 9th Szkolna Street, 40-006 Katowice, Poland; ‡ Department of Physical Chemistry and Technology of Polymers, Silesian University of Technology, Strzody 9, 44-100 Gliwice, Poland; § Centre of Polymer and Carbon Materials, Polish Academy of Sciences, 34 M. Curie-Sklodowska Street, 41-819 Zabrze, Poland; ∥ Nanophotonics Group, Institute of Physics, Faculty of Physics, Astronomy and Informatics, Nicolaus Copernicus University, 5 Grudziadzka Street, 87-100 Torun, Poland; ⊥ Department of Chemical Organic Technology and Petrochemistry, Silesian University of Technology, Krzywoustego 4, 44-100 Gliwice, Poland

## Abstract

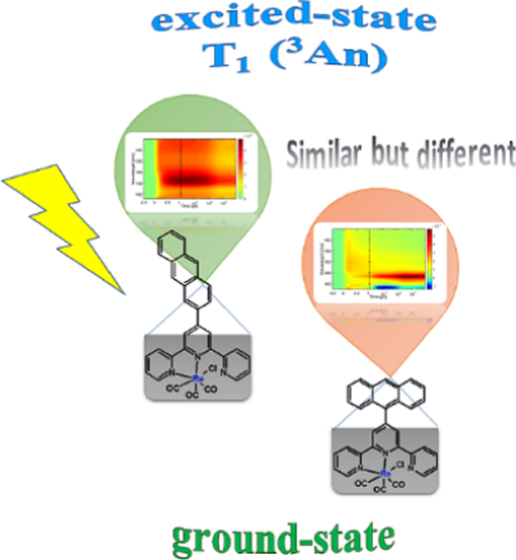

Rhenium(I) complexes with 2,2′:6′,2″-terpyridines (terpy) substituted with 9-anthryl (**1**) and 2-anthryl (**2**) were synthesized, and the impact of the anthryl linking mode on the ground- and excited-state properties of resulting complexes [ReCl(CO)_3_(4′-An-terpy-κ^2^N)] (An—anthryl) was investigated using a combination of steady-state and time-resolved optical techniques accompanied by theoretical calculations. Different attachment positions of anthracene modify the overlap between the molecular orbitals and thus the electronic coupling of the anthracene and {ReCl(CO)_3_(terpy-κ^2^N)} chromophores. Following the femtosecond transient absorption, the lowest triplet excited state of both complexes was found to be localized on the anthracene chromophore. The striking difference between **1** and **2** concerns the triplet-state formation dynamics. A more planar geometry of 2-anthryl-terpy (**2**), and thus better electronic communication between the anthracene and {ReCl(CO)_3_(terpy-κ^2^N)} chromophores, facilitates the formation of the ^3^An triplet state. In steady-state photoluminescence spectra, the population ratio of ^3^MLCT and ^3^An was found to be dependent not only on the anthryl linking mode but also on solvent polarity and excitation wavelengths. In dimethyl sulfoxide (DMSO), compounds **1** and **2** excited with λ_exc_ > 410 nm show both ^3^MLCT and ^3^An emissions, which are rarely observed. Additionally, the abilities of the designed complexes for ^1^O_2_ generation and light emission under the external voltage were preliminary examined.

## Abstract 2

The impact of the anthryl linking mode on the ground- and excited-state properties of [ReCl(CO)_3_(4′-An-terpy-κ^2^N)] with 2,2′:6′,2″-terpyridines (terpy) substituted with 9-anthryl (**1**) and 2-anthryl (**2**) was thoroughly investigated. Different attachment positions of anthracene were evidenced to modify the overlap between the molecular orbitals and electronic coupling of the anthracene and {ReCl(CO)_3_(terpy-κ^2^N)} chromophores and thus the optical properties of the resulting complexes. The striking difference between **1** and **2** was demonstrated in the triplet-state formation dynamics.

## Introduction

Since the first report on Re(I) carbonyl diamine complexes by Wrighton and Morse,^1^ a large number of experimental and theoretical studies have been carried out to understand the ground- and excited-state properties of [Re(CO)_3_X(N^∩^N)]^*n*+^ (X—ancillary ligand, *n* = 0 or 1). Structural modifications of the chelating organic ligand (N^∩^N) and variations of the ancillary one (X = halogens, carboxylates, phosphines, nitriles, isonitriles, etc.) have been found to be effective tools for fine-tuning the metal-to-ligand charge-transfer (MLCT) state of these systems or switching it to ligand-to-ligand charge transfer (LLCT), halogen-to-ligand charge transfer (XLCT), intraligand charge transfer (ILCT), intraligand (IL), and σ-bond-to-ligand charge transfer (SBLCT) ones.^2−8^ Relatively simple synthetic procedure, thermal stability, and photophysical properties make Re(I) carbonyl polypyridyl complexes very promising agents for bioimaging and photodynamic therapy (PDT) of cancer,^9−18^ organic light-emitting diodes (OLED),^19−23^ and photocatalysis for selective reduction of CO_2_ to CO.^24−29^ The control of electronically excited states in [Re(CO)_3_X(N^∩^N)]^*n*+^ systems, and thus the ability to tune their photophysical properties in the context of such many applications, is generally realized by the manipulation of the diimine core, substitution of the diimine ligand with π-conjugated organic chromophores, electron-donating or -withdrawing substituents, and variations of the ancillary X ligand.^1−29^ More specifically, Re(I) carbonyl chromophores with prolonged excited-state lifetimes have been successfully obtained using two popular concepts: (i) via the attachment of π-conjugated organic chromophores to the diimine ligand and formation of bichromophoric systems featuring the triplet-state equilibrium between ^3^MLCT and ^3^IL,^30−32^ and (ii) via introduction of electron-rich groups into the diimine unit and switching the emitting state from ^3^MLCT to ^3^ILCT.^33,34^ Excellent examples of such systems are Re(I) complexes with *N*-(1,10-phenanthroline)-4-(1-piperidinyl)naphthalene-1,8-dicarboximide^30^ and 1,10-phenanthroline substituted with triphenylamine at the 5-position,^33^ with approximately 3000-fold and 30-fold longer excited-state lifetimes compared to the parent complex [ReCl(CO)_3_(phen)], respectively. Dramatic enhancement of the excited-state lifetime has also been reported among terpyridine Re(I) systems. As a result of the decoration of C_6_H_5_-terpy with the dimethylamine group and formation of the ^3^ILCT excited state in [ReCl(CO)_3_(Me_2_N-C_6_H_4_-terpy-κ^2^N)], the lifetime of the resulting complex became ca. 260 times longer than that for [ReCl(CO)_3_(C_6_H_5_-terpy-κ^2^N)] with the lowest triplet state of the ^3^MLCT character.^34^

In our recent work,^35^ we investigated the impact of selected π-conjugated aryl chromophores on the photophysical behavior of [ReCl(CO)_3_(4′-Ar*^n^*-terpy-κ^2^N)]. We demonstrated that Re(I) complexes with 2,2′:6′,2″-terpyridines (terpy) substituted with 1-naphthyl-, 2-naphthyl-, and 9-phenanthryl are typical ^3^MLCT emitters, and naphthyl- and phenanthryl substituents have a negligible effect on the energies of the ^1^MLCT absorption and ^3^MLCT emission bands. On the contrary, the attachment of the electron-rich pyrenyl group leads to a bathochromic shift of the visible absorption accompanied by a significant increase in its intensity relative to the parent complex [ReCl(CO)_3_(terpy-κ^2^N)],^36,37^ as well as substantial enhancement of the room-temperature (RT) photoluminescence lifetime of [ReCl(CO)_3_(4′-(1-pyrenyl)-terpy-κ^2^N)] (4.4 μs) in comparison to [ReCl(CO)_3_(terpy-κ^2^N)] (3 ns) due to the establishment of the triplet-state equilibrium between the pyrenyl and Re(I) coordination framework. Further, we demonstrated that the excitation of [ReCl(CO)_3_(4′-pyrenyl-terpy-κ^2^N)] populates predominately the ^1^ILCT state, which undergoes the energy transfer to the ^1^MLCT* state via the Förster resonance energy transfer (FRET) mechanism. The ^1^MLCT state formed in this process is transformed into ^3^MLCT* by femtosecond intersystem crossing (ISC). In the next step, triplet–triplet energy transfer occurs from the relaxed ^3^MLCT state to a lower energy ^3^IL/^3^ILCT state localized on the pyrenyl-terpy ligand.

**Scheme 1:**
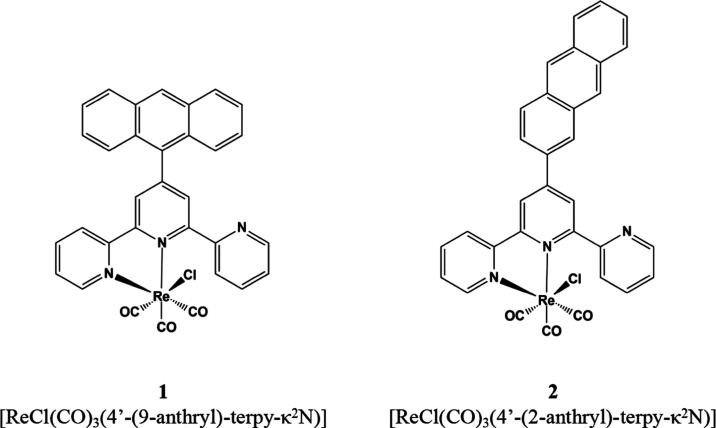
Structural Formulas of Re(I) Complexes Investigated in This Study

Continuing exploration of this field, two Re(I) complexes with 2,2′:6′,2″-terpyridines (terpy) substituted with 9-anthryl (**1**) and 2-anthryl (**2**) were synthesized to investigate the impact of the anthryl group and its linking mode on the ground- and excited-state properties of resulting complexes [ReCl(CO)_3_(4′-An-terpy-κ^2^N)] (Scheme 1). In similarity to pyrene, the anthracene chromophore (An) is well known for its valuable optical and electronic properties. Its attachment to polypyridyl ligands may lead to the energy transfer from ^3^MLCT to a long-lived ^3^IL excited state or, if ^3^MLCT and ^3^IL states have similar energies, formation of an excited-state equilibrium in metal complexes.^38−45^ Anthracene is also a rare example of a compound, which shows ISC via the upper excited state, that is T_2_, which is isoenergetic to the S_1_ state.^46,47^

Most remarkably, the only difference between designed complexes **1** and **2** is the relative orientation of anthracene and {ReCl(CO)_3_(terpy-κ^2^N)} chromophores, which was achieved by appending anthracene via its 9-position or 2-position to the terpy ligand. Therefore, by elimination of additional structural factors, this unique pair gives the opportunity to explore the impact of the mutual chromophore orientation and thus their electronic communication on light-triggered processes in such bichromophoric systems. The optimization of the photophysical properties of transition metal complexes by spatial effects still remains a great challenge, and comprehensive studies in this area are strongly desirable for the rational design of new photoluminescent materials with predefined photophysical properties.

In the present work, an insight into the ground- and excited-state properties of **1** and **2** was achieved using a combination of steady-state and time-resolved optical techniques accompanied by theoretical calculations. In addition, the abilities of the designed complexes for ^1^O_2_ generation and light emission under the external voltage were preliminary examined.

## Experimental Section

### Materials

The ligands 9-anthryl-terpy and 2-anthryl-terpy were prepared according to the literature method.^48^ Re(CO)_5_Cl, solvents for synthesis (of reagent grade) and for spectroscopic studies (of high-performance liquid chromatography (HPLC) grade), diphenylisobenzofuran (DPBF), [Ru(bipy)_3_](PF_6_)_2_, poly(9-vinylcarbazole) (PVK, *M*_n_ = 25 000–50 000; Sigma-Aldrich), poly(3,4-(ethylenedioxy)thiophene):poly-(styrenesulfonate) (PEDOT:PSS) (0.1–1.0 S·cm^–1^), and substrates with pixilated indium tin oxide (ITO) anodes (Ossila) were all commercially available and used without further purification.

### Preparation of [ReCl(CO)_3_(4′-(9-anthryl)-terpy-κ^2^N)] (**1**) and [ReCl(CO)_3_(4′-(2-anthryl)-terpy)] (**2**)

The corresponding 4′-(anthracen-9-yl)-2,2′:6′,2″-terpyridine (9-anthryl-terpy) or 4′-(anthracen-2-yl)-2,2′:6′,2″-terpyridine (2-anthryl-terpy) ligand (0.27 mmol) was added to a suspension of [Re(CO)_5_Cl] (0.27 mmol) and toluene (35 mL). The reaction mixture was heated at reflux for 8 h under an argon atmosphere. The formed precipitate was filtered off and washed with diethyl ether, dried in the air, and then purified by repeated recrystallization from toluene.

#### [ReCl(CO)_3_(4′-(9-anthryl)-terpy-κ^2^N)] (**1**)

Yield: 80%. ^1^H NMR (400 MHz, dimethyl sulfoxide (DMSO)) δ 9.14 (d, *J* = 5.2 Hz, 1H, H^C1^), 9.06 (s, 1H, H^D8^), 8.90–8.84 (m, 2H, H^B4,C4^), 8.78 (d, *J* = 4.5 Hz, 1H, H^A1^), 8.31–8.21 (m, 3H, H^C3,D3,D13^), 8.07–7.97 (m, 3H, H^B2,D6,D10^), 7.79–7.75 (m, 1H, H^C2^), 7.73 (d, *J* = 8.9 Hz, 1H, H^A4^), 7.65–7.47 (m, 6H, H^A2,A3,D4,D5,D11,D12^). ^13^C NMR (100 MHz, DMSO) δ 197.74 (C^CO^), 194.54 (C^CO^), 191.20 (C^CO^), 161.16 (C^B1^), 157.35 (C^A5^), 157.26 (C^B5^), 156.36 (C^C5^), 152.83 (C^C1^), 150.96 (C^D1^), 149.19 (C^A1^), 140.03 (C^C3^), 137.31 (C^B3^), 136.88 (C^B2^), 131.26 (C^D2^), 130.72 (C^D7^), 130.68 (C^D9^), 129.54 (C^D3^), 128.74 (C^D14^), 128.66 (C^D6^), 128.60 (C^D10^), 128.41 (C^C4^), 127.45 (C^C2^), 127.07 (C^D4^), 126.92 (C^D5^), 126.15 (C^D8^), 125.68 (C^D12^), 125.66 (C^D11^), 125.51 (C^A4^), 125.46 (C^B4^), 125.27 (C^D13^), 125.04 (C^A3^), 124.79 (C^A2^). IR (KBr, cm^–1^): 2023 (vs), 1926 (vs) and 1896 (vs) ν(C≡O); 1611 (m), ν(C=N) and ν(C=C). High-resolution mass spectrometry (HRMS) (electrospray ionization (ESI)) (*m*/*z*): [M – Cl]^+^ calcd for [C_32_H_19_N_3_O_3_Re]^+^ 680.0984. Found 680.0987. Anal. Calcd for C_32_H_19_ClN_3_O_3_Re (715.17 g·mol^–1^): C, 53.74; H, 2.68; N, 5.88. Found: C, 53.65; H, 2.74; N, 5.80.

#### [ReCl(CO)_3_(4′-(2-anthryl)-terpy)] (**2**)

Yield: 75%. ^1^H NMR (400 MHz, DMSO) δ 9.28 (s, 1H), 9.17 (d, *J* = 8.3 Hz, 1H), 9.10–9.07 (m, 2H), 8.83 (d, *J* = 4.8 Hz, 1H), 8.77 (s, 1H), 8.70 (s, 1H), 8.46–8.39 (m, 2H), 8.35–8.28 (m, 2H), 8.16 (t, *J* = 8.2 Hz, 2H), 8.09 (t, *J* = 7.5 Hz, 1H), 7.96 (d, *J* = 7.7 Hz, 1H), 7.80 (t, *J* = 6.6 Hz, 1H), 7.68–7.63 (m, 1H), 7.63–7.56 (m, 2H). ^13^C NMR: not recorded due to insufficient complex solubility. IR (KBr, cm^–1^): 2021 (vs), 1915 (vs) and 1893 (vs) ν(C≡O); 1611 (m), ν(C=N) and ν(C=C). HRMS (ESI) (*m*/*z*): [M – Cl]^+^ calcd for [C_32_H_19_N_3_O_3_Re]^+^ 680.0984. Found 680.0986. Anal. Calcd for C_32_H_19_ClN_3_O_3_Re·2H_2_O (751.17 g·mol^–1^): C, 51.16; H, 3.09; N, 5.59. Found: C, 51. 34; H, 2.83; N, 5.45.

### Physical Measurements

High-resolution mass spectrometry analyses were performed on a Waters Xevo G2 Q-TOF mass spectrometer (Waters Corporation) equipped with an ESI source operating in positive-ion modes. Elemental analysis was recorded on a Vario EL Cube apparatus. IR spectra were measured using a Nicolet iS5 Fourier transform infrared (FTIR) spectrophotometer (4000–400 cm^–1^) in the form of KBr pellets. Absorption spectra were recorded on a Thermo Scientific Evolution 220 (solution) and a Jasco V-570 (solid state as a film deposited on a glass substrate and as blends with poly(*N*-vinylcarbazole) (PVK): 2-(4-*tert*-butylphenyl)-5-(4-biphenylyl)-1,3,4-oxadiazole (PBD) on a glass substrate). NMR spectra were recorded on a Bruker Avance 400 NMR spectrometer (298 K) using DMSO-*d*_6_ as the solvent. Resonance frequencies of 400 and 100 MHz were used for ^1^H NMR and ^13^C NMR spectra, respectively.

Cyclic voltammetry (CV) experiments were carried out in a classic three-electrode system. A Pt wire and a Pt spiral were used as working and counter electrodes, respectively. A leakless Ag/AgCl electrode (eDAQ) was used as a reference electrode. The electrode potential was calibrated against ferrocene. Bu_4_NPF_6_ in CH_2_Cl_2_ with a concentration of 0.1 mol·dm^–3^ was used as a supporting electrolyte. Measurements were performed with an Autolab PGSTAT 100N with a scan rate of 50 mV·s^–1^.

### Photo- (PL) and Electroluminescence (EL) spectra

Steady-state luminescence spectra were recorded on an FLS-980 fluorescence spectrophotometer equipped with a 450 W Xe lamp and a high-gain photomultiplier PMT + 500 nm (Hamamatsu, R928P) detector. The emission spectra at 77 K were registered in a butyronitrile rigid matrix. The PL lifetime measurements were performed with the time-correlated single photon counting (TCSPC) method. The TCSPC measurements were carried out in optically diluted solutions using a picosecond laser diode (EPL 405 nm) as the excitation light source and PMT (Hamamatsu, R928P, Japan) as a detector. The IRF was designated using the Ludox solution. Decay curves were fitted using deconvolution fit analysis. For samples with long-lived phosphorescence, PL lifetime measurements were performed with the multichannel scaling (MCS) method, and a 60 W microsecond Xe flash lamp was used for sample excitation. The quantum yields of fluorescence were determined by the absolute method using the integrating sphere with the solvent (for argon-saturated solution samples) or the Spectralon reflectance standard (for powdered samples) as blanks. FLS-980 software was used to perform the emission correction and calculation of the quantum yield. To collect electroluminescence (EL) spectra, a precise voltage supply (Gw Instek PSP-405) with the sample fixed to an *XYZ* stage was applied. Light from the OLED device was collected through a 30 mm lens, focused on the entrance slit (50 μm) of a monochromator (Shamrock SR-303i), and detected using a charge-coupled device (CCD) detector (Andor iDus 12305). Typical acquisition times were equal to 10 s. The prealignment of the setup was done using a 405 nm laser.

Singlet oxygen generation efficiency was determined by monitoring the photooxidation of 1,3-diphenylisobenzofuran (DPBF) sensitized by Re(I) complexes in DMSO. Singlet oxygen quantum yields (Φ_Δ_) of Re(I) complexes were estimated using [Ru(bipy)_3_](PF_6_)_2_ as the standard (Φ_ΔO_2__ = 0.66 in DMSO).^49,50^

### Femtosecond Transient Absorption (fsTA)

Femtosecond TA spectra were recorded using a pump–probe transient absorption spectroscopy system (Ultrafast Systems, Helios) described in our previous work.^35^ TA experiments were carried out for the solution samples (in CHCl_3_, MeCN, and DMSO), stirred during the experiments to avoid photoproduct interference. The absorbance range was equal to 0.20–0.70 in the pumping wavelengths (corresponding to a concentration of 2.5 × 10^–4^ mol·dm^–3^). The 405 and 355 nm pump pulses were used to excite the samples. Transient absorption data were analyzed using Surface Xplorer (Ultrafast Systems) and OPTIMUS software.^51,52^ A more detailed description of the fsTA studies is given in Figures S20–S25 in the Supporting Information (SI).

### Computational Details

Theoretical calculations were performed using the Gaussian 09 program package^53^ at the density functional theory (DFT) or time-dependent (TD)-DFT level with the PBE1PBE^54,55^ hybrid exchange–correlation functional, the def2-TZVPD basis set for rhenium, and the def2-TZVP basis set for other elements.^56,57^ In all calculations, the acetonitrile solvent environment was simulated using the polarizable continuum model (PCM).^58,59^

## Results and Discussion

### Synthesis, Molecular Structures, and Stability

The complexes [ReCl(CO)_3_(4′-An-terpy-κ^2^N)] (An = 9-anthryl (**1**) and 2-anthryl (**2**)) were obtained in satisfactory yields by reacting 4′-An-terpy with [Re(CO)_5_Cl] under reflux in toluene. The identity and purity of **1** and **2** were determined by NMR spectroscopy (Figures S1 and S2), FTIR technique (Figures S3 and S4), HRMS (Figures S5 and S6), and elemental analysis. ^1^H NMR spectra of complexes **1** and **2** demonstrate the splitting of signals for the protons of the peripheral pyridine rings, which confirms the κ^2^N coordination of the 4′-An-terpy ligand. For **1**, the full assignment of ^1^H and ^13^C NMR signals was provided with the aid of two-dimensional techniques ^1^H–^1^H correlated spectroscopy (COSY), ^1^H–^13^C heteronuclear multiple quantum correlation (HMQC), and ^1^H–^13^C heteronuclear multiple bond correlation (HMBC) (Figures S1 and S2). The facial geometry of carbonyl ligands in **1** and **2** was evidenced by solid-state IR spectroscopy, where the characteristic band pattern for CO vibrations was observed. Namely, a sharp intense C≡O stretching band (2024 cm^–1^ for **1** and 2021 cm^–1^ for **2**) was present in the spectra along with two poorly resolved bands in the lower energy range (1927 and 1897 cm^–1^ for **1** and 1916 and 1893 cm^–1^ for **2**; Figures S3 and S4).^1−8,35^

Both Re(I) complexes are stable in solution (Figure S7) and show acceptable photostability (Figure S8).

Regarding the thermal properties, the complexes showed melting temperatures of 177 and 217 °C for **1** and **2**, respectively, as detected using the differential scanning calorimetry (DSC) technique. These values are relatively high. After rapid cooling, the compounds form a stable amorphous phase with glass transition registered during the second heating scan at 206 and 198 °C for **1** and **2**, respectively.

### Ground-State Properties

To determine the impact of the anthryl molecular geometry on the ground-state properties of [ReCl(CO)_3_(4′-An-terpy-κ^2^N)], complexes **1** and **2** were investigated by cyclic voltammetry and absorption spectroscopy.

**Figure 1:**
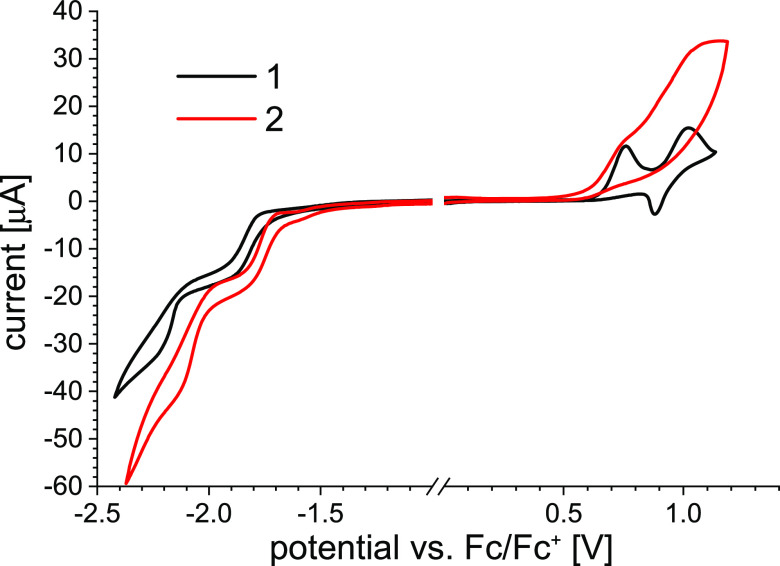
Cyclic voltammetry curves of complexes **1** and **2**. Concentration: 2 mmol·dm^–3^, electrolyte: Bu_4_NPF_6_/CH_2_Cl_2_, and scan rate: 50 mV·s^–1^.

The basic electrochemical parameters, such as oxidation onset potential (*E*_ox onset_), reduction onset potential (*E*_red onset_), ionization potential (IP), and electron affinity (EA) are summarized in Table 1. Cyclic voltammetry curves of **1** and **2** are shown in Figure 1. Bu_4_NPF_6_/CH_2_Cl_2_ was chosen as an electrolyte due to the good solubility of the studied compounds in this solvent. Both complexes present multistage oxidation and reduction characteristics.

**Table 1:** Electrochemical Properties of Complexes **1** and **2**

	*E*_ox onset_^a^ (V)	*E*_red onset_^b^ (V)	IP^c^ (eV)	EA^d^ (eV)	Eg_el_^e^ (eV)	Eg_opt_^f^ (eV)
**1**	0.67	–1.76	5.77	3.34	2.43	2.61
**2**	0.61	–1.69	5.71	3.41	2.30	2.46

a *E*_ox onset_: oxidation onset potential.

b *E*_red onset_: reduction onset potential.

c IP: ionization potential estimated from the equation IP = |e^–^|(5.1 + *E*_ox onset_).

d EA: electron affinity estimated from the equation EA = |e^–^|(5.1 + *E*_red onset_).

e Eg_el_: electrochemical band gap estimated from the equation ΔEg_el_ = *E*_ox onset_ – *E*_red onset_.

f .

By analyzing the values of IP and EA, which are related to the removal of an electron from the highest occupied molecular orbital (HOMO) or the addition of an electron to the lowest unoccupied molecular orbital (LUMO), slight differences between **1** and **2** can be noticed. Most remarkably, complex **2** exhibits a narrower electrochemical band gap (Eg_el_), indicating the increase of the effective coupling in comparison to complex **1**. This is in line with the results of the DFT calculations discussed below and optical energy gap values (Table 1). Similar relationships were also reported for other complexes containing anthracene-based ligands, for example bis(benzo[*h*]quinolinato) Ir(III) complexes^60^ and anthracene-bridged bimetallic ruthenium vinyl complexes.^61^

For both complexes, the shape of CV curves indicates the electrochemical irreversibility of reduction and oxidation processes. A noticeable impact of the anthryl linking mode on the shape of the CV recorded during the oxidation leads to the assumption that the HOMO is largely associated with the anthracene substituent. On the other hand, weaker dependence of the reduction process on the anthryl molecular geometry may indicate that the LUMO of these systems is located predominantly on the terpy core.

**Figure 2:**
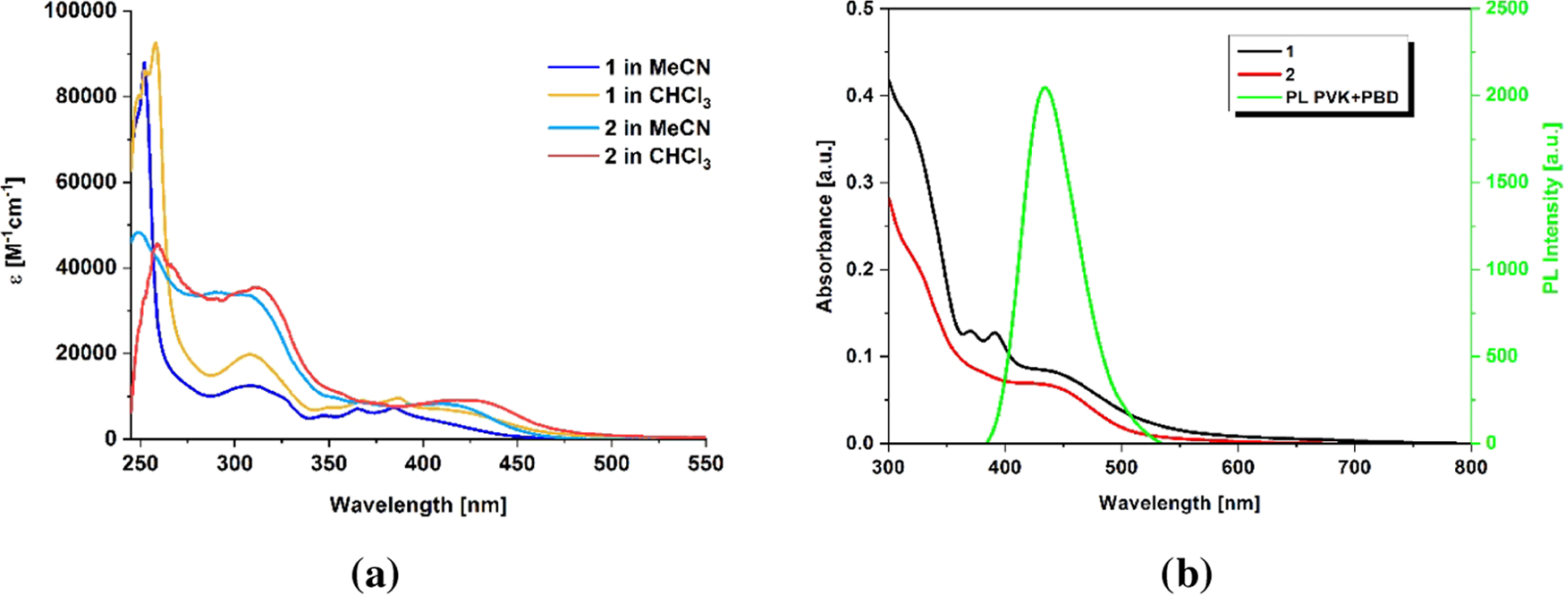
UV–vis absorption spectra of **1** and **2** in solutions (a) and as thin films (b).

The electronic absorption spectra of **1** and **2** were recorded in solvents of different polarities and as thin films (Table S1 and Figure 2).

A comparison of spectral features of **1** and **2** in a lower energy region (330–470 nm) clearly indicates that the anthryl linking mode has a noticeable impact on the absorption properties of [ReCl(CO)_3_(4′-An-terpy-κ^2^N)]. For complex **1**, the absorption in this region is in principle a sum of π_An_ → π*_An_ and Re → terpy* transitions. The structured absorption in the range of 330–390 nm is typical of that of anthracene, while ^1^MLCT (Re → terpy*) charge transfer contribution is visible in the form of a low-energy shoulder above 390 nm.^41,62,63^ These observations are supportive of the weak electronic coupling between the 9-anthryl and terpy units owing to the strong steric hindrance of the rotation about the C–C linker in 9-anthryl-terpy.^48,64^ On the contrary, complex **2** does not show typical anthracene-like absorption with clear vibronic progression. In agreement with a more coplanar geometry and thus more extended delocalization of 2-anthryl-terpy, the ^1^MLCT/^1^IL_An_ absorption band of **2** shifts to lower energies and becomes slightly more intense compared to that of **1**.^48,64,65^ A bathochromic shift of the lowest energy absorption of **1** and **2** caused by replacing acetonitrile with less polar chloroform is due to negative solvatochromism, which is well recognized for rhenium(I) tricarbonyl diimine complexes.^35,66−70^ Intensive bands in the high-energy region of **1** and **2** are attributed to π–π*(terpy) and π–π*(anthracene) transitions (Figure S9).

The impact of the anthryl group directly attached to the terpy core via its 9-position (**1**) and 2-position (**2**) on the electronic structure of [ReCl(CO)_3_(4′-An-terpy-κ^2^N)] was also investigated theoretically at the DFT/PBE0/def2-TZVPD/def2-TZVP level. The geometry optimization shows good agreement with previously reported experimental X-ray data for structurally related Re(I) complexes.^35,68,70−73^ The 9-anthryl and 2-anthryl groups introduced in the terpy ligand do not generate any noticeable structural changes in the {ReClN_2_C_3_} coordination core. The bond lengths and bond angles around the Re(I) ion are almost the same for **1** and **2** (Table S2). Also, the electrochemical data of **1** and **2** are satisfactorily reproduced by the calculated ionization potentials and electronic affinities^74^ (Table S3). The striking difference between **1** and **2** concerns the dihedral angle between the plane of the anthryl group and the central pyridine ring (72° for **1** and 32° for **2**).

**Figure 3:**
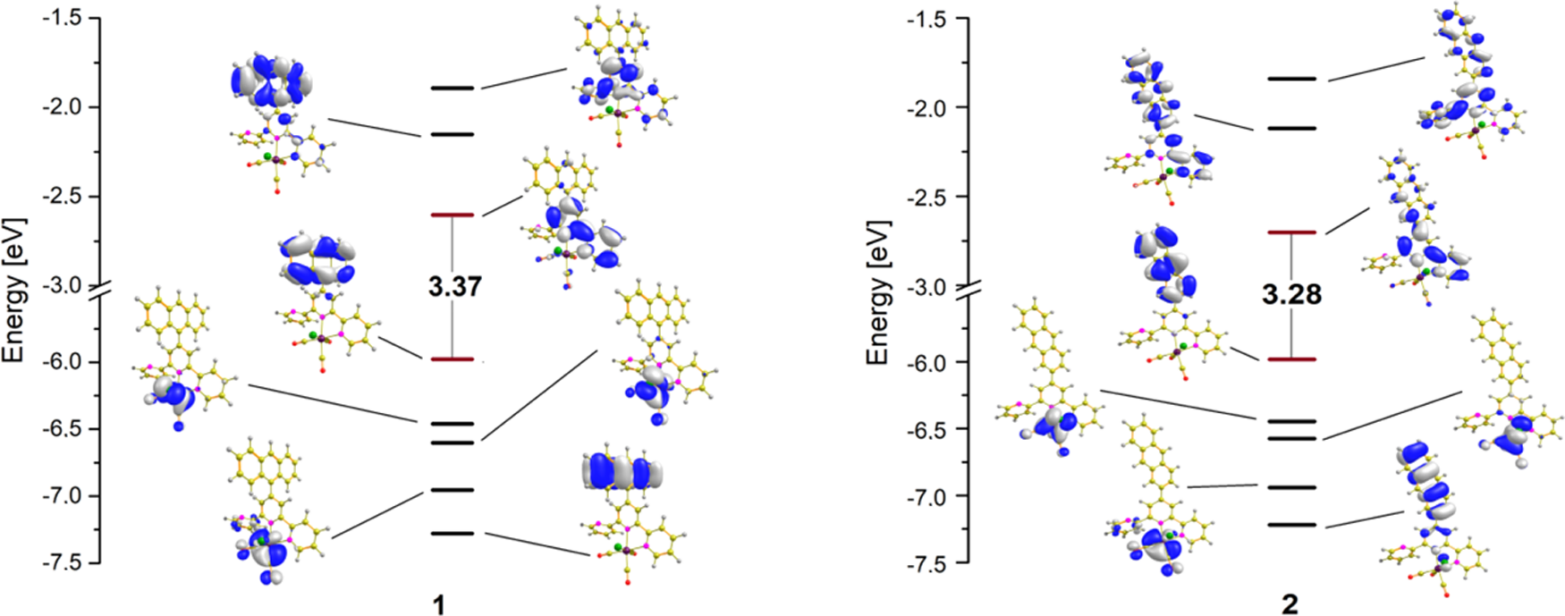
Partial molecular orbital energy-level diagrams for compounds **1** and **2**.

For both compounds, the lowest unoccupied molecular orbital (LUMO) largely resides on the terpy core and the highest occupied molecular orbital (HOMO) is localized on the anthryl substituent, while HOMO–1, HOMO–2, and HOMO–3 orbitals spread over the {Re(CO)_3_Cl} moiety (Figures 3 and S10). Depending on the anthryl linking mode, no clear energy variations are observed for HOMO, HOMO–1, HOMO–2, and HOMO–3 energy levels between compounds **1** and **2**. When considering the LUMO levels of **1** and **2**, the replacement of 9-anthryl by 2-anthryl leads to a slight stabilization of the LUMO and thus causes a decrease in the HOMO–LUMO gap of **2** (3.28 eV) relative to **1** (3.37 eV). These changes can be rationalized as resulting from a more coplanar geometry of 2-anthryl-terpy, which leads to a stronger coupling between the 2-anthryl group and the terpy core. As a result of more extended overlapping orbitals of the 2-anthryl and terpy moieties, molecular orbitals LUMO+1 and LUMO+2 of **2** also have higher contributions of the 2-anthryl orbitals and are better energetically stabilized compared to those of **1**. Most importantly, the HOMO–LUMO energy gaps of compounds **1** and **2** become noticeably lower in comparison to the previously reported [ReCl(CO)_3_(4′-Ar-terpy-κ^2^N)] complex bearing the structural isomer 4′-(phenanthren-9-yl)-2,2′:6′,2″-terpyridine (Figure S11). In contrast to **1** and **2**, the HOMO of [ReCl(CO)_3_(4′-Ar-terpy-κ^2^N)] with 9-phenanthryl-terpy is distributed over the {ReCl(CO)_3_} unit,^35^ which also confirms a pivotal role of the molecular configuration of the appended aryl group. In contrast to linear anthracene, phenanthrene is nonlinear.

To examine the nature of electronic transitions of obtained complexes, time-dependent DFT (TD-DFT) calculations were performed using optimized structures of **1** and **2**. The calculated transitions along with the experimental spectra and natural transition orbitals for the lowest energy band of **1** and **2** are presented in Figure S12. For both complexes, the excitation S_0_ → S_1_ can be assigned to the intraligand π_An_ → π*_terpy_ and π_An_ → π*_An_/π*_terpy_ transition for **1** and **2**, respectively. A twist of the 2-anthryl group, which is smaller than that of the 9-anthryl one, results in an increase in the oscillator strength of the S_0_ → S_1_ transition for complex **2**, which correlates with the stronger absorptivity (ε) of **2** relative to that of **1** in the visible region. The character π_An_ → π*_terpy_/π*_An_ can also be assigned to the S_0_ → S_4_ transition, while the transitions S_0_ → S_2_ and S_0_ → S_3_ are of metal-to-ligand charge transfer (MLCT) nature (Table S4).

### Excited-State Properties

**Figure 4:**
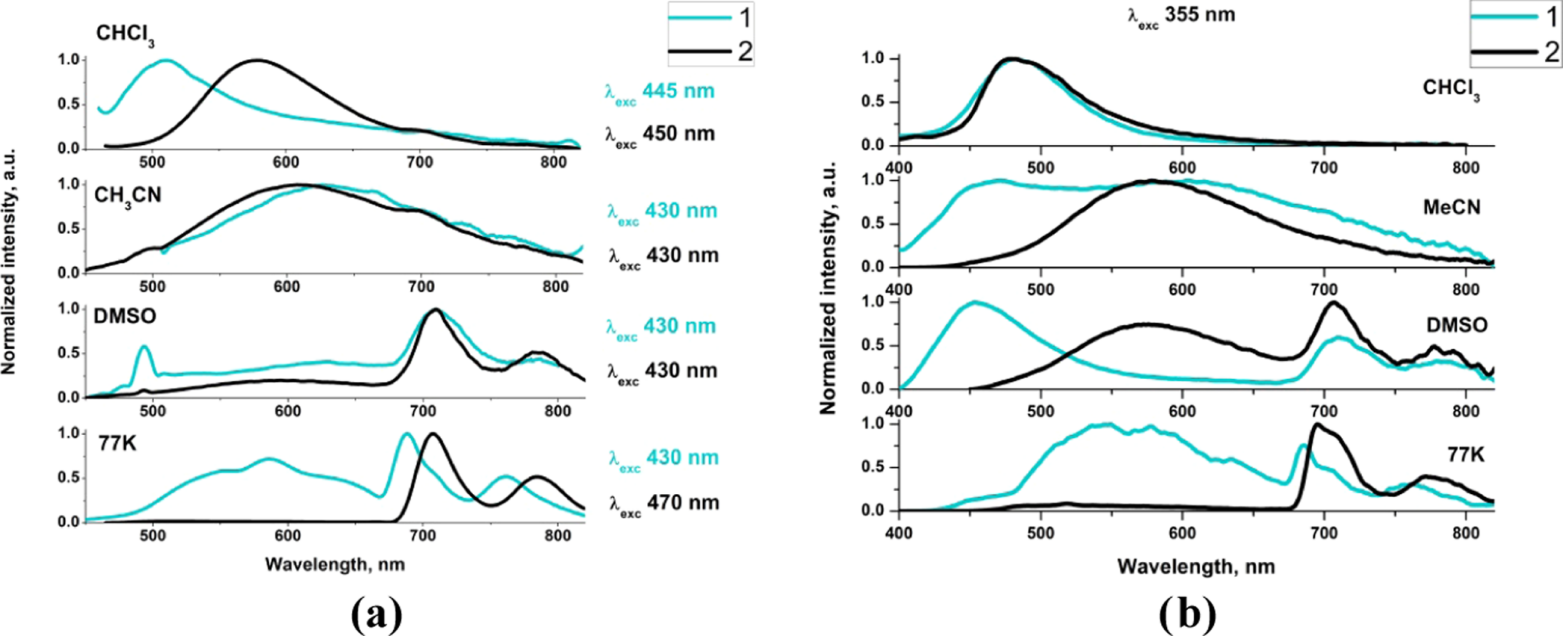
Photoluminescence spectra of **1** and **2** in different environments upon excitation at the red side of the lowest energy absorption (a) and 355 nm (b).

Photoluminescence spectra of **1** and **2** are displayed in Figures 4 and S13–S19. Upon excitation into the red side of the lowest energy absorption, with λ_exc_ > 400 nm, the complexes in acetonitrile solution show broad and unstructured emission with maximum at 627 nm for **1** and 609 nm for **2** (Table 2). With reference to previously reported compounds [ReCl(CO)_3_(4′-Ar-terpy-κ^2^N)] incorporating 2,2′:6′,2″-terpyridines functionalized with 1-naphthyl, 2-naphthyl, and 9-phenanthryl groups, it can be derived that the emission of **1** and **2** in MeCN originates predominately from the ^3^MLCT excited state (Figure S15). However, as shown in Figure S15, the emission bands of **1** and **2** are not completely superimposed over those for [ReCl(CO)_3_(terpy-κ^2^N)] and [ReCl(CO)_3_(4′-Ar-terpy-κ^2^N)] substituted with 1-naphthyl, 2-naphthyl, and 9-phenanthryl. Both maxima and onsets of the ^3^MLCT emission of **1** and **2** are slightly blue-shifted relative to those previously reported, which may suggest some contribution of residual fluorescence due to any incomplete FRET from ^1^IL to ^1^MLCT, as reported previously for other bichromophoric systems.^31,32,75−77^

**Table 2:** Summary of the Photoluminescence Properties of Complexes **1** and **2**^a^

	CHCl_3_				MeCN				DMSO				CH_3_OH:C_2_H_5_OH (77 K)		
	λ_exc_ (nm)	λ_em_ (nm)	τ_av_ (ns)	φ (%)	λ_exc_ (nm)	λ_em_ (nm)	τ_av_ (ns)	φ (%)	λ_exc_ (nm)	λ_em_ (nm)	τ_av_	φ (%)	λ_exc_ (nm)	λ_em_ (nm)	τ_av_ (μs)
**1**	445	511	4.91	3.85	430	627	2.26	4.08	430	^I^627	2.83 ns	0.02	430	^I^587,	^I^3.6
										^II^711	14.28 μs			^II^688, 761	^II^12 069
**2**	450	580	7.30	1.78	430	609	3.97	0.05	430	^I^593	5.75 ns	1.40	470	707, 784	2590
										^II^712	22.71 μs				

a τ_av_: average lifetime of multiexponential fits of decay curves (see Figure S11 in the SI). φ: luminescence quantum yield.

The contribution of the residual fluorescence becomes more noticeable when acetonitrile solutions of **1** and **2** are excited at the blue side of the lowest energy absorption. Irradiation with λ_exc_ = 355 nm predominately populates the ^1^An state. Higher energy excitation results in dual fluorescence–phosphorescence emission for **1** and a significant hypsochromic shift of the structureless emission band for **2** (Figure 4 and Table S5). The replacement of acetonitrile by less polar chloroform induces noticeable changes in the photophysical properties of **1** and **2**. In this case, the emission of **1** excited at 355 nm and λ_exc_ > 410 nm appears at significantly shorter wavelengths (∼510 nm), and it can be assigned to ^1^IL/^1^ILCT fluorescence. The phosphorescence of the ^3^MLCT origin of **1** may be effectively masked by brighter and faster fluorescence or even quenched by the lower-lying triplet state localized on the anthracene moiety, which is well recognized for bichromophoric systems with the anthracene unit.^32,41,78,79^ The structureless emission band of **2** in CHCl_3_ upon excitation λ_exc_ > 410 nm appears at ∼580 nm, which is bathochromically shifted by ∼135 nm compared to the free ligand in CHCl_3_ (Figure S16). In addition, compared to the emission from the ^3^MLCT excited state of **2** in MeCN, it is blue-shifted by ∼30 nm. This allows us to assume that the photophysics of **2** in CHCl_3_ is determined by both the ^1^IL/^1^ILCT fluorescence and ^3^MLCT phosphorescence. The presence of two components is clearly noticeable upon excitation of **2** in CHCl_3_ at 410 nm (Figure S14). In DMSO, two emission bands are observed for both complexes **1** and **2** excited with λ_exc_ > 410 nm. The broad and weak component at higher energies falls in the range of ^3^MLCT phosphorescence, while the structured emission at longer wavelengths (>680 nm) can be safely assigned to ^3^An. Regarding excited-state lifetimes of ^3^MLCT and ^3^An, it can be assumed that they decay independently, there is no evidence of formation of the triplet-state equilibrium between the anthracene and {ReCl(CO)_3_(terpy-κ^2^N)} chromophores.

Higher energy excitation (λ_exc_ = 355 nm) of **1** in DMSO results in dual fluorescence–phosphorescence ligand-centered emission for **1**, while the emission spectrum of **2** still shows both ^3^MLCT and ^3^An components. However, compared to the spectra measured for lower energy excitation (λ_exc_ = 430 nm), the relative contribution of the ^3^MLCT component is considerably enhanced. Most importantly, the anthracene-related room-temperature phosphorescence is rarely observed. For the first time, this was reported for Pt(II) bisacetylide complexes,^80^ and its presence was rationalized by the enhanced effect of the heavy atom on anthracene.

To obtain further insights, the photoluminescence of [ReCl(CO)_3_(4′-An-terpy-κ^2^N)] was examined at a low temperature (77 K). The frozen-state emission spectra also revealed noticeable differences between **1** and **2** (Figure 4). For compound **1**, the emission spectrum at 77 K consists of two components: a broad and slightly structured emission band in the range of 480–670 nm, which overlaps with the phosphorescence of [ReCl(CO)_3_(4′-Ar-terpy-κ^2^N)] incorporating 2,2′:6′,2″-terpyridines functionalized with 1-naphthyl, 2-naphthyl, and 9-phenanthrenyl groups (Figure S17), and a well-structured emission, which is superimposed over the phosphorescence of the free ligand and anthracene (Figure S18). A weak vibronic structure of the high-energy emission is assigned to a small contribution of ^3^IL_terpy_, as reported for Re(I) carbonyl complexes with naphthyl- and phenanthrenyl-substituted terpyridine ligands.^35^ In contrast, complex **2** shows mainly the emission band with a well-resolved vibronic structure in the range of 680–830 nm, originating from the ^3^IL_An_ excited state. Relative to complex **1**, ^3^An emission of **2** appears in a lower energy region, which can be rationalized by the negligible population of the ^3^MLCT excited state, contrary to complex **1** (Figure 4).

**Figure 5:**
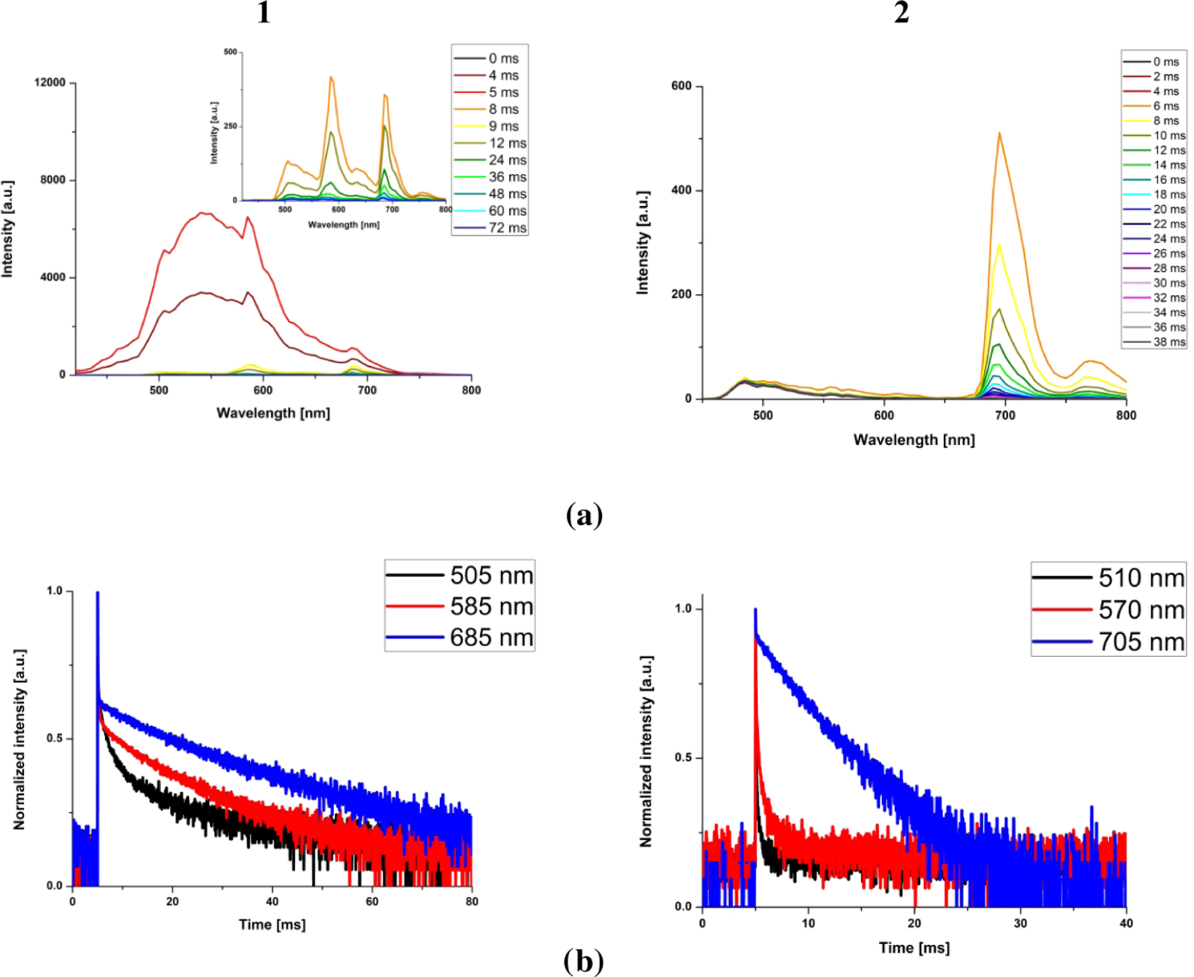
(a) Time-resolved emission spectra for **1** and **2** in the BuCN rigid matrix at 77 K (excitation wavelength: 405 nm and time window: 80 ms for **1**, and excitation wavelength: 405 nm and time window: 20 ms for **2**). (b) Decay curves for the maxima of emission bands observed in time-resolved emission spectra for **1** and **2** in the BuCN rigid matrix at 77 K.

The formation of the ^3^MLCT state upon excitation of **2** was evidenced more clearly by time-resolved emission spectra (TRES) recorded at 77 K. As demonstrated in Figure 5, the ^3^MLCT excited state of **2** almost immediately undergoes triplet–triplet energy transfer into ^3^IL localized on the 2-anthryl-terpy ligand. Another striking difference between **1** and **2** concerns the relative contributions of the residual fluorescence, which is significantly larger for complex **1**.

The nature of the triplet excited state of [ReCl(CO)_3_(4′-An-terpy-κ^2^N)] was also investigated theoretically. In this regard, structures of **1** and **2** were optimized in their triplet states (T_1_). The character of the lowest energy triplet excited state was assigned using the spin density surfaces generated from the lowest energy triplet state, and the phosphorescence energies were determined as the difference between the ground singlet and triplet states Δ*E*_T_1_–S_0__.

**Figure 6:**
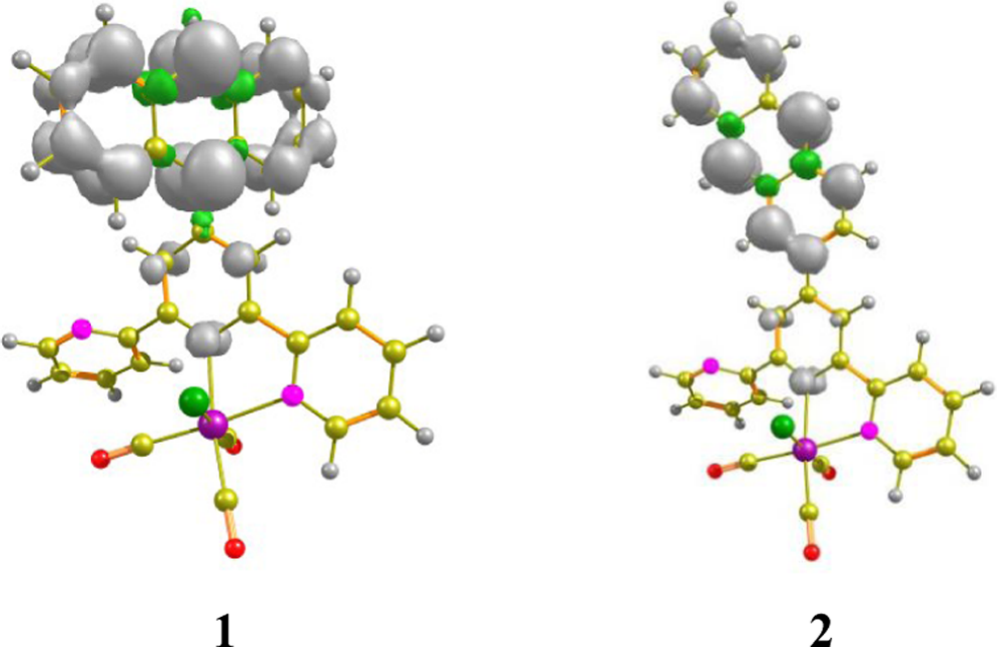
Spin density surface plots for **1** and **2**. Gray and green colors show regions of positive and negative spin density values, respectively (see also Table S6 in the SI).

As shown in Figure 6, the spin density surfaces of both **1** and **2** were distributed on the anthryl substituent and central pyridine of terpy, supporting the ^3^An/^3^(An-terpy) character of the lowest energy triplet state. Calculated phosphorescence energies, 905 nm for **1** and 886 nm for **2**, correlate well with the lowest energy shoulder of the structured triplet emission band of anthracene.^81^

### Femtosecond Transient Absorption—The Nature of the Lowest Triplet-State and Excited-State Dynamics

The nature of the lowest triplet-state and excited-state dynamics of **1** and **2** was investigated by applying pump–probe transient absorption with femtosecond (fsTA) resolution. The measurements were performed for solutions of **1** and **2** in CHCl_3_, MeCN, and DMSO upon excitation at 355 nm, as well as for **1** and **2** in CHCl_3_ excited at 405 nm. After photoexcitations at 355 and 405 nm, both ^1^An and ^1^MLCT excited states are simultaneously populated, but a higher ^1^An/^1^MLCT population ratio is expected in the case of 355 nm excitation.

**Figure 7:**
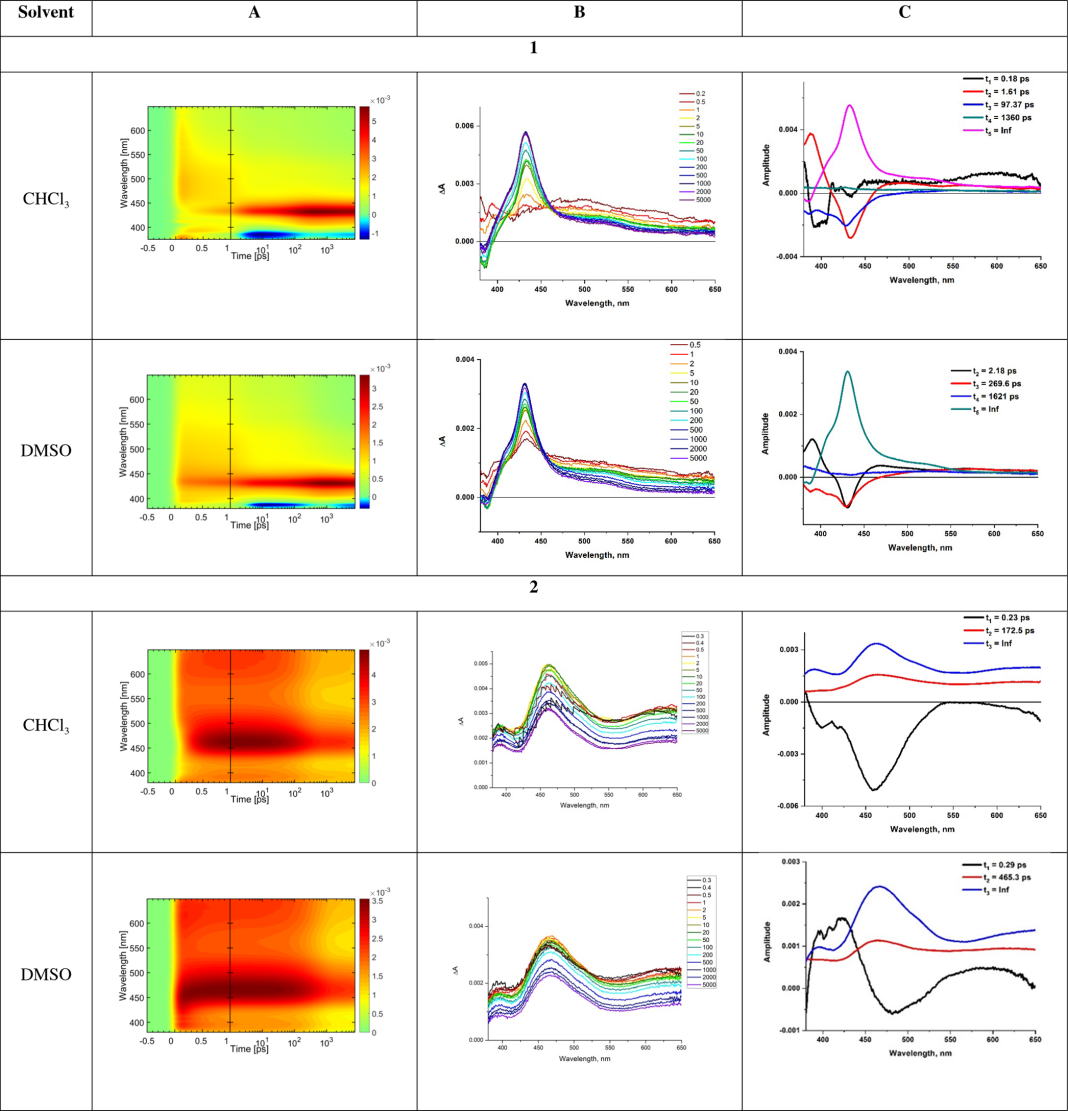
Summary of fsTA measurements for complexes **1** and **2** excited at 355 nm: fsTA 2D maps (A), TA spectra at selected time delays (B), and decay-associated spectra (DAS) (C) (see also Figures S21–S25).

The results of fsTA experiments of complexes **1** and **2** together with global fitting analysis are summarized in Figures 7 and S21–S25.

**Figure 8:**
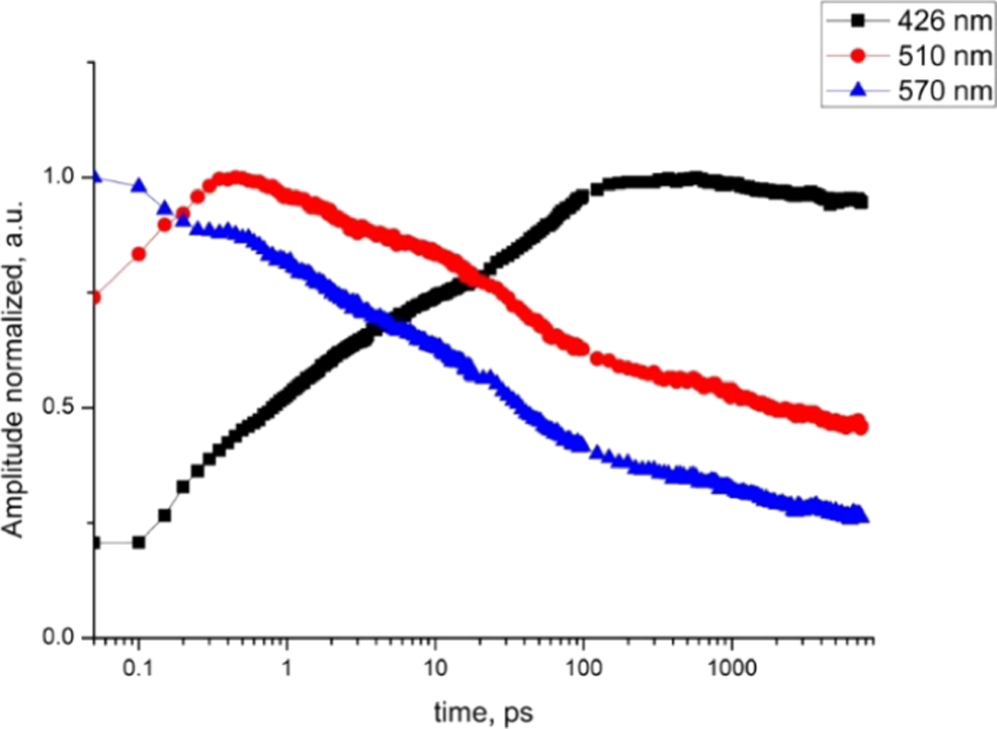
Time traces for representative wavelengths of 426 nm (maximum of T_1_(^3^An) → T*_n_*(^3^An) ESA), 510 nm (red edge of ^3^LMCT ESA), and 570 nm (maximum of T_2_(^3^An) → T*_n_*(^3^An) ESA) for **1** in MeCN excited at 355 nm.

The most intense excited-state absorption (ESA) of **1** in the range of 410–460 nm is a typical spectral feature corresponding to the T_1_ → T*_n_* transitions of anthracene.^46,47,65,78,82,83^ It is the only signal at longer time delays, confirming that the lowest triplet state is localized on the anthracene chromophore. The ESA band starts rising almost immediately after photoexcitation and persists up to the end of the delay stage, as the ^3^An lifetime is much longer than the maximum pump–probe time delay of the fsTA setup.^81^ Regarding the spectral changes occurring at early time delays and previous reports on related systems,^37,47,84,85^ we can assume that the final spectral component (^3^An) of **1** is formed via two paths, ^1^MLCT → ^3^MLCT → ^3^An and S_1_(^1^An) → T_2_(^3^An) → T_1_(^3^An). The population of ^3^An via two channels is clearly evidenced by the biphasic kinetics of growth displayed on time trace at 426 nm (see Figures 8, S23, and S24 showing time traces for selected wavelengths along with fitting curves obtained via global analysis). The ^3^MLCT state is represented by ESA bands with maxima at ∼385 and ∼495 nm, in agreement with the results for the parent chromophore [ReCl(CO)_3_(terpy-κ^2^N)]^37^ (Figure S25). The signal in the UV region is attributed to the absorption of polypyridine anion radicals, while ESA in the visible part corresponds to ligand-to-metal charge transfer (LMCT, Cl/L^•–^ → Re) transitions.^37,84,85^ As reported by Yang,^47^ spectral features attributable to T_2_(^3^An) → T*_n_*(^3^An) transitions are expected in the range of 500–600 nm, partly overlapping with the ESA of LMCT.

**Scheme 2:**
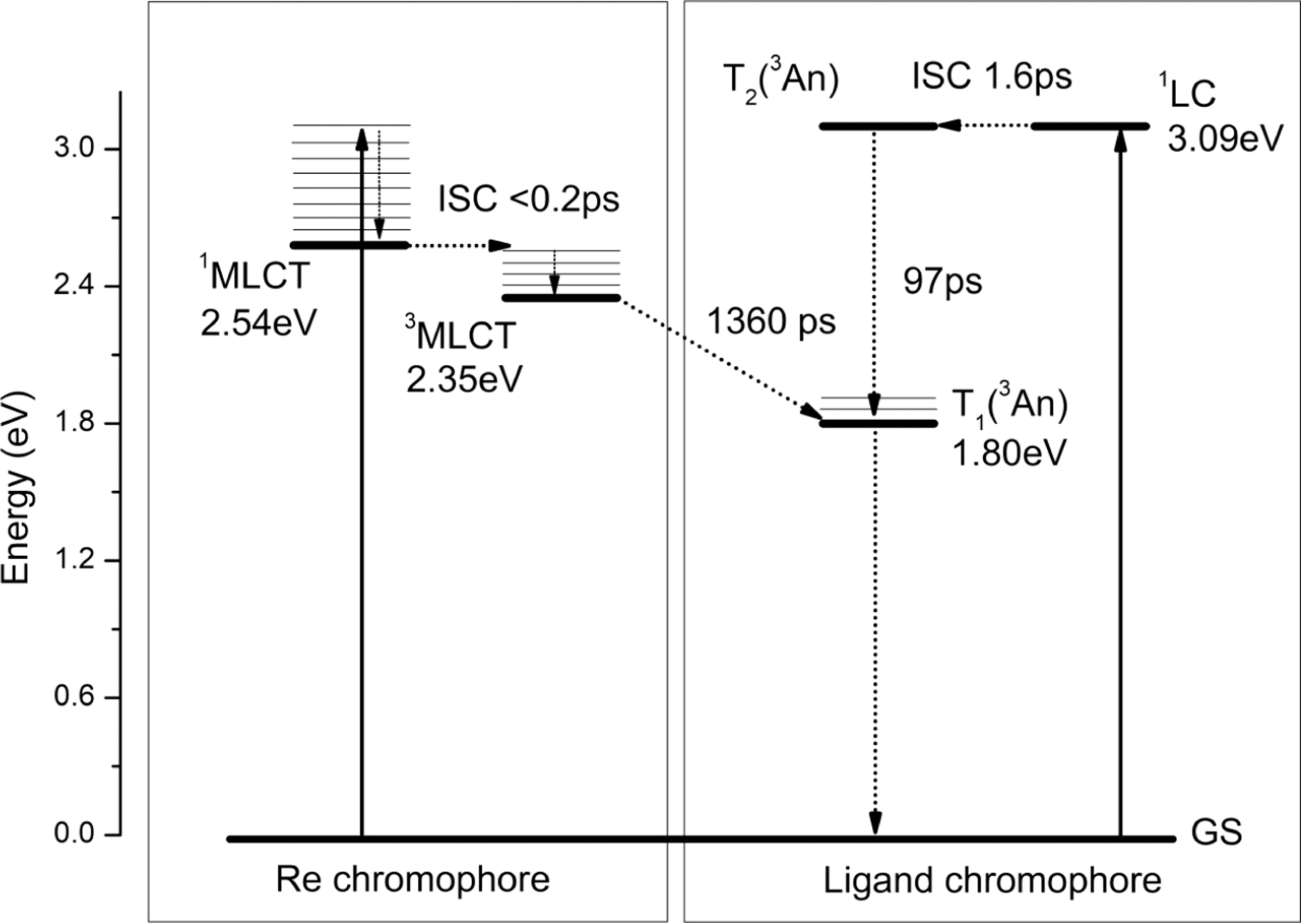
Representative Energy-Level Diagram along with Photophysical Processes Occurring upon Photoexcitation of **1** The energies of ^1^LC and ^1^MLCT were estimated from onsets of the lowest energy absorption bands of the corresponding ligand and parental [ReCl(CO)_3_(terpy-κ^2^N)] in CHCl_3_, respectively, while ^3^LC and ^3^MLCT energies were obtained from onsets of 77 K phosphorescence of the organic ligand and the room-temperature emission of [ReCl(CO)_3_(terpy-κ^2^N)] in CHCl_3_, respectively.

Time constants determined from global fit analysis are summarized in Table 3, and decay-associated spectra (DAS) are shown in column C in Figure 7. The ultrafast intersystem crossings, which occur in the time range shorter than the instrument response, are not included in Table 3. Besides the discarded ultrafast time constants, which can be safely assigned to the ultrafast ISC from the initially populated ^1^MLCT to ^3^MLCT,^37,84,85^ four components are involved in the fsTA kinetics of **1**. The lifetimes *t*_2_, *t*_3_, and *t*_4_ can be attributed to S_1_(^1^An) → T_2_(^3^An), T_2_(^3^An) → T_1_(^3^An), and ^3^MLCT → ^3^An processes, respectively. DAS_5_ (with *t*_5_ = inf) corresponds to the absorption spectrum of the fully relaxed lowest triplet state ^3^An, which does not completely decay within the time scale of the measurement. The photophysical processes occurring upon photoexcitation of **1** are summarized in Scheme 2.

**Table 3:** Summary of the Time Constants (ps) from Global Fit Analysis

		405 nm	355 nm		
	*t* (ps)	CHCl_3_	CHCl_3_	MeCN	DMSO
**1**	*t*_1_		0.18		
	*t*_2_	1.53	1.61	1.93	2.18
	*t*_3_	92.9	97.37	52.0	269.6
	*t*_4_	1964	1360	1730	1621
	*t*_5_	inf	inf	inf	inf
**2**	*t*_1_	0.58	0.23	0.18	0.29
	*t*_2_	168.8	172.5	105.1	465.3
	*t*_3_	inf	inf	inf	inf

Excitation of **2** at both 355 and 405 nm results in an instant appearance of excited-state absorption (ESA) with the main peak at 460 nm and a minor one at 640 nm. Within about 2–5 ps, the intensity of the ESA at 460 nm increases and then starts to slowly decrease. The rise of the higher energy band is accompanied by a slight intensity decrease of the minor peak within this time scale. In analogy to **1**, the dominant ESA corresponding to the T_1_ → T*_n_* transitions of the anthracene unit does not completely decay within the time scale of the measurements, but a more planar geometry of 2-anthryl-terpy, and thus stronger electronic communication between the anthracene and {ReCl(CO)_3_(terpy-κ^2^N)} chromophores, facilitates substantial population of the ^3^An triplet state. Compared to **1**, however, T_1_ → T*_n_* absorption band shows noticeable red shift and broadening, which can be attributed to the stronger electronic coupling between An and terpy moieties in complex **2**.

For the best fitting of transient spectra kinetics of **2** upon excitation at 355 and 405 nm, three time constants were required (Table 3). Over the course of the first time constant (Table 3), the vibrationally hot ^3^An excited state is formed. DAS_2_ (corresponding to *t*_2_), which is positive in the region corresponding to the fully relaxed lowest triplet state ^3^An, reflects the vibrational relaxation of the lowest triplet state ^3^An, comprising reorganization within the [ReCl(CO)_3_(4′-An-terpy-κ^2^N)] bichromophore and interacting solvent molecules. DAS_3_ with infinite lifetime corresponds to ground state recovery.

### Abilities for ^1^O_2_ Generation and Light Emission under the External Voltage

**Figure 9:**
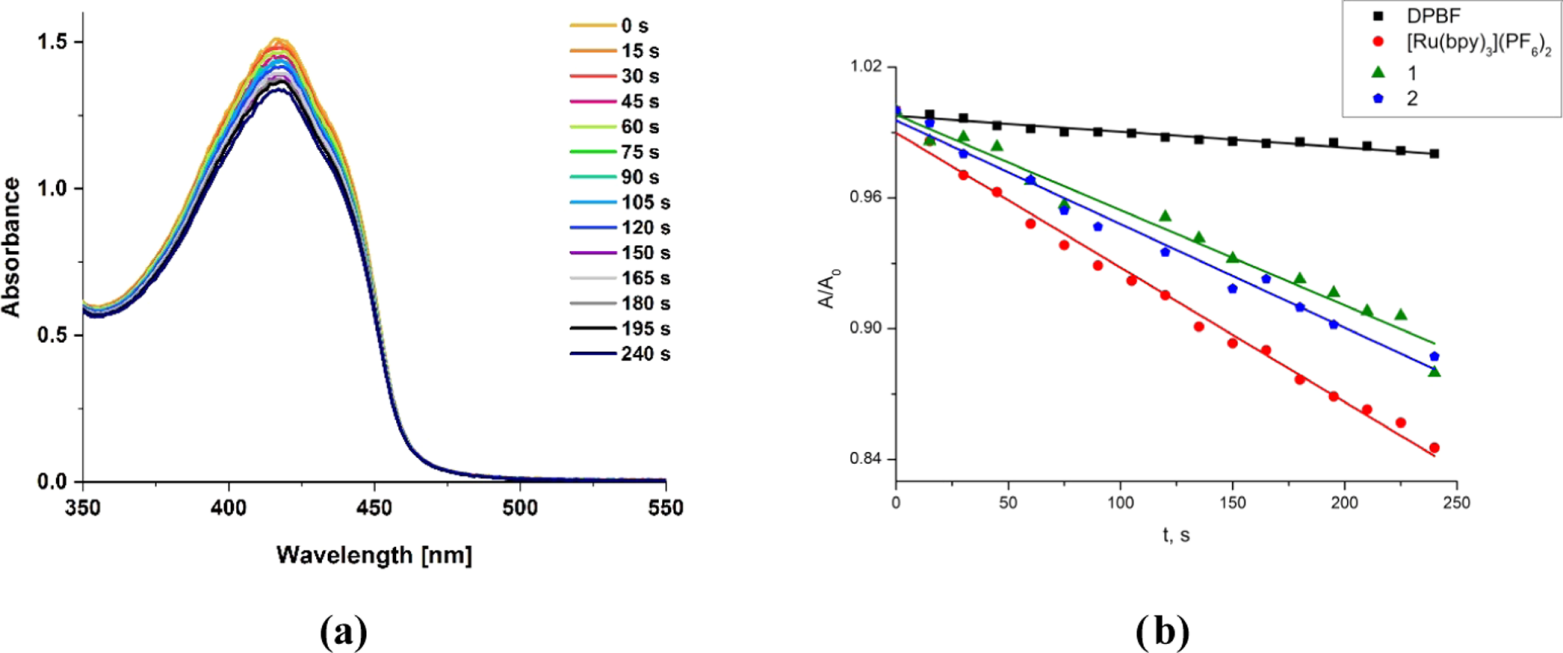
UV–vis absorption spectra of diphenylisobenzofuran (DPBF) in DMSO (*c*: 50 μM) treated with complex **1** (*c*: 50 μM) upon exposure to visible light at 420 nm recorded over 240 s (a) and relative changes in the absorbance of DPBF at 417 nm (*A*/*A*_0_) with time (b).

Since both complexes have long excited-state lifetimes (14 μs for **1** and 22 μs for **2** in DMSO), it can be anticipated that they are suitable for transferring the excited triplet-state energy to molecular oxygen, generating singlet oxygen. The ability of Re(I) complexes for photosensitized generation of singlet oxygen was examined by an indirect method with the use of diphenylisobenzofuran (DPBF), which is highly sensitive to ^1^O_2_. Reacting with ^1^O_2_, diphenylisobenzofuran forms endoperoxide, which spontaneously decomposes to 1,2-dibenzoylbenzene.^49,86−88^ As shown in Figure 9, both complexes **1** and **2** exhibit singlet oxygen sensitizing ability. The insignificantly enhanced singlet oxygen sensitizing ability of **2** (Φ_ΔO_2__ = 0.45) in relation to **1** (Φ_ΔO_2__ = 0.42) correlates with its longer triplet excited-state lifetime in DMSO at room temperature (Table 2). For both complexes, the values of Φ_ΔO_2__ are lower than that for anthracene (0.57) but ∼2 times greater than those for free ligands (0.21 for 9-anthryl-terpy and 0.23 for 2-anthryl-terpy; Figures S27 and S28). Relative to the efficient [Ru(bipy)_3_](PF_6_)_2_ photosensitizer (Φ_ΔO_2__: 0.66), however, the absorbance plots of DPBF in the presence of **1** and **2** display lower slopes, indicating their weaker abilities for ^1^O_2_ generation.

To perform preliminary tests of [ReCl(CO)_3_(An-terpy-κ^2^N)] ability for light emission induced by voltage, two types of diodes with structures of ITO/PEDOT:PSS/complex/Al and ITO/PEDOT:PSS/PVK:PBD:complex/Al were fabricated. The matrix consisting of poly(9-vinylcarbazole) (PVK, 50 wt %) and (2-*tert*-butylphenyl-5-biphenyl-1,3,4-oxadiazole) (PBD, 50 wt %) was applied due to its effective charge carrier transport of ambipolar character. Moreover, diodes with such a guest–host configuration were also utilized in our previous investigations concerning Re(I) complexes.^35−37,67,68,70,71,73,82,89^ As can be seen in Figure 3b, absorption of **1** and **2** partially overlaps with the emission spectrum of the host (matrix PVK:PBD), thus Förster energy transfer can be expected.^90^ However, only the diode with complex **1** as an active layer showed weak emission with the maximum electroluminescence (EL) band (λ_EL_) at 715 nm under 20 V, contrary to the device based on **2**, which was nonemissive. With reference to the discussion in the previous section, this weak emission can be assigned to anthracene phosphorescence.

**Figure 10:**
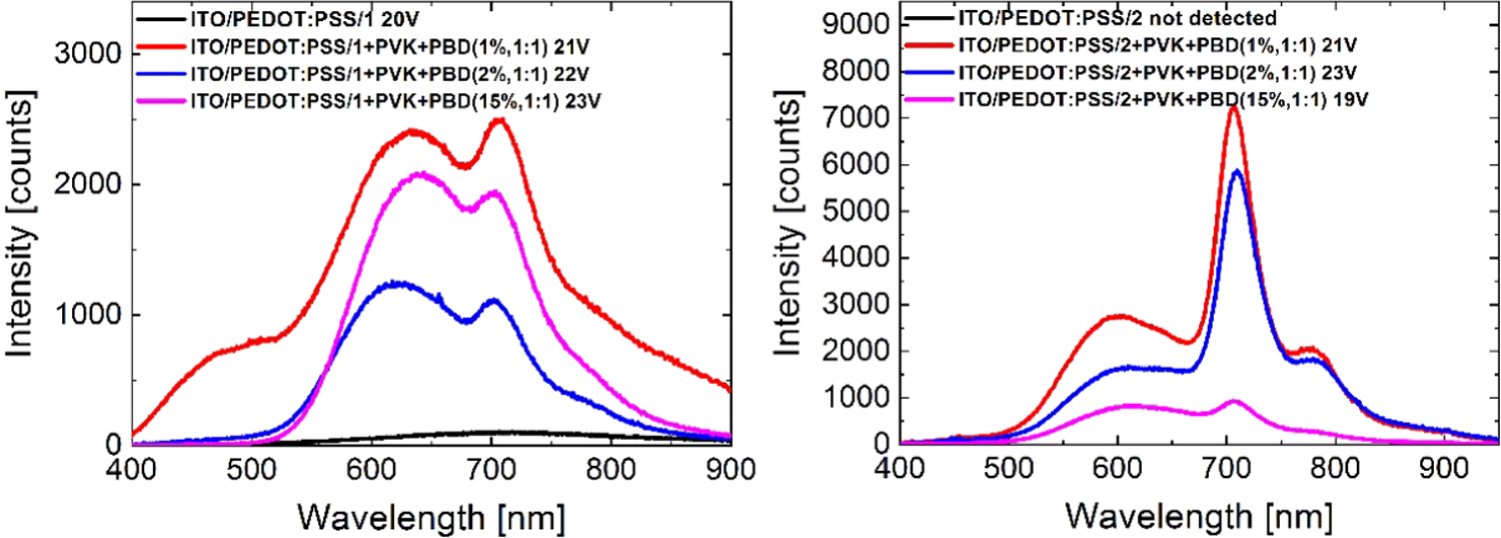
Electroluminescence spectra of diodes based on investigated Re(I) complexes with anthryl groups.

In the case of diodes with guest–host structures, the emission was dependent on the applied voltage, and an increase of the applied voltage resulted in an increase in the light emission. Nevertheless, rather high voltages were required to detect light emission (Figure 10).

Most importantly, the electroluminescence (EL) band of guest–host diodes covers a broad range from the visible (500 nm) to near-infrared region (750–900 nm), with λ_EL_ located at about 638 and 705 nm for **1** and 600, 705, and 778 for **2**. The addition of another component, emitting blue light from 400 to 500 nm, may yield a diode, which emits white light (WORED). The EL spectra are similar to the photoluminescence spectra of **1** and **2** registered in DMSO at RT and a rigid matrix at 77 K in BuCN (Figure 4). Regarding the complex content, the addition of 1 wt % gives the most intense emission. In turn, taking into consideration the anthryl linking mode, significantly more intense EL showed a device based on the complex with 2-anthryl substituent (**2**), which well correlates with the higher PL quantum yield of **2** in DMSO relative to **1**. To the best of our knowledge, such broad and structured emission, ranging to 900 nm with two or three maxima, was observed for the first time in diodes based on [ReCl(CO)_3_(4′-Ar-terpy-κ^2^N)] compounds.^35−37,67,68,70,71,73,82,89^ Indeed, diodes reported previously by our research group, Re(I) complexes based on 2,2′:6′,2″-terpyridines substituted with conjugated aryl groups such as 1-naphthyl, 2-naphthyl, 9-phenanthrenyl, and 1-pyrrenyl applied as guests in the PVK:PBD matrix (1, 2, and 15 wt %), exhibited λ_EL_ in ranges of 595–600, 595–610, 585–610, and 640–650 nm, respectively.^35^ In addition, devices with molecularly dispersed Re(I) complexes bearing electron-donating amine units also showed emission in a significantly narrower spectral range.^72,91^

## Conclusions

In summary, in-depth studies of the electrochemical and optical properties of two Re(I) complexes with 2,2′:6′,2″-terpyridines (terpy) substituted with the 9-anthryl (**1**) and 2-anthryl (**2**) confirmed a noticeable impact of the anthryl linking mode on the ground- and excited-state properties of resulting [ReCl(CO)_3_(4′-An-terpy-κ^2^N)]. As a result of the more extended overlapping orbitals of 2-anthryl and terpy moieties, the absorption band of **2** is shifted to lower energy and becomes slightly more intense compared to that of **1** with a clearly twisted geometry of 9-anthryl-terpy. The stronger electronic coupling between the anthracene and {ReCl(CO)_3_(terpy-κ^2^N)} chromophores also facilitates the population of the ^3^An triplet state. Following the femtosecond transient absorption, the excitation of **2** results in an instant appearance of excited-state absorption (ESA) corresponding to the T_1_ → T*_n_* transitions of the anthracene unit, while the ^3^An ESA of **1** is formed significantly slower, and its rise occurs via two independent channels. It is worth noticing that complexes **1** and **2** are rare examples that show anthracene-related room-temperature phosphorescence. However, although complexes **1** and **2** in DMSO excited with λ_ex_ > 410 nm show both ^3^MLCT and ^3^An emission bands, there was no evidence of formation of the equilibrium between the triplet states localized on anthracene and {ReCl(CO)_3_(terpy-κ^2^N)} chromophores. The excited states ^3^MLCT and ^3^An decay independently with different excited-state lifetimes. The presence of both ^3^MLCT and ^3^An emissions was also observed in the electroluminescence band of guest–host diodes based on investigated Re(I) complexes. Having long excited-state lifetimes in DMSO, the examined carbonyls were found to be suitable for transferring the excited triplet-state energy to molecular oxygen, generating singlet oxygen. We strongly believe that reported findings will be useful for understanding and controlling the excited-state nature of transition metal complexes and thus designing photoluminescent materials with predefined photophysical properties.

## Supporting Information Available

NMR (^1^H, ^13^C, and 2D); IR and HRMS spectra; stability and photostability; UV–vis spectra; DFT and TD-DFT calculations; steady-state and time-resolved emission spectra; spin density surface plots; femtosecond transient absorption spectra; and global fitting results (PDF)
