# Supplementary material for: Impact of the Anthryl Linking Mode on the Photophysics and Excited-State Dynamics of Re(I) Complexes [ReCl(CO)3(4′-An-terpy-κ2N)]
Source: Inorg Chem. 2022 Sep 14;61(38):15070–84. doi: 10.1021/acs.inorgchem.2c02160 (PMC9516691; doi:10.1021/acs.inorgchem.2c02160)
Supplement: Supplementary file 1 — ic2c02160_si_001.pdf [file ic2c02160_si_001.pdf]

# Supporting Information

## Impact of the anthryl linking mode on photophysics and excited state dynamics of Re(I) complexes [ReCl(CO)$_3$(4′-An-terpy-κ$^2$N)]

*Magdalena Małecka [†], Agata Szlapa-Kula [†], Anna M. Maroń [†], Przemyslaw Ledwon [§], Mariola Siwy[‡], Ewa Schab-Balcerzak [†,‡], Karolina Sulowska [|], Sebastian Maćkowski [|], Karol Erfurt [‖], and Barbara Machura *[†]*

[a] *Institute of Chemistry, University of Silesia, 9th Szkolna Street, 40-006 Katowice, Poland; E-mail:barbara.machura@us.edu.pl*

[b]*Department of Physical Chemistry and Technology of Polymers, Silesian University of Technology, Strzody 9, 44-100 Gliwice, Poland*

[c] *Centre of Polymer and Carbon Materials, Polish Academy of Sciences, 34 M. Curie-Sklodowska Str., 41-819 Zabrze, Poland*

[d] *Nanophotonics Group, Institute of Physics, Faculty of Physics, Astronomy and Informatics, Nicolaus Copernicus University, 5 Grudziadzka Str., 87-100 Torun, Poland*

[e] *Department of Chemical Organic Technology and Petrochemistry, Silesian University of Technology, Krzywoustego 4, 44-100 Gliwice, Poland*

## Table of Contents


| **NMR spectra** | |
|---|---|
| 1. NMR spectra of **1** in DMSO-d$_6$: $^1$H, $^{13}$C and 2D | Figure S1 |
| 2. $^1$H NMR spectrum of **2** in DMSO-d$_6$ | Figure S2 |
| **IR spectra** | |
| 3. FT-IR spectrum of **1** along with FT-IR spectrum of 9-anthryl-terpy. | Figure S3 |
| 4. FT-IR spectrum of **2** along with FT-IR spectrum of 2-anthryl-terpy. | Figure S4 |
| **HR-MS spectrometry** | |
| 5. HR-MS spectra of **1** and **2** | Figures S5–S6 |
| **Stability and Photostability** | |
| 6. UV-Vis spectra **1** and **2** in $CHCl_3$, MeCN and DMSO recorded once every two hours over 26 h at room temperature | Figure S7 |
| 7. UV-Vis spectra **1** and **2** in $CHCl_3$, MeCN and DMSO recorded after their exposure to light (450 nm) | Figure S8 |

| | |
|---|---|
| **UV-Vis** | |
| 8. Absorption maxima and molar extinction coefficients for **1** and **2** in solvents of different polarity ($CHCl_3$, $CH_3CN$ and DMSO), and as thin film on glass substrate | Table S1 |
| 9. UV-Vis absorption spectra of **1** and **2** in comparison to electronic spectra of the free ligands | Figure S9 |
| **DFT calculations** | |
| 10. Theoretical bond lengths [Å] and angles [°] for **1** and **2** | Table S2 |
| 11. Selected molecular orbitals of complexes **1** and **2** along with their energies [eV] and percentage compositions | Figure S10 |
| 12. The partial molecular orbital energy level diagrams for **1** and **2** in comparison to [ReCl(CO)$_3$(4′-Ar-terpy-κ$^2$N)] complex with the structural isomer 4′-(phenanthren-9-yl)-2,2′:6′,2″-terpyridine | Figure S11 |
| 13. Calculated ionization potentials and electron affinities (vertical and adiabatic), energy gaps (DFT/PBE1PBE/DEF2-TZVPD/DEF2-TZVP) of **1**and **2**. | Table S3 |
| **TD-DFT calculations** | |
| 14. The calculated transitions along with the experimental spectra, and natural transition orbitals for the lowest energy band of **1** and **2** | Figure S12 |
| 15. The energies and characters of the spin-allowed electronic transitions assigned to the lowest wavelength absorption bands of **1** to **2** computed at TD-DFT/PBE0/def2-TZVPD/def2-TZVP level with the use of PCM model at polarities corresponding to $CH_3CN$ | Table S4 |
| **Luminescence studies** | |
| 16. Emission spectral data of **1** and **2** in three solvents of different polarity ($CHCl_3$, $CH_3CN$ and DMSO), and rigid matrix at 77 K (BuCN) | Figures S13-14 |
| 17. Normalized emission spectra of **1** and **2** in $CH_3CN$, along with the emission spectra of [ReCl(CO)$_3$(4′-Ar-terpy-κ$^2$N)] incorporating 2,2′:6′,2″-terpyridines functionalized with 1-naphtyl, 2-naphtyl and 9-phenanthryl groups | Figure S15 |
| 18. Normalized emission spectra of **1** and **2** in $CHCl_3$, along with the emission of free ligands in $CHCl_3$ | Figure S16 |
| 19. Normalized emission spectra of **1** and **2** in rigid matrix at 77 K, with the emission spectra of [ReCl(CO)$_3$(4′-Ar-terpy-κ$^2$N)] incorporating 2,2′:6′,2″-terpyridines functionalized with 1-naphtyl, 2-naphtyl and 9-phenanthryl groups | Figure S17 |
| 20. Phosphorescence spectra of **1** at 77 K along with the phosphorescence spectra of the free ligand and anthracene | Figure S18 |
| 21. Phosphorescence spectra of **2** at 77 K along with the phosphorescence spectra of the free ligand and anthracene | Figure S19 |
| 22. Photoluminescence properties of complexes **1** and **2** upon excitation 335 nm | Table S5 |
| **Phosphorescence emissions – computational data** | |
| 23. The energies of theoretical phosphorescence emissions, calculated from the difference between the ground singlet and the triplet state | Table S6 |

| | |
|---|---|
| $\Delta E_{T_1-S_0}$, along with the experimental values and the spin density surface plots for **1** and **2** | |
| **Femtosecond transient absorption spectroscopy** | |
| 24. Fluence dependence and photo-stability tests of **1** and **2** prior femtosecond transient absorption experiment. | Figure S20 |
| 25. Summary of fsTA measurements for complexes **1** and **2** excited at 355 nm: fsTA 2D maps (panel A), TA spectra at selected time delays (panel B), and decay associated spectra, DAS (panel C). | Figure S21 |
| 26. Summary of fsTA measurements for complexes **1** and **2** excited at 405 nm: fsTA 2D maps (panel A), TA spectra at selected time delays (panel B), and decay associated spectra, DAS (panel C) | Figure S22 |
| 27. 2D maps presenting time traces along with the fitting curves from the global analysis for complexes **1** and **2** excited at 355 nm | Figure S23 |
| 28. 2D maps presenting time traces along with the fitting curves from the global analysis for complexes **1** and **2** in $CHCl_3$ excited at 355 nm | Figure S24 |
| 29. Comparison of fsTA spectra at early time delay (0.2 – 0.5 ps) of **1** and parent chromophore [ReCl(CO)$_3$(terpy-κ$^2$N)] | Figure S25 |
| **$^1O_2$ generation** | |
| 30. UV-vis absorption spectra of diphenylisobenzofuran (DPBF) in DMSO (c – 50 μM) treated with complexes **1** and **2** (c – 50 and 25 μM, respectively for **1** and **2**) upon exposure to visible light at 420 nm recorded over 240 s. | Figure S26 |
| 31. UV-vis absorption spectra of diphenylisobenzofuran (DPBF) in DMSO (c – 50 μM) treated with free ligands and anthracene (c – 50 and 25 μM, respectively for 1 and 2) upon exposure to visible light at 380 nm recorded over 240 s. | Figure S27 |
| 32. Relative changes in absorbance of DPBF at 417 nm (A/A$_0$) with time | Figure 28 |

# NMR spectra

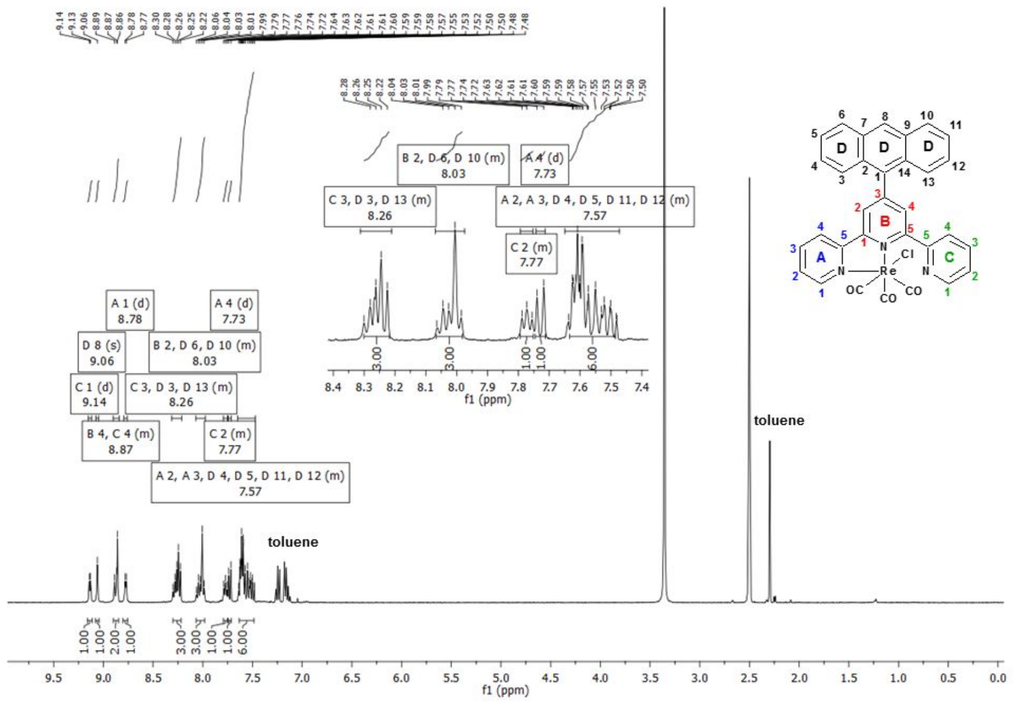

**(a)**

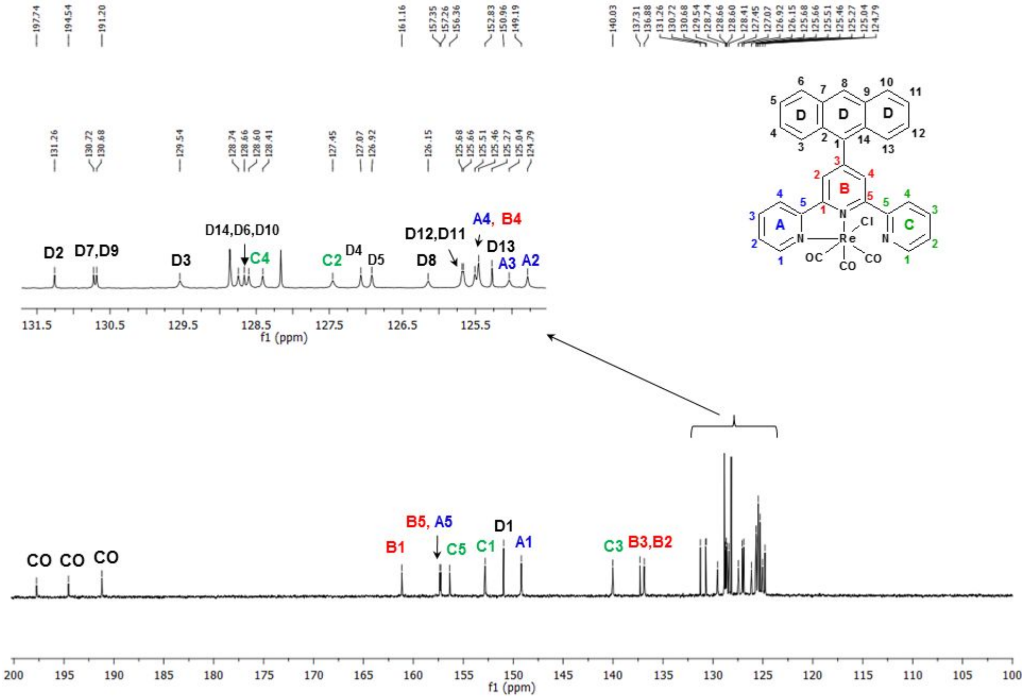

**(b)**

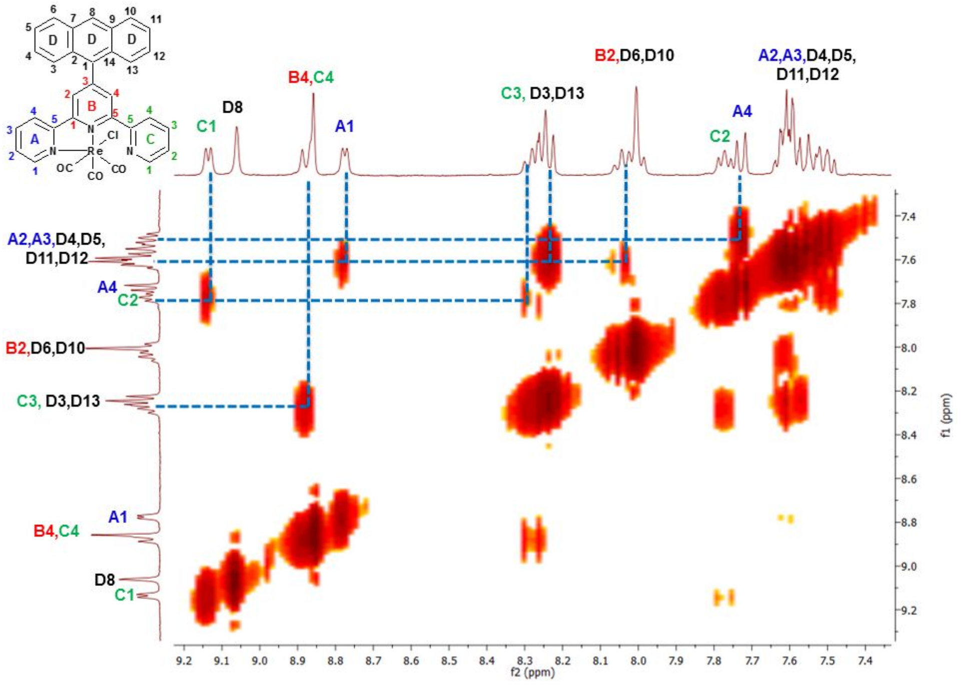

A2,A3,D4,D5,
D11,D12
A4
C2
B2,D6,D10
C3, D3,D13
A1
B4,C4
D8
C1
f2 (ppm)
f1 (ppm)


**(c)**

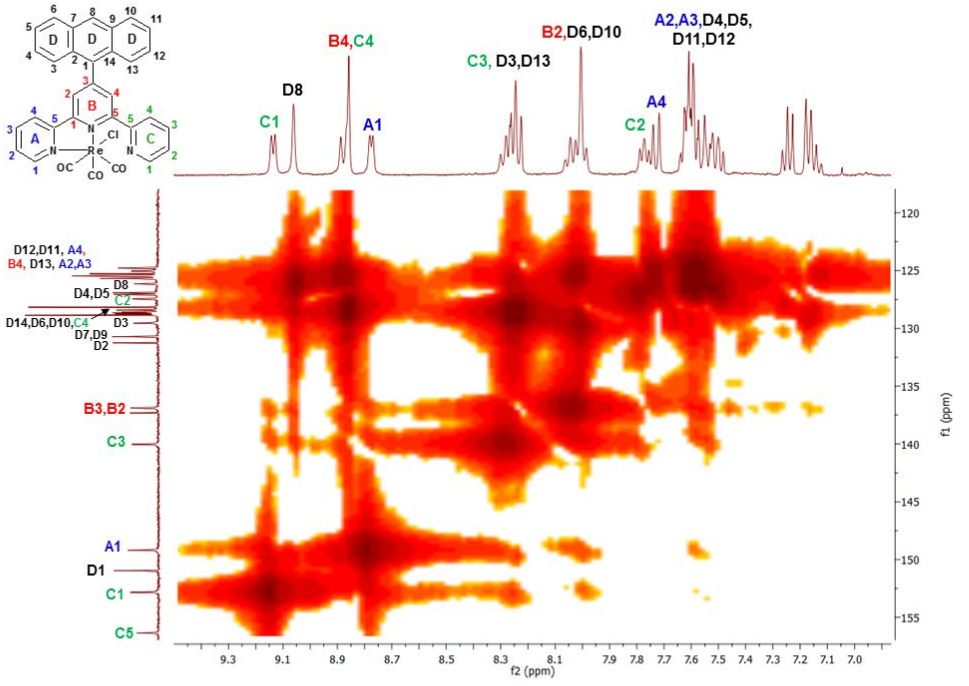

C1
D8
B4,C4
A1
C3, D3,D13
B2,D6,D10
C2
A4
A2,A3,D4,D5,
D11,D12
D12,D11, A4,
B4, D13, A2,A3
D8
D4,D5
C2
D14,D6,D10,C4
D3
D7,D9
D2
B3,B2
C3
A1
D1
C1
C5
f2 (ppm)
f1 (ppm)


**(d)**

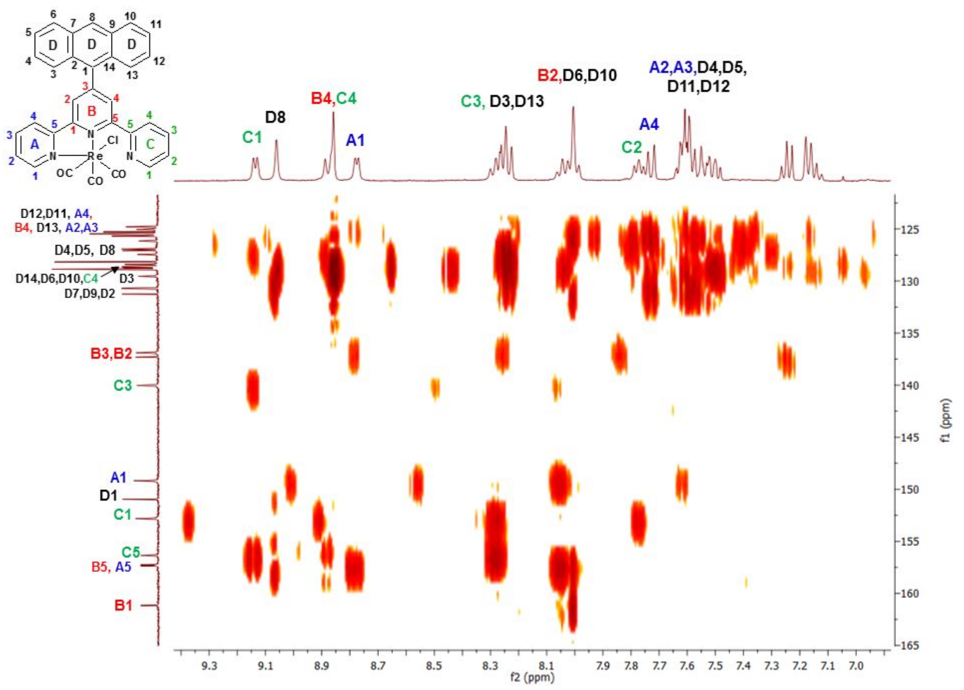

**(e)**

**Figure S1.** NMR spectra of **1** in DMSO-d$_6$: $^1$H (a), $^{13}$C (b), $^1$H–$^1$H COSY (c), $^1$H–$^{13}$C HMQC (d), $^1$H–$^{13}$C HMBC (e).

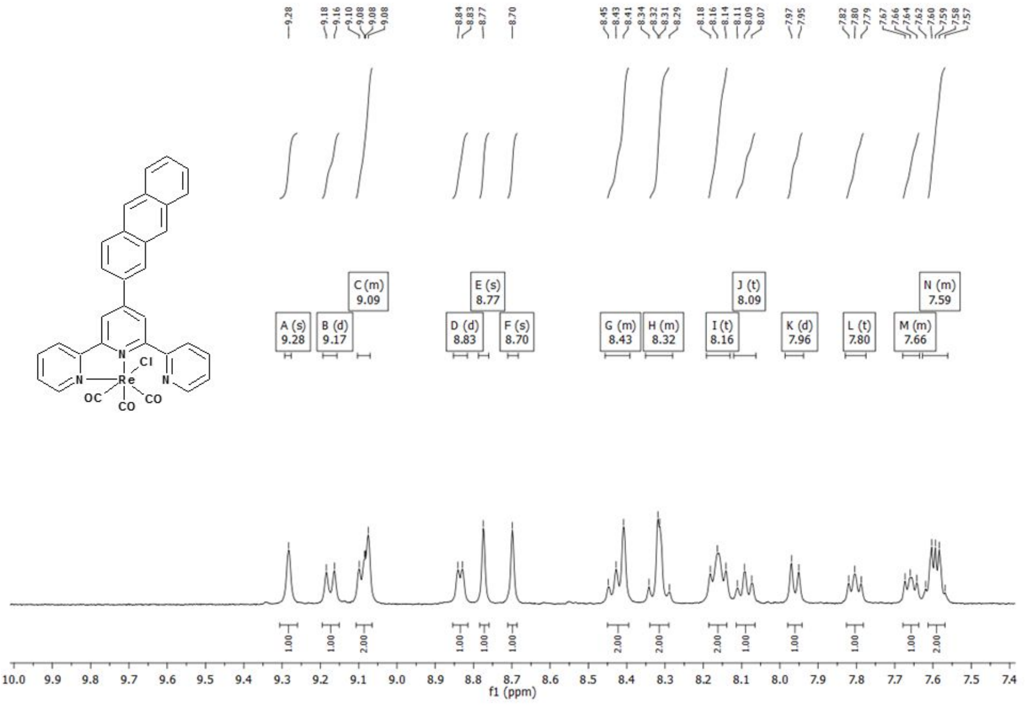

**Figure S2**. $^1$H NMR spectrum of **2** in DMSO-d$_6$. $^{13}$C NMR spectrum does not recorded due to insufficient complex solubility.

# IR spectra

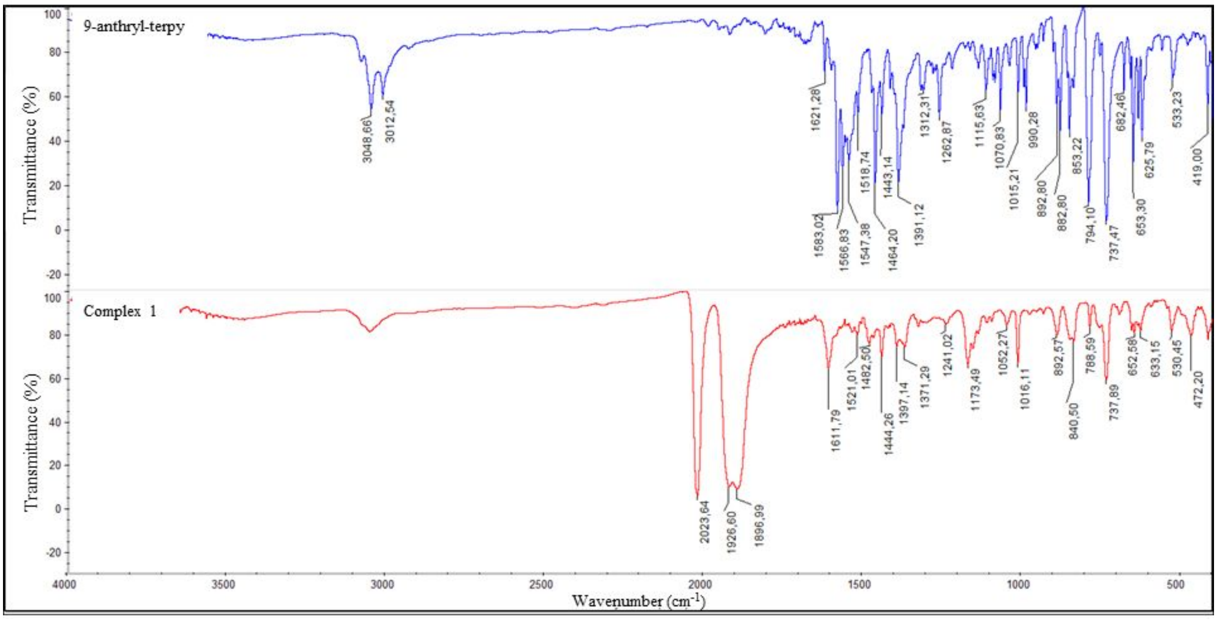


**Figure S3.** FT-IR spectrum of **1** along with FT-IR spectrum of **9-anthryl-terpy**.

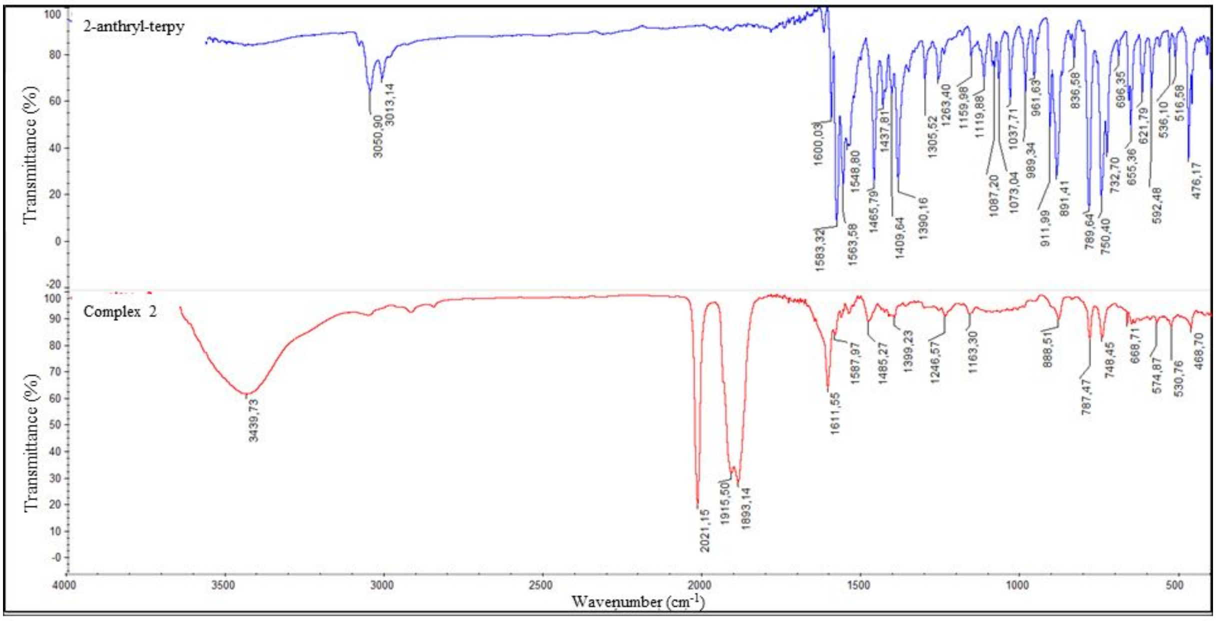


**Figure S4.** FT-IR spectrum of **2** along with FT-IR spectrum of **2-anthryl-terpy**.

# HR-MS spectrometry

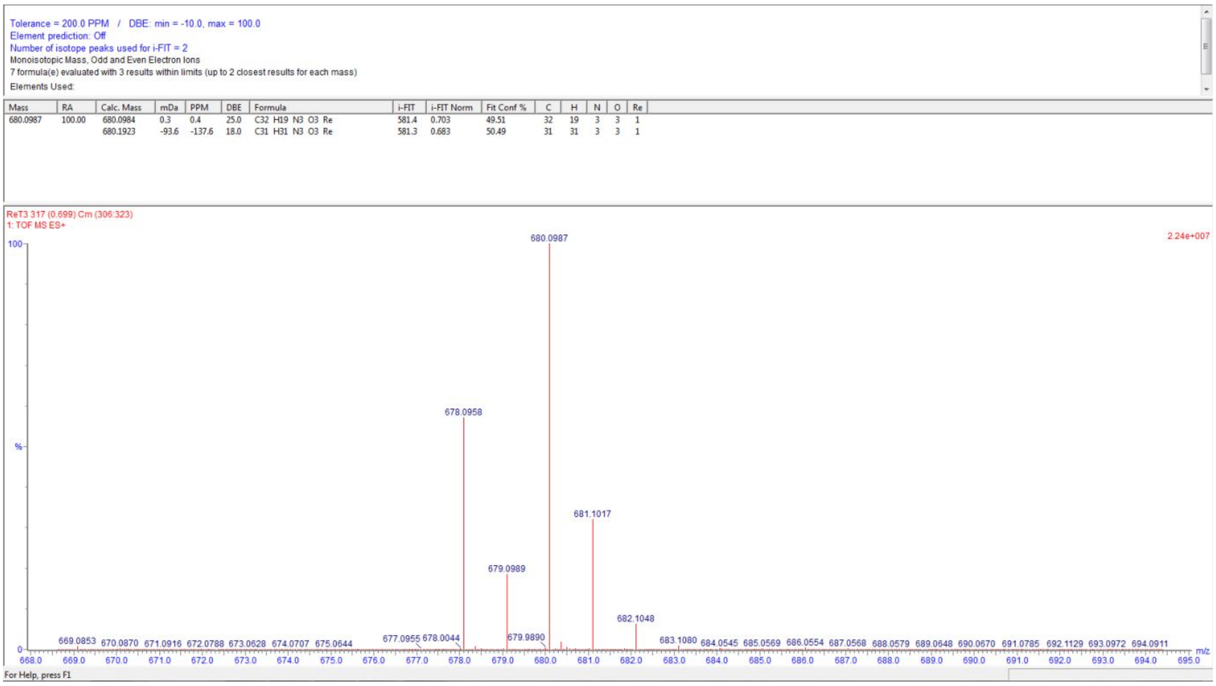


**Figure S5.** HRMS spectrum of **1**.

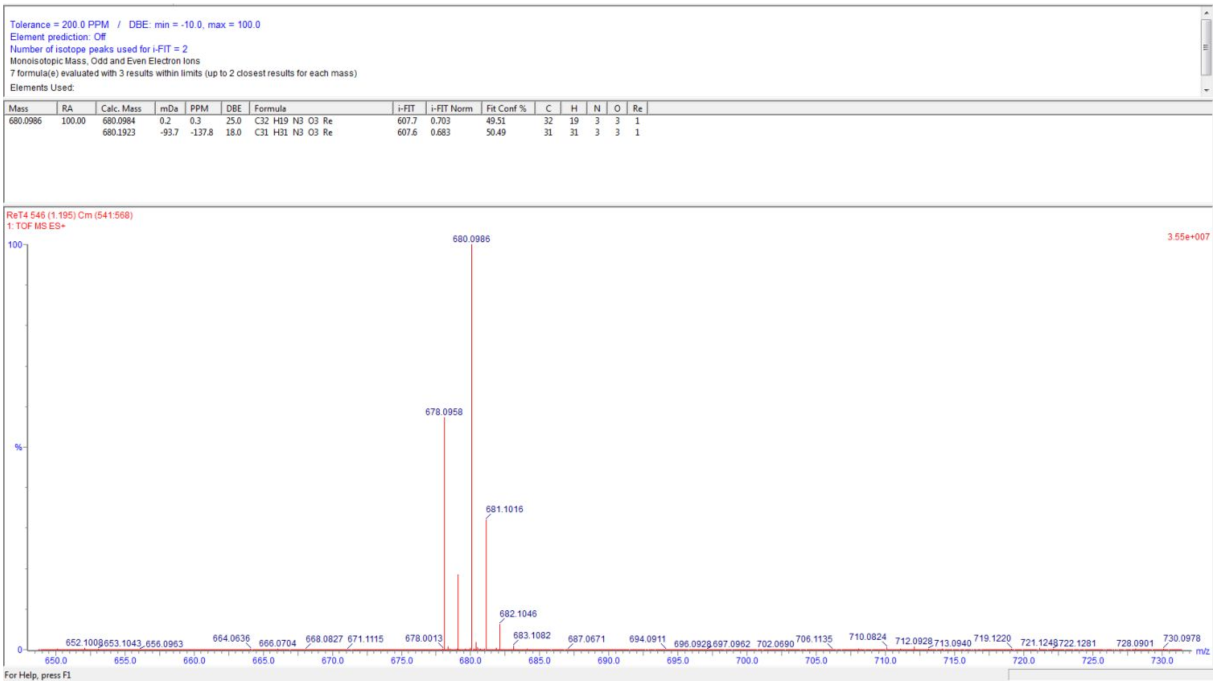


**Figure S6.** HRMS spectrum of **2**.

# Stability and Photostability

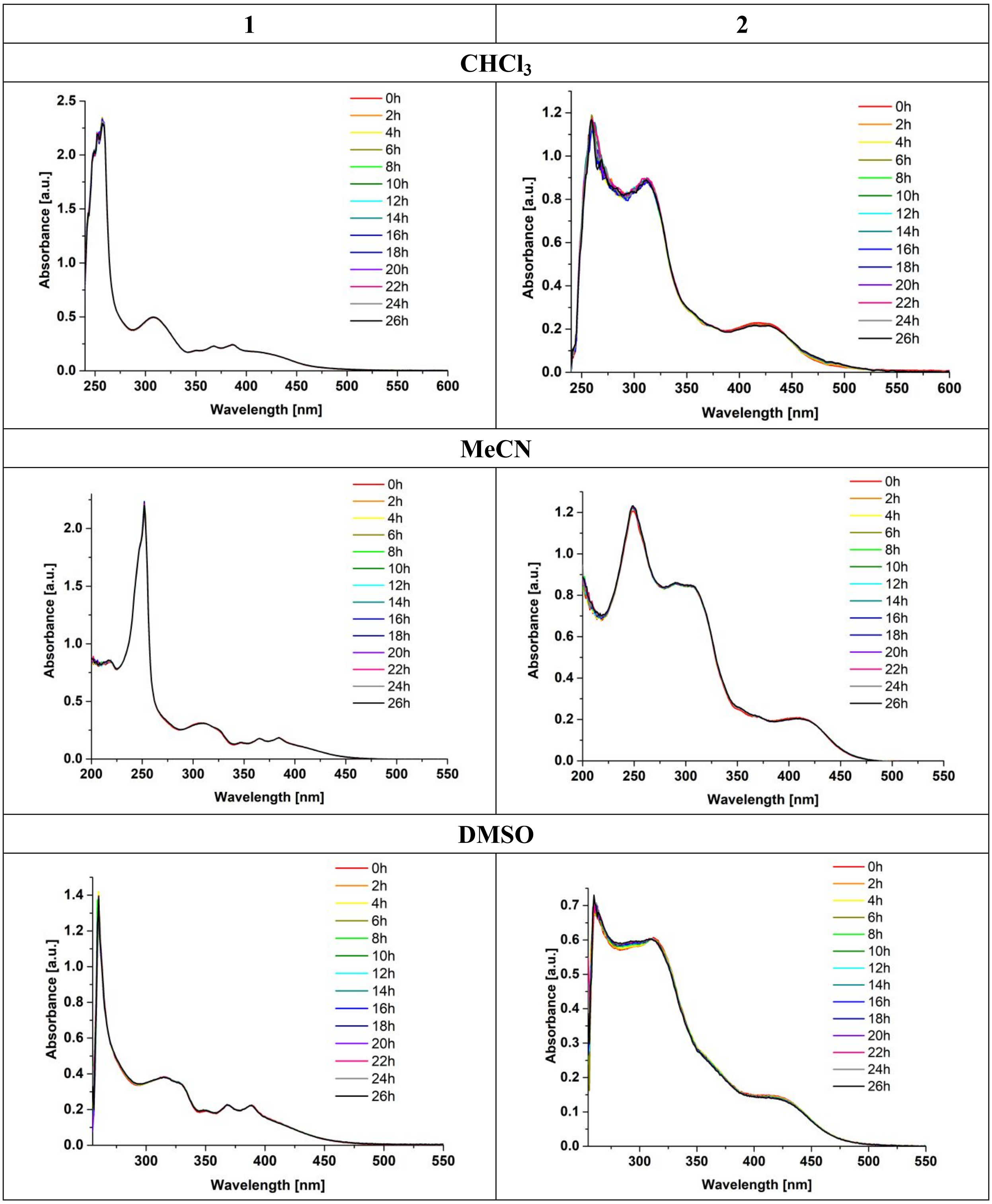


**Figure S7.** UV-Vis spectra **1** and **2** in $CHCl_3$, MeCN and DMSO recorded once every two hours over 26 h at room temperature.

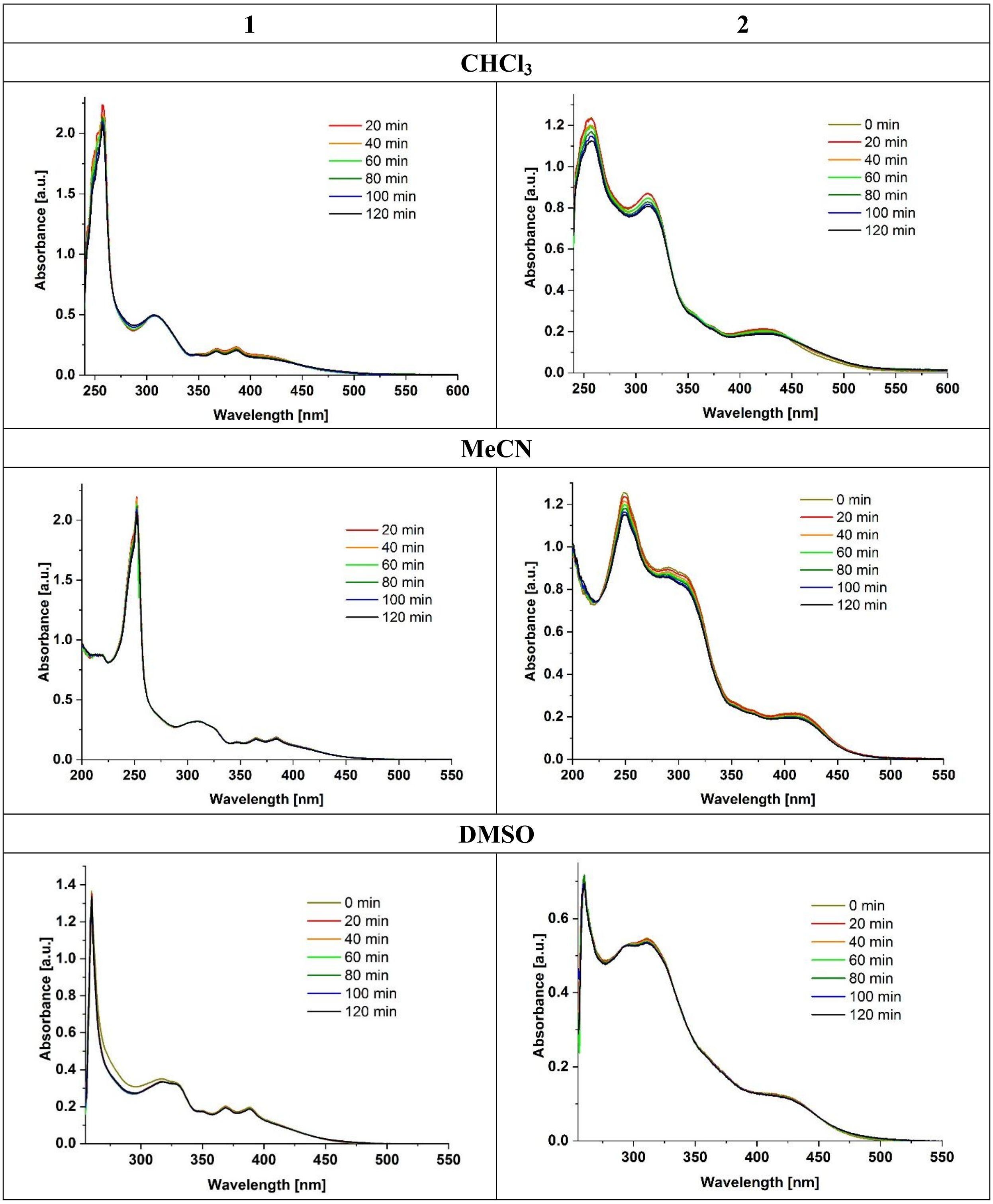


**Figure S8.** UV-Vis spectra **1** and **2** in $CHCl_3$, MeCN and DMSO recorded after their exposure to light (450 nm).

# UV-Vis

**Table S1.** Absorption maxima and molar extinction coefficients for **1** and **2** in solvents of different polarity ($CHCl_3$, MeCN and DMSO), and as thin films on glass substrate.

| Compound | Medium | λ (nm) ( ε ($M^{-1}cm^{-1}$)) |
|---|---|---|
| **1** | $CHCl_3$ | 416 (6672), 386 (9730), 368 (9051), 351 (7692), 308 (19770), 258 (92485) |
| | MeCN | 407 (4100), 384 (7386), 365 (6936), 348 (5616), 311 (12693), 252 (88008) |
| | DMSO | 406 (5670), 388 (8980), 368 (9062), 349 (7583), 328 (13956), 316 (15289), 260 (53314) |
| | film | 456, 392, 370, 321sh |
| **2** | $CHCl_3$ | 423 (9141), 312 (35496), 259 (45577) |
| | MeCN | 412 (8356), 305 (33778), 292 (24334), 250 (48204) |
| | DMSO | 421 (5836), 360 (10019), 312 (24285), 262 (27508) |
| | film | 450, 326sh |

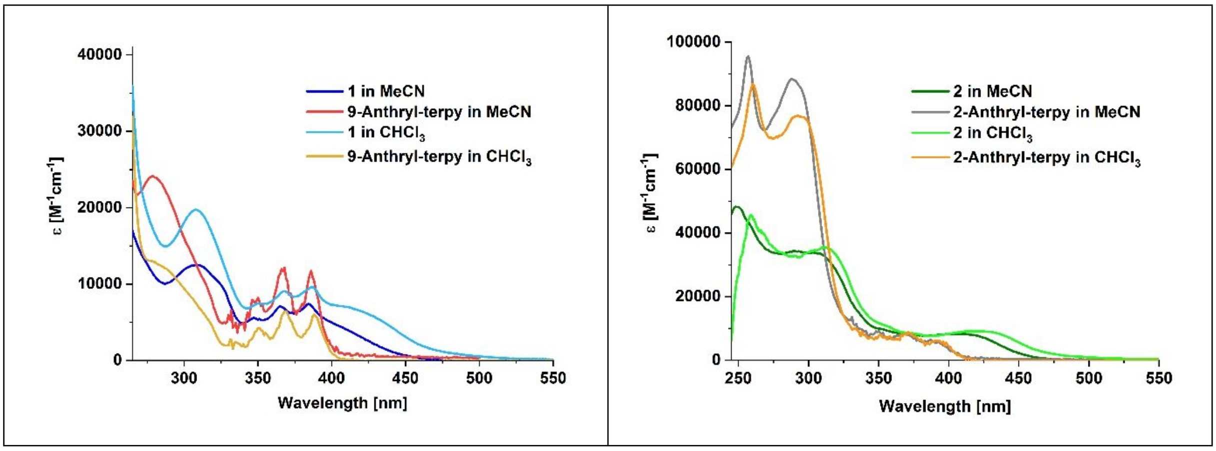


**Figure S9.** UV-Vis absorption spectra of **1** and **2** in comparison to electronic spectra of the free ligands.

# DFT calculations

**Table S2.** Theoretical bond lengths [Å] and angles [°] for **1** and **2**.

| | **1** | **2** |
|---|---|---|
| | 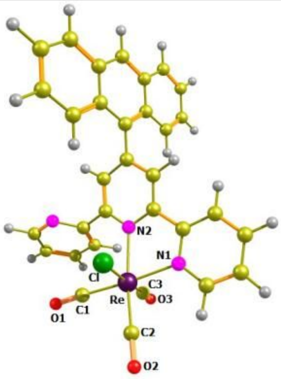  | 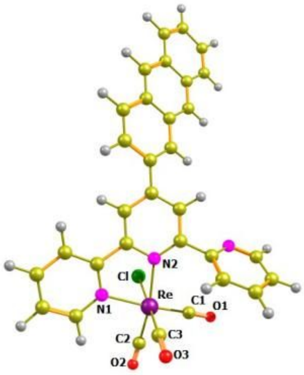  |
| Bond lengths | | |
| Re(1)–C(1) | 1.923 | 1.923 |
| Re(1)–C(2) | 1.906 | 1.906 |
| Re(1)–C(3) | 1.901 | 1.901 |
| Re(1)–N(1) | 2.178 | 2.179 |
| Re(1)–N(2) | 2.241 | 2.238 |
| Re(1)–Cl(1) | 2.499 | 2.500 |
| C(1)–O(1) | 1.149 | 1.149 |
| C(2)–O(2) | 1.152 | 1.152 |
| C(3)–O(3) | 1.155 | 1.155 |
| Bond angles | | |
| C(2)–Re(1)–C(1) | 85.80 | 85.88 |
| C(3)–Re(1)–C(1) | 89.94 | 89.94 |
| C(3)–Re(1)–C(2) | 88.49 | 88.52 |
| C(1)–Re(1)–N(1) | 175.00 | 175.03 |
| C(2)–Re(1)–N(1) | 97.33 | 97.33 |
| C(3)–Re(1)–N(1) | 94.04 | 93.92 |
| C(1)–Re(1)–N(2) | 102.18 | 102.10 |
| C(2)–Re(1)–N(2) | 170.12 | 170.09 |
| C(3)–Re(1)–N(2) | 97.25 | 97.29 |
| N(1)–Re(1)–N(2) | 74.33 | 74.33 |
| C(1)–Re(1)–Cl(1) | 91.76 | 91.83 |
| C(2)–Re(1)–Cl(1) | 92.30 | 92.18 |
| C(3)–Re(1)–Cl(1) | 178.18 | 178.14 |
| N(1)–Re(1)–Cl(1) | 84.23 | 84.28 |
| N(2)–Re(1)–Cl(1) | 81.74 | 81.79 |

| | 1 | 2 | Composition |
|---|---|---|---|
| **H-4** | 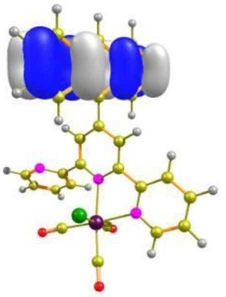 **-7.28 eV** | 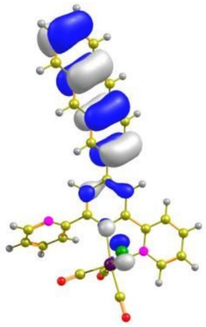 **-7.22 eV** | 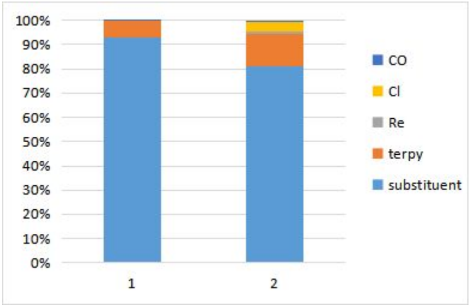  |
| **H-3** | 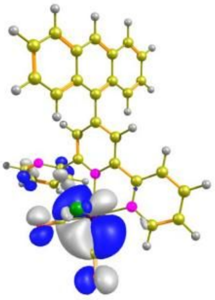 **-6.95 eV** | 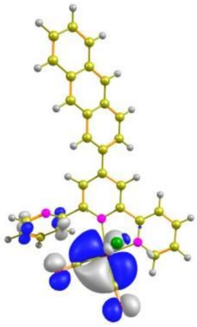 **-6.94 eV** | 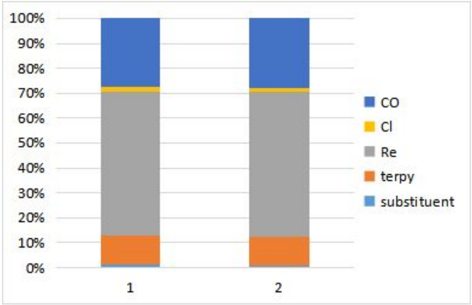  |
| **H-2** | 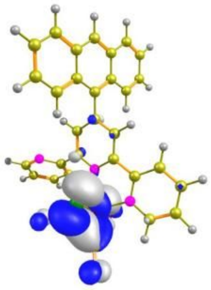 **-6.60 eV** | 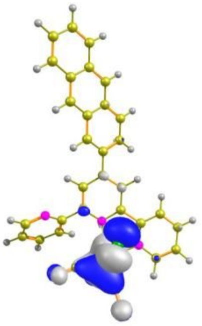 **-6.57 eV** | 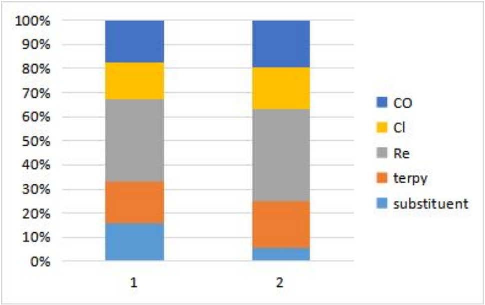  |
| **H-1** | 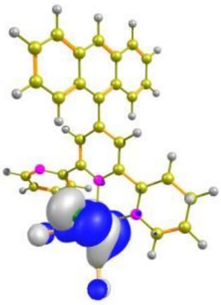 **-6.46 eV** | 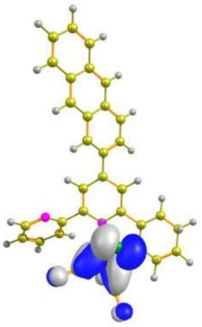 **-6.45 eV** | 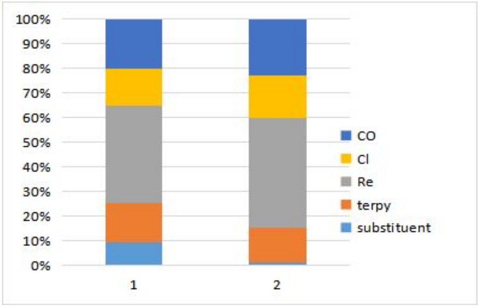  |

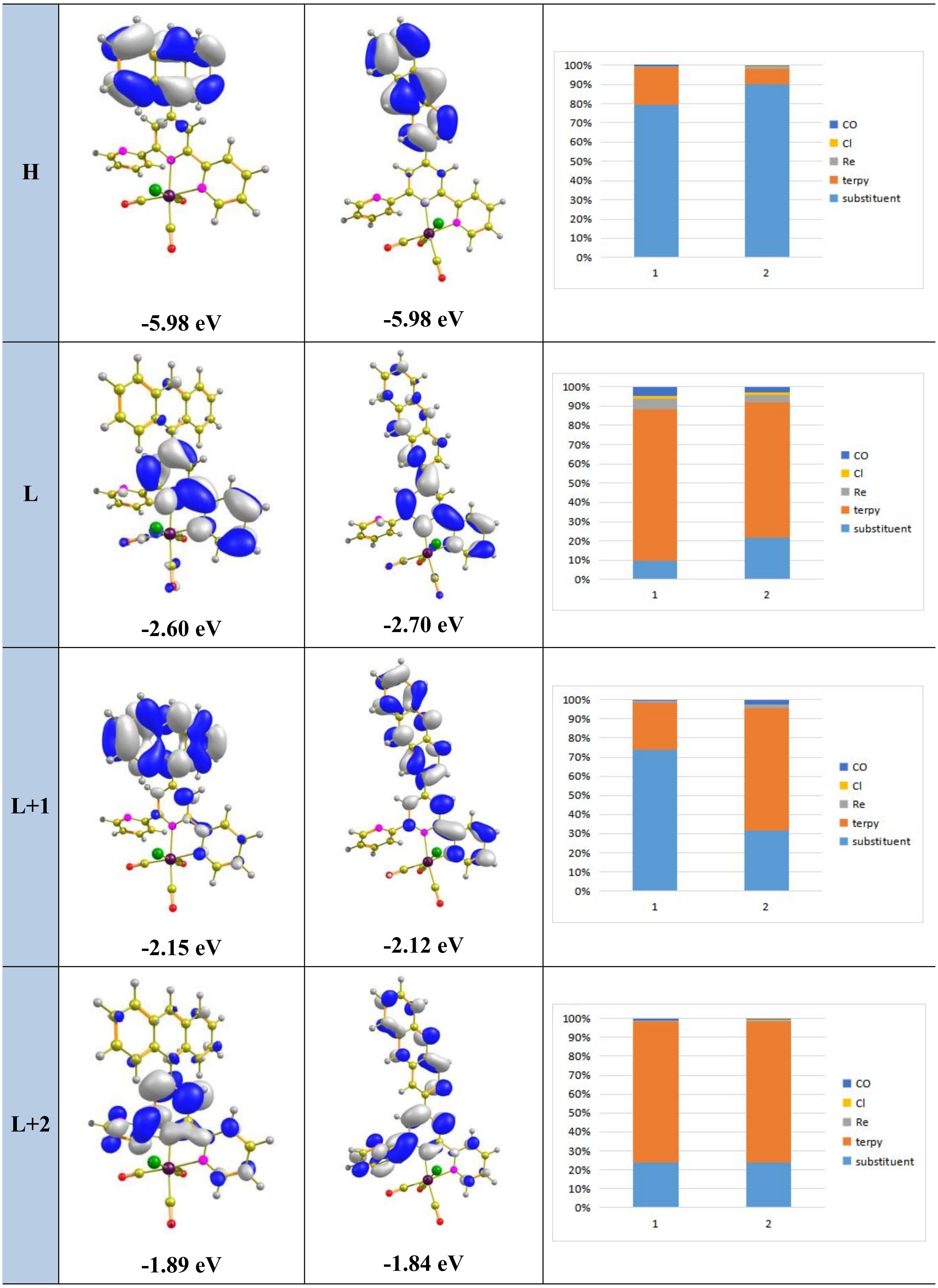


**Figure S10.** Selected molecular orbitals of complexes **1** and **2** along with their energies [eV] and percentage compositions.

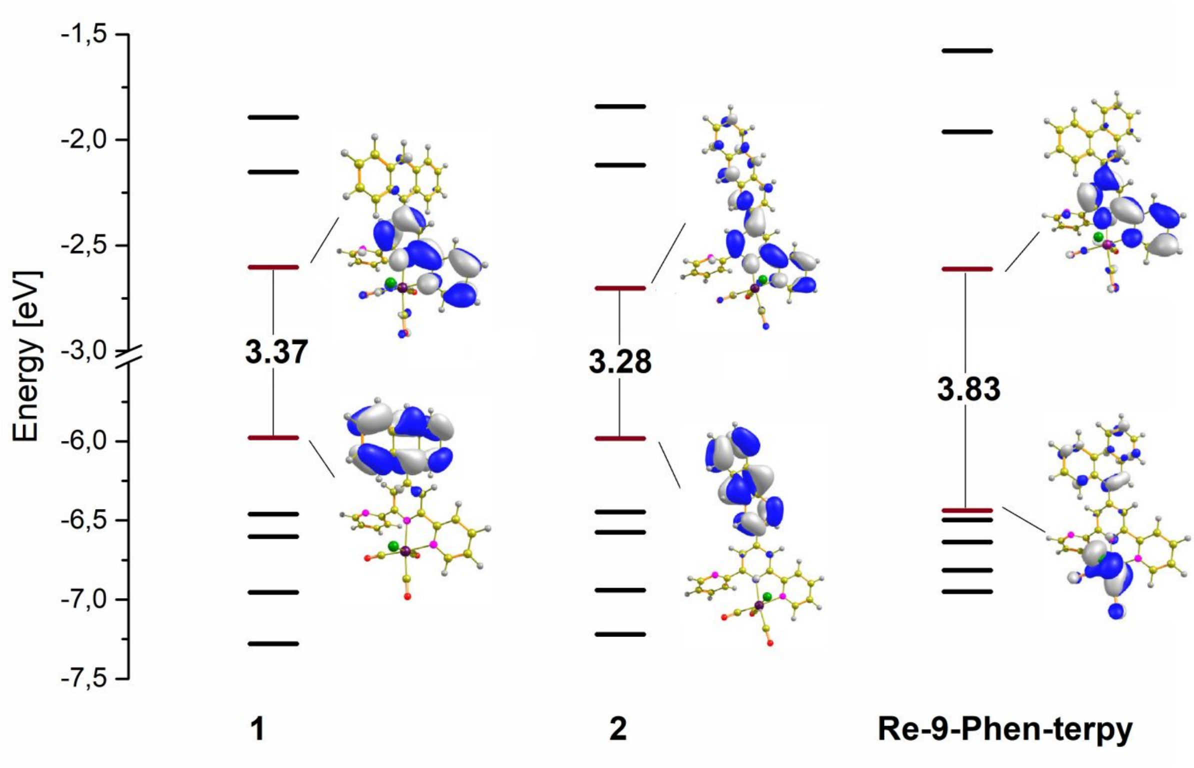


**Figure S11.** The partial molecular orbital energy level diagrams for **1** and **2** in comparison to [ReCl(CO)$_3$(4′-Ar-terpy-κ$^2$N)] complex with the structural isomer **4′-(phenanthren-9-yl)-2,2′:6′,2″-terpyridine** [ref. 30 in manuscript]. Copyright by license of Inorganic Chemistry (Creative Commons Attribution 4.0 International License).

**Table S3**. Calculated ionization potentials and electron affinities (vertical and adiabatic), energy gaps (DFT/PBE1PBE/DEF2-TZVPD/DEF2-TZVP) of **1**and **2**.

| **Complex** | **IP(v) [eV]** | **IP(a) [eV]** | **EA(v) [eV]** | **EA(a) [eV]** | **energy gap(a) [eV]** |
|---|---|---|---|---|---|
| **1** | 5.70 | 5.62 | 2.88 | 3.06 | 2.56 |
| **2** | 5.70 | 5.63 | 2.97 | 3.12 | 2.50 |

# TD-DFT calculations

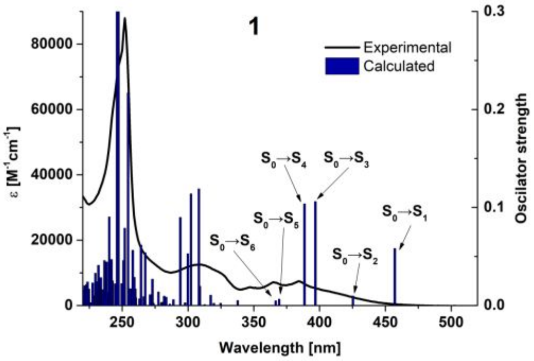


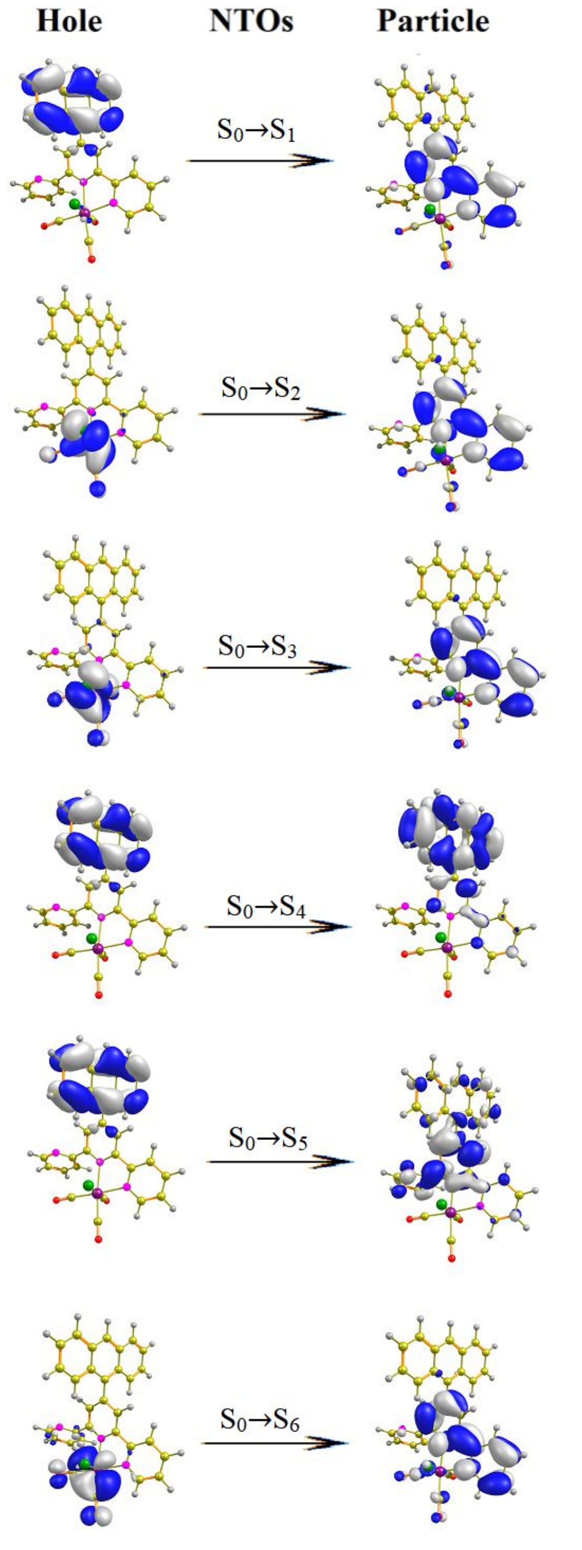

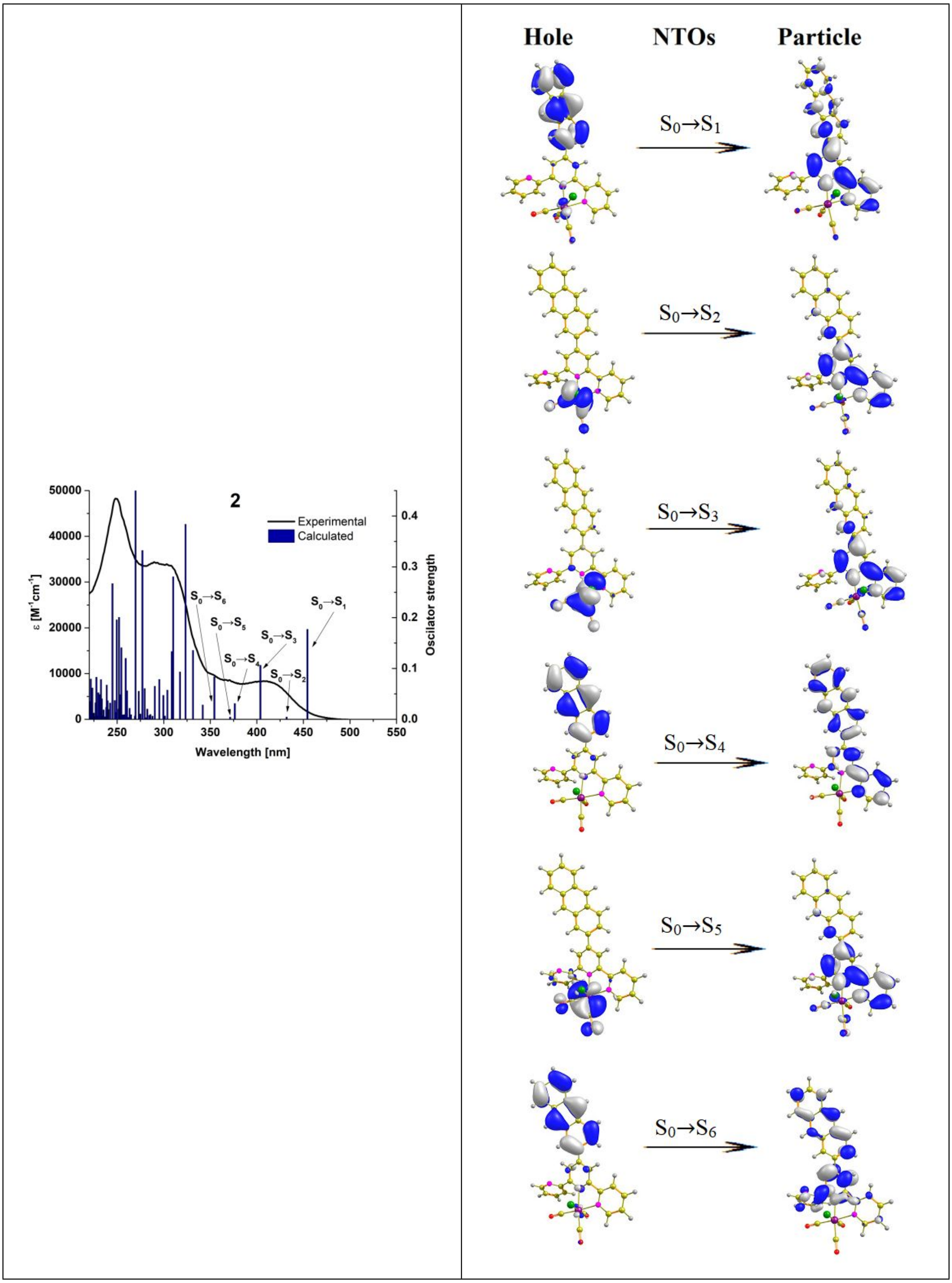


**Figure S12.** The calculated transitions along with the experimental spectra and natural transition orbitals for the lowest energy band of **1** and **2**.

**Table S4.** The energies and characters of the spin-allowed electronic transitions assigned to the lowest wavelength absorption bands of **1** to **2** computed at TD-DFT/PBE0/def2-TZVPD/def2-TZVP level with the use of PCM model at polarities corresponding to MeCN.

| Experimental absorption, λ [nm] ($10^4ε$ [$M^{-1}cm^{-1}$]) | Calculated transitions | | | | |
|---|---|---|---|---|---|
| | Excitation | Character | E[eV] | λ [nm] | Oscillator strength |
| **1** | | | | | |
| 407 (0.67) 384 (0.97) 365 (0.91) | $S_0 \rightarrow S_1$ | $\pi_{anthracene} \rightarrow \pi^*_{terpy}$ | 2.71 | 457.1 | *0.0582* |
| | $S_0 \rightarrow S_2$ | MLCT | 2.92 | 425.3 | *0.0098* |
| | $S_0 \rightarrow S_3$ | MLCT | 3.12 | 396.8 | *0.1061* |
| | $S_0 \rightarrow S_4$ | $\pi_{anthracene} \rightarrow \pi^*_{anthracene}/\pi^*_{terpy}$ | 3.19 | 388.5 | *0.1037* |
| | $S_0 \rightarrow S_5$ | $\pi_{anthracene} \rightarrow \pi^*_{anthracene}/\pi^*_{terpy}$ | 3.36 | 369.2 | *0.0066* |
| | $S_0 \rightarrow S_6$ | MLCT | 3.38 | 366.6 | *0.0048* |
| **2** | | | | | |
| 412 (0.84) | $S_0 \rightarrow S_1$ | $\pi_{anthracene} \rightarrow \pi^*_{anthracene}/\pi^*_{terpy}$ | 2.73 | 454.3 | *0.1772* |
| | $S_0 \rightarrow S_2$ | MLCT | 2.87 | 431.9 | *0.0046* |
| | $S_0 \rightarrow S_3$ | MLCT | 3.07 | 403.8 | *0.1063* |
| | $S_0 \rightarrow S_4$ | $\pi_{anthracene} \rightarrow \pi^*_{anthracene}/\pi^*_{terpy}$ | 3.29 | 376.3 | *0.031* |
| | $S_0 \rightarrow S_5$ | MLCT | 3.34 | 371.3 | *0.0046* |
| | $S_0 \rightarrow S_6$ | $\pi_{anthracene} \rightarrow \pi^*_{anthracene}/\pi^*_{terpy}$ | 3.50 | 354.3 | *0.0835* |

# Luminescence studies

<table>
<tr><th>Medium</th><th>Emission and Excitation</th><th>Lifetime</th></tr>
<tr><td colspan="3">1</td></tr>
<tr><td>MeCN</td><td>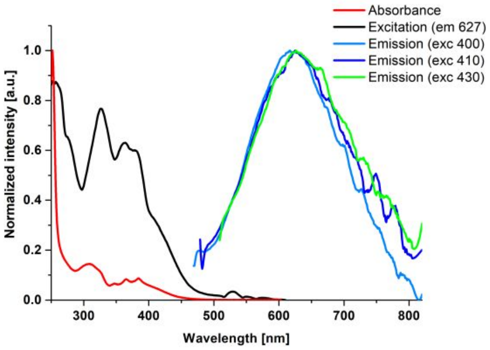
</td><td>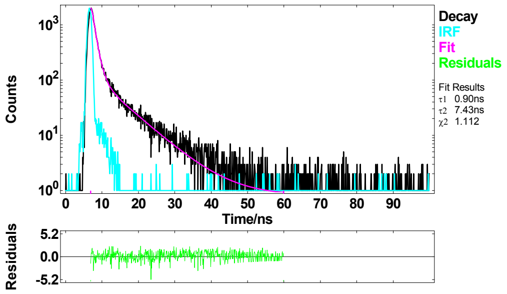

Fit results:<br>τ1 0.90 ns ±0.01 ns 79.08%<br>τ2 7.43 ns ±0.18 ns 20.92%</td></tr>
<tr><td>$CHCl_3$</td><td>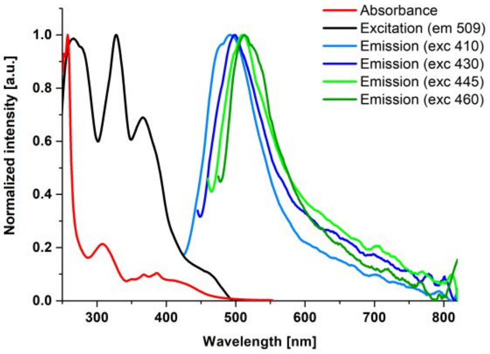
</td><td>For emission 511 nm<br>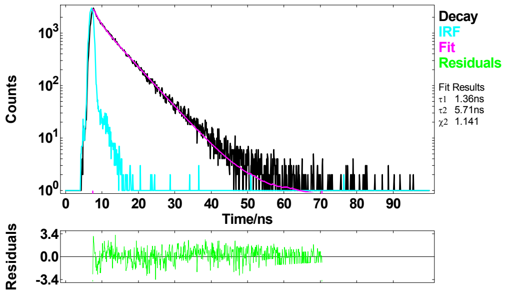

Fit results:<br>τ1 1.35ns ±0.07 ns 18.16%<br>τ2 5.71 ns ±0.14 ns 81.84%<br>For emission 650 nm<br>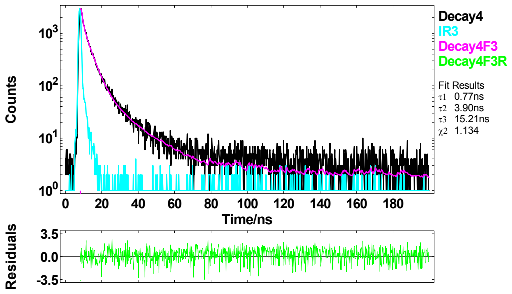

Fit results:<br>τ1 0.77 ns ±0.06 ns 26.94%<br>τ2 3.90 ns ±0.11 ns 58.66%<br>τ3 15.21 ns ±0.66 ns 14.41%</td></tr>
</table>

<table>
<tr>
<td>DMSO</td>
<td>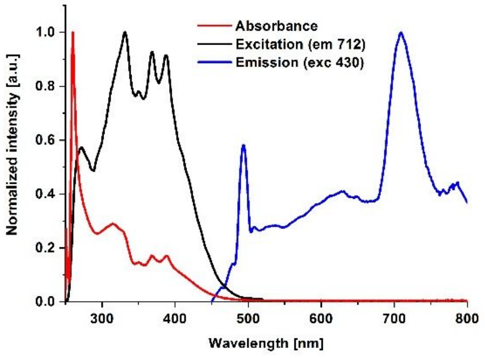
</td>
<td>For emission 627nm<br>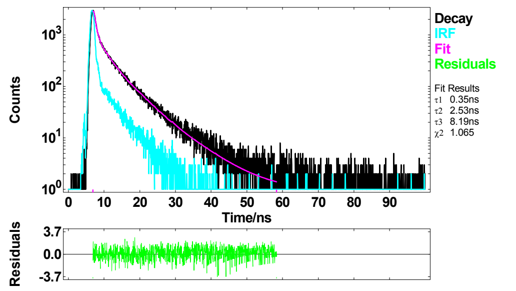
<br>Fit results:<br>τ1 0.35 ns ±0.02 ns 43.93%,<br>τ2 2.53 ns ±0.11 ns 33.83%,<br>τ3 8.19 ns ±0.29 ns 22.24%<br><br>For emission 712nm<br>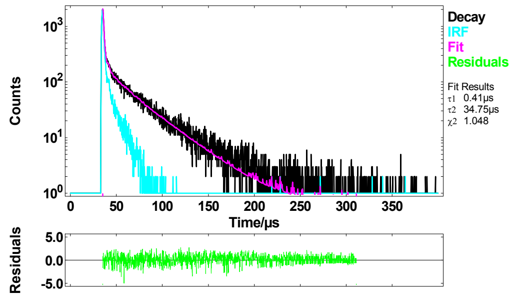
<br>Fit results:<br>τ1 0.41 μs ±0.03 μs 59.61%<br>τ2 34.75 μs ±0.40 ns 40.39%</td>
</tr>
<tr>
<td>BuCN<br>(77K)</td>
<td>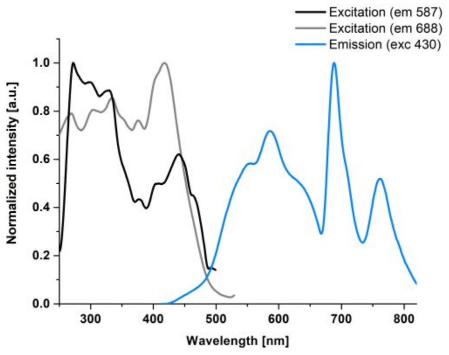
</td>
<td>For emission 587 nm<br>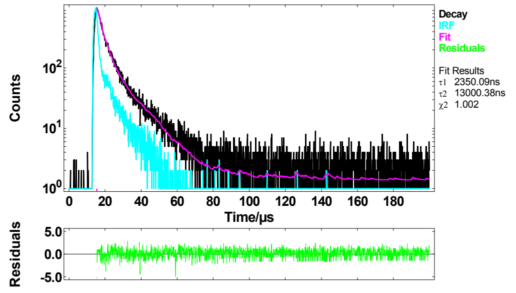
<br>Fit results:<br>τ2 2350.09 ns ±41.88 ns 88.92%<br>τ3 13000.38 ns ±710.20 ns 11.08%<br><br>For emission 688 nm</td>
</tr>
</table>

| | | |
|---|---|---|
| | | 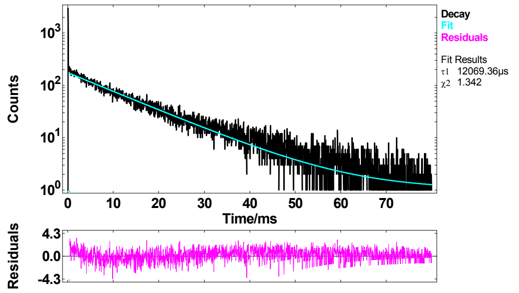<br>Fit results:<br>τ1 12069.36 μs ± 79.22 μs 100% |
| | **2** | |
| MeCN | 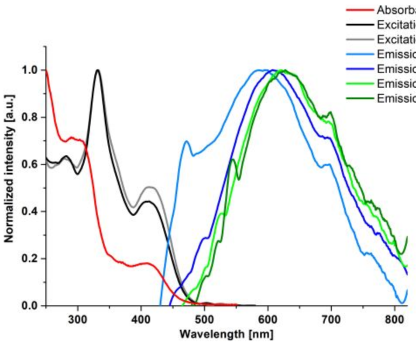 | 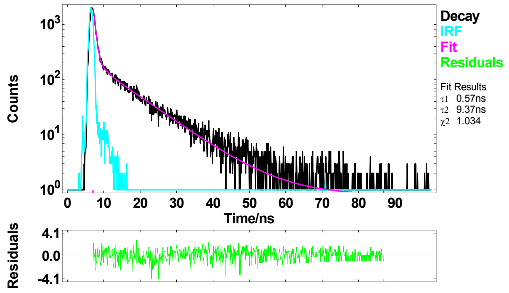<br>Fit results:<br>τ1 0.57 ns ±0.01 ns 61.35%<br>τ2 9.37 ns ±0.11 ns 38.65% |
| $CHCl_3$ | 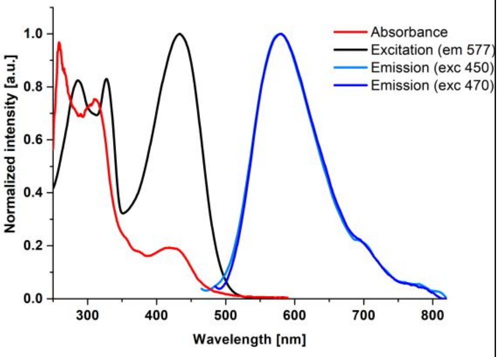 | 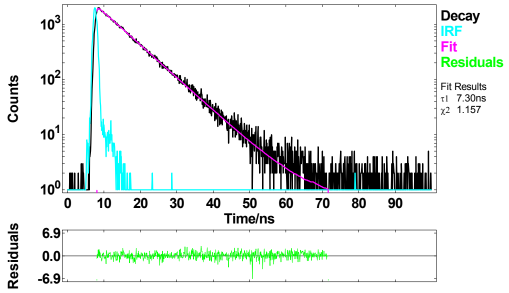<br>Fit results:<br>τ1 7.30 ns ±0.02 ns 100% |
| DMSO | 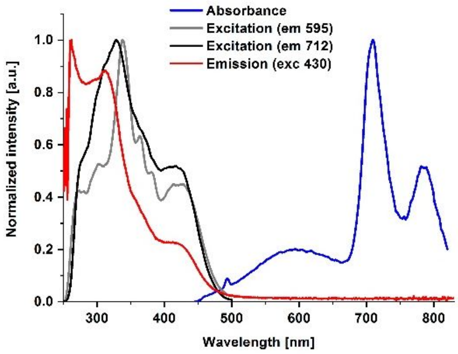 | **For emission 595nm**<br>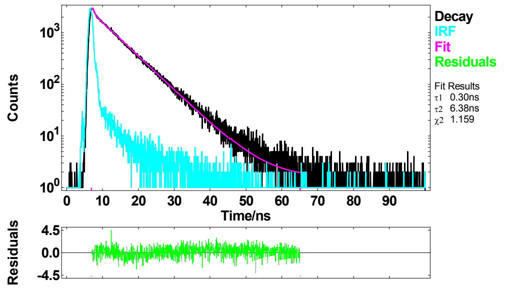<br>τ1 0.30 ns ±0.01 ns 10.40%<br>τ2 6.38 ns ±0.01 ns 89.60%<br><br>**For emission 712 nm** |

<table>
<tr>
<td></td>
<td></td>
<td>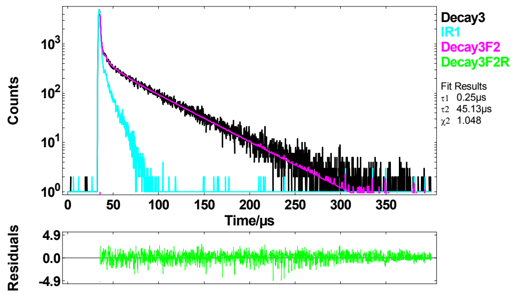

Fit results:<br>τ1 0.25 μs ±0.03 μs 49.96%<br>τ2 45.13 μs ±0.24 ns 50.04%</td>
</tr>
<tr>
<td>BuCN (77K)</td>
<td>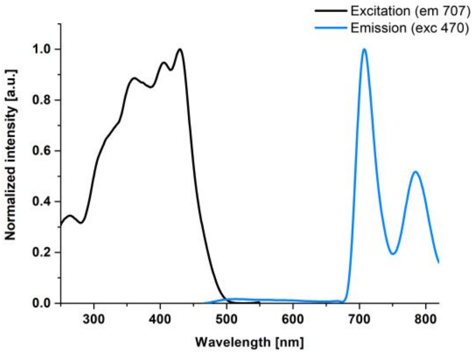
</td>
<td>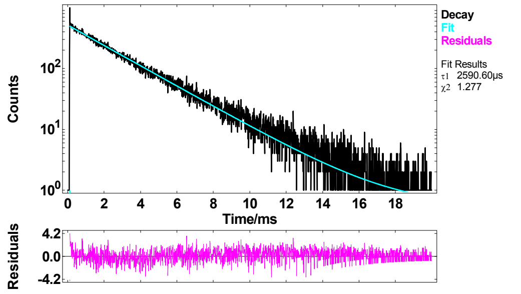

Fit results:<br>τ1 2590.60 μs ± 9.01 μs 100%</td>
</tr>
</table>

**Figure S13.** Emission spectral data of **1** and **2** in three solvents of different polarity ($CHCl_3$, ε = 4.81, η = 0.51 cP; MeCN, ε = 37.5, η = 0.34 cP and DMSO, ε = 46.7, η = 2.00 cP), and rigid matrix at 77 K (BuCN).

| 1 | 2 |
| --- | --- |
| $CHCl_3$ | |
| 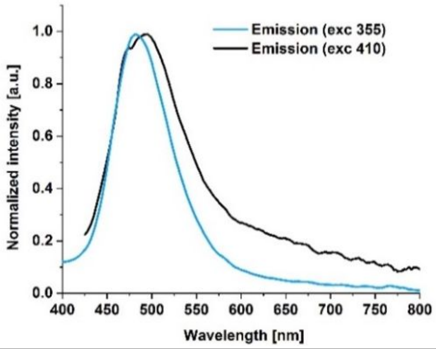 | 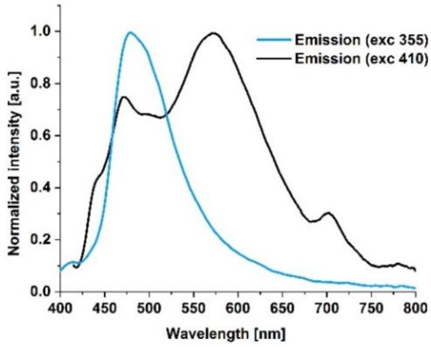 |
| MeCN | |
| 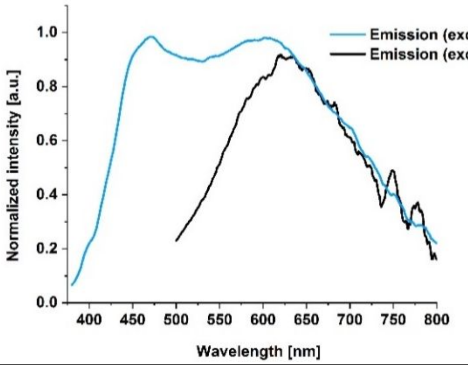 | 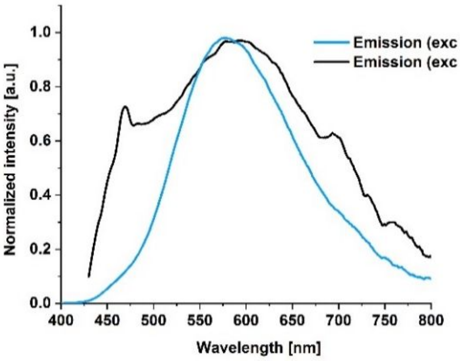 |
| DMSO | |
| 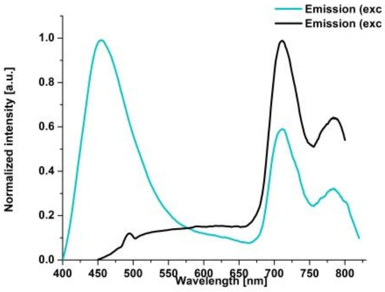 | 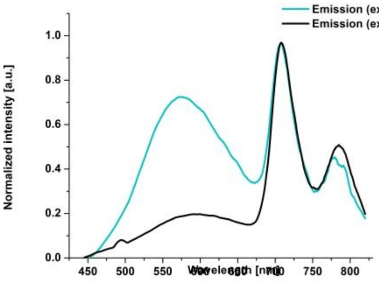 |
| 77K | |
| 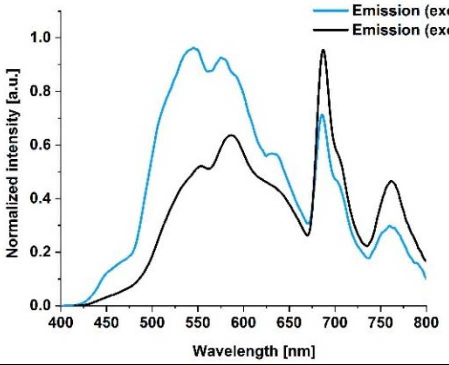 | 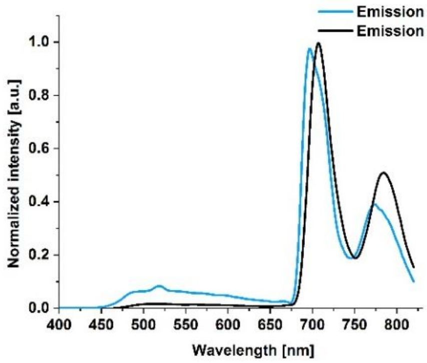 |

**Figure S14.** Comparison of emission spectra of **1** and **2** in three solvents of different polarity and at 77K upon excitations at $^1$An and $^1$MLCT absorption bands.

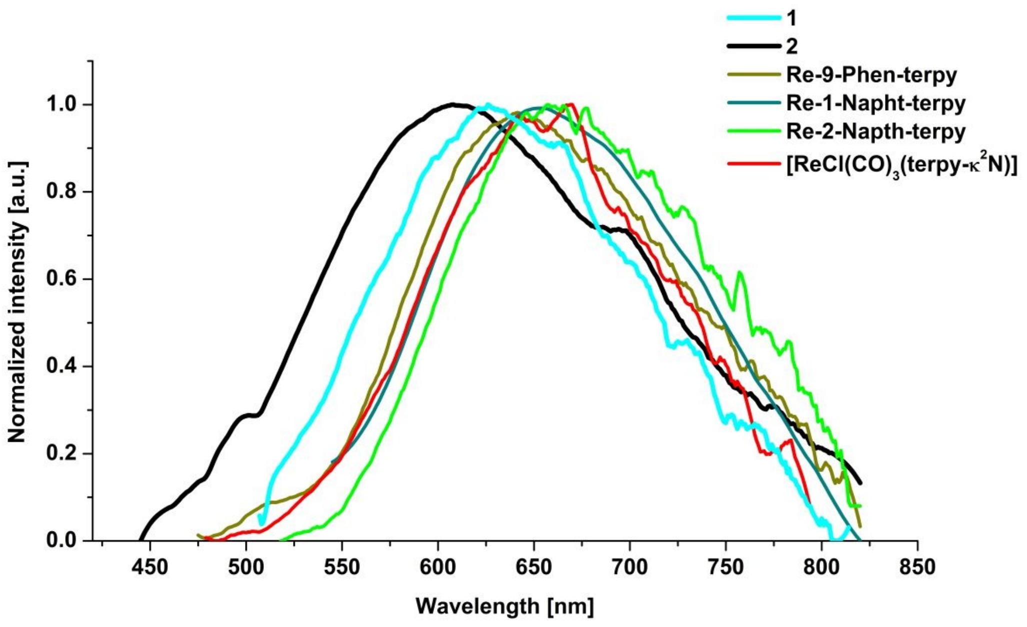


**Figure S15.** Normalized emission spectra of **1** and **2** in MeCN, along with the emission spectra of $[ReCl(CO)_3(terpy\text{-}\kappa^2N)]$ and $[ReCl(CO)_3(4'\text{-}Ar\text{-}terpy\text{-}\kappa^2N)]$ incorporating **2,2′:6′,2″-terpyridines functionalized with 1-naphtyl, 2-naphtyl and 9-phenanthryl groups** [ref. 30 in manuscript]. Copyright by license of Inorganic Chemistry (Creative Commons Attribution 4.0 International License).

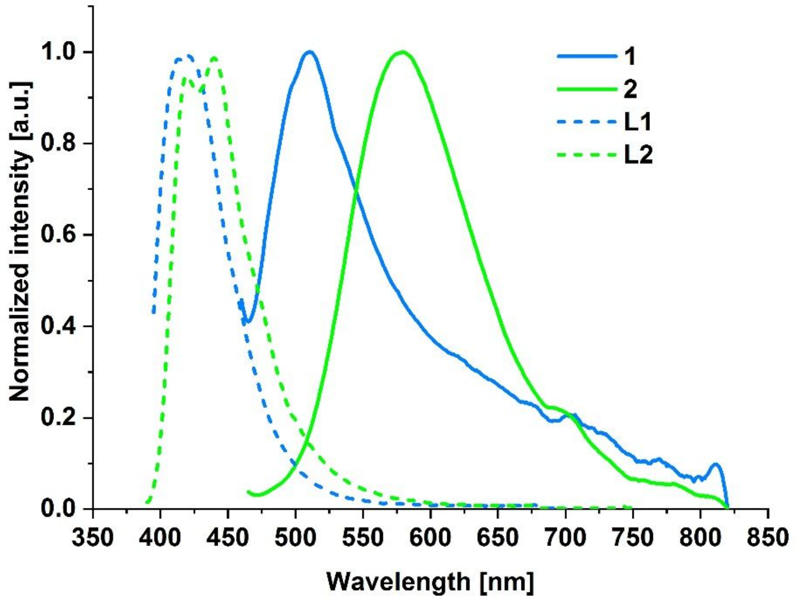


**Figure S16.** Normalized emission spectra of **1** and **2** in $CHCl_3$, along with the emission of free ligands in $CHCl_3$.

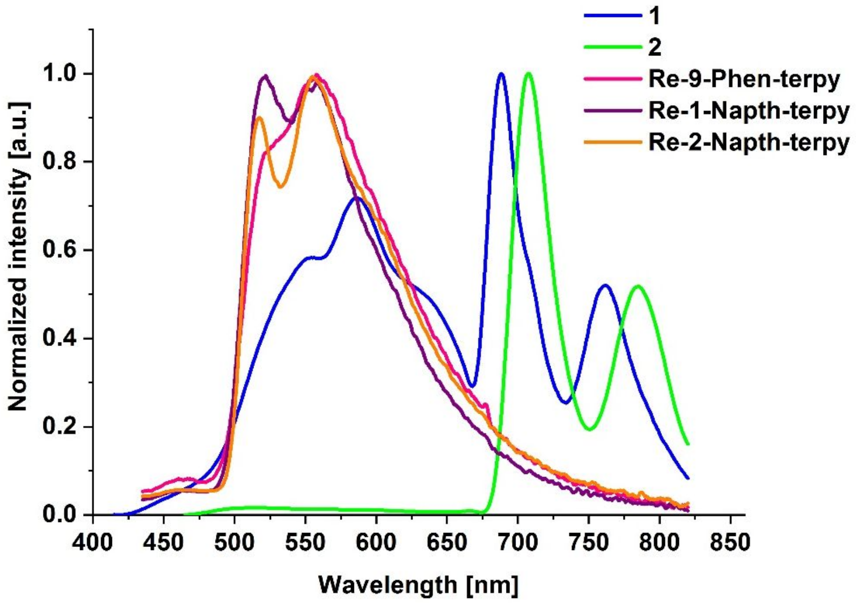


**Figure S17**. Normalized emission spectra of **1** and **2** in rigid matrix at 77 K, with the emission spectra of [ReCl(CO)$_3$(4′-Ar-terpy-κ$^2$N)] incorporating **2,2′:6′,2″-terpyridines functionalized with 1-naphtyl, 2-naphtyl and 9-phenanthryl groups** [ref. 30 in manuscript]. Copyright by license of Inorganic Chemistry (Creative Commons Attribution 4.0 International License)**.**

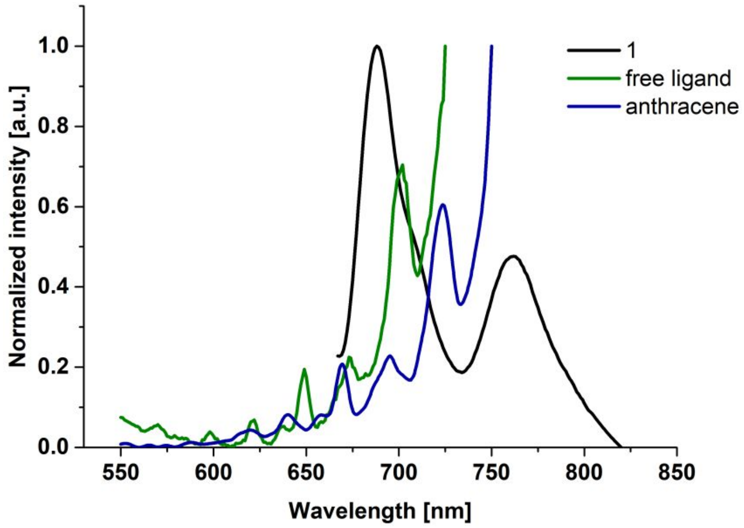


**Figure S18.** Phosphorescence spectra of **1** at 77 K along with the phosphorescence spectra of the free ligand and anthracene.

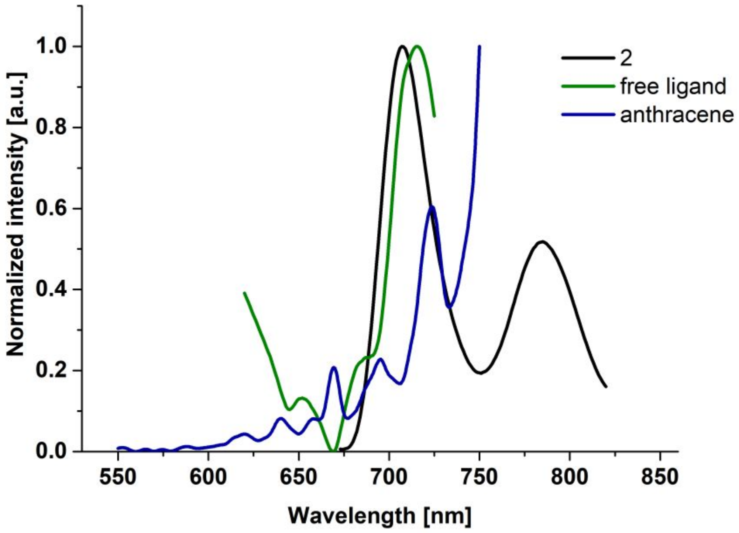


**Figure S19.** Phosphorescence spectra of **2** at 77 K along with the phosphorescence spectra of the free ligand and anthracene.

**Table S5.** Photoluminescence properties of complexes **1** and **2** upon excitation 335 nm

| **Code** | **$CHCl_3$** | **$CH_3CN$** | **DMSO** | **$CH_3OH{:}C_2H_5OH$ (77 K)** |
|---|---|---|---|---|
| | **$\lambda_{em}$ [nm]** | **$\lambda_{em}$ [nm]** | **$\lambda_{em}$ [nm]** | **$\lambda_{em}$ [nm]** |
| **1** | 482 | 470, 604 | $^{I}$455,<br>$^{II}$712,783 | $^{I}$546, 576, 632<br>$^{II}$686, 760 |
| **2** | 479 | 578 | $^{I}$573,<br>$^{II}$708, 781 | $^{I}$520,<br>$^{II}$697,775 |

## Phosphorescence emissions – computational data

**Table S6**. The energies of theoretical phosphorescence emissions, calculated from the difference between the ground singlet and the triplet state $\Delta E_{T_1-S_0}$, along with the experimental values and the spin density surface plots for **1** and **2**. Grey and green colours show regions of positive and negative spin density values, respectively.

| Code | Experimental emission maximum | Calculated emission maximum | Spin density surface plots |
|---|---|---|---|
| **1** | 624<br>(1.99 eV) | 905<br>(1.37 eV) | 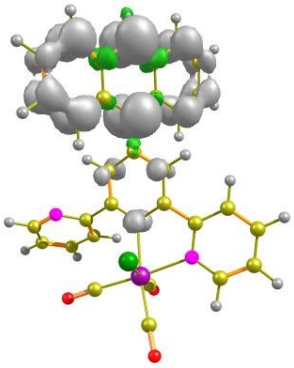 |
| **2** | 626<br>(1.98 eV) | 886<br>(1.40 eV) | 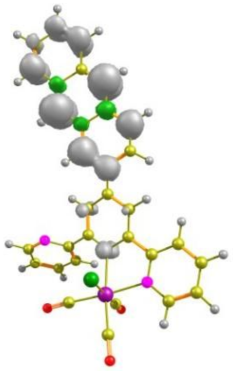 |

## Femtosecond transient absorption spectroscopy

Femtosecond transient absorption spectra were measured using Helios pump-probe transient absorption spectrometer (Ultrafast Systems). The solutions of **1** and **2** in $CHCl_3$/MeCN/DMSO with the absorbance of 0.2–0.7 at the excitation wavelengths were placed in 2 mm path length quartz cells with magnetic stirring, and excited with 355 nm or 405 nm pump pulses. A regenerative amplified femtosecond Ti:sapphire laser system (Astrella, Coherent) delivered pulses under 100 fs duration with 5 mJ pulse with repetition rate of 1 kHz and a central wavelength of 800 nm. The excitation pulses of 355 nm and 405 nm were generated from an optical parametric amplifier (Light Conversion, TOPAS prime). A white light continuum pulse was generated by focusing the residual of the fundamental light at $CaF_2$ crystal, which was used as a probe beam. The pump pulse was chopped by a mechanical chopper synchronised to one-half of the laser repetition rate (1 kHz), resulting in a pair of spectra with and without the pump, from which absorption changes induced by the pump pulse were estimated. Pump beam was depolarised to mimic dynamical changes of the orientation of the molecules. The delay time between pump and probe pulses was controlled by a moveable delay line in a time scale up to 7.5 ns. For the detection of the transient absorption signals, the white light continuum after passing through the sample was sent to CCD detector installed in the system.

Transient absorption data were prepared using the Surface Xplorer (Ultrafast Systems) software and then analysed with use of Optimus™ software. Correction of background, scattered light subtraction, solvent signal contribution subtraction as well as removing of spikes were performed routinely prior to the analysis. Moreover, coherent artefact analysis provided necessary information about IRF (IRF FWHM was estimated as about 180 fs ($CHCl_3$), 140 fs (MeCN) and 190 fs (DMSO) for 355 nm pump pulse and 160 fs in case of $CHCl_3$ and 405 nm pump pulse) and allowed to the corrections of the probe chirp. The global analysis was performed using linear unidirectional sequential model implemented in Optimus™ software. The analysis allows to the deconvolution of the transient spectra into species-associated spectra (SAS) and provides the decay-associated spectra (DAS) as a linear combination of the SAS of the compartments.

Prior to the femtosecond experiments both fluence dependence and photodamage tests were performed in order to determine appropriate experiment conditions for each sample. The pump-probe measurements were performed at the highest fluence within the linear regime. For each sample, the photodamage test under these conditions displayed no loss of absorption after fsTA experiment.

**Figure S20** Fluence dependence and photo-stability tests of **1** and **2** prior femtosecond transient absorption experiment.

<table>
<tr><td>Solvent</td><td>Fluence test</td><td>Photodamage test</td></tr>
<tr><td colspan="3">1 excited at 355 nm</td></tr>
<tr><td>$CHCl_3$</td><td>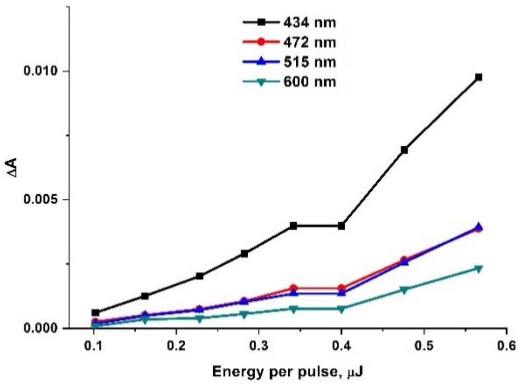
</td><td>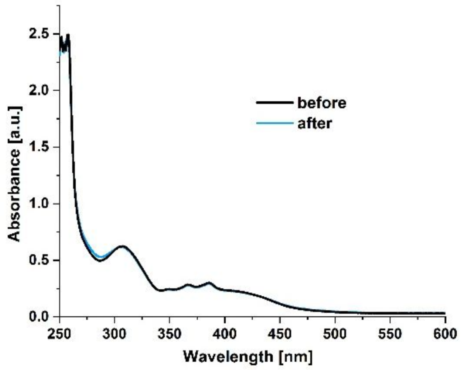
</td></tr>
<tr><td>MeCN</td><td>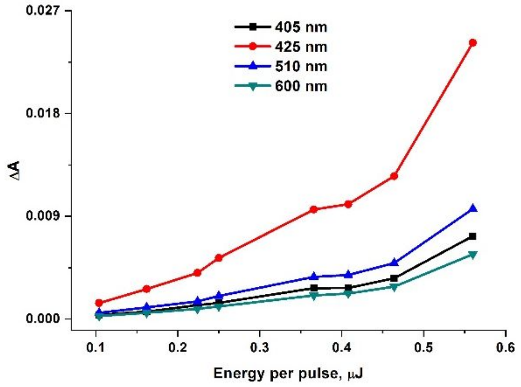
</td><td>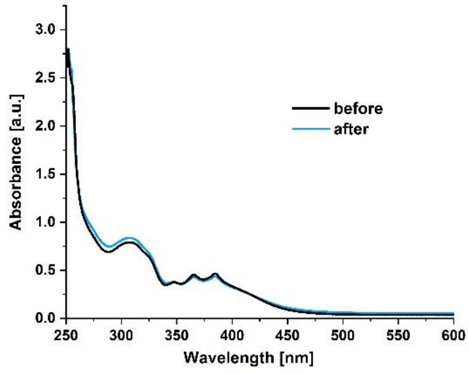
</td></tr>
<tr><td colspan="3">2 excited at 355 nm</td></tr>
<tr><td>$CHCl_3$</td><td>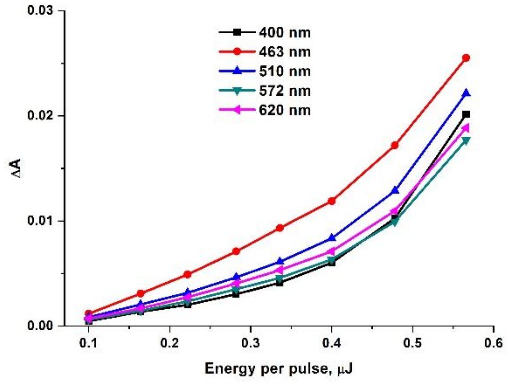
</td><td>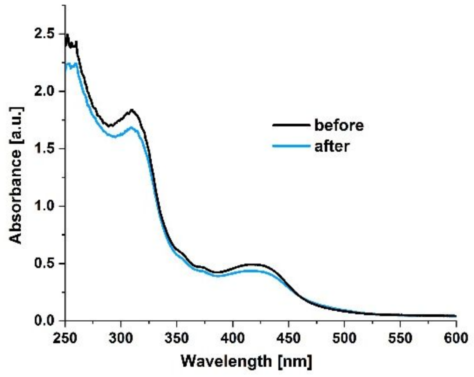
</td></tr>
</table>

<table>
<tr>
<td>MeCN</td>
<td>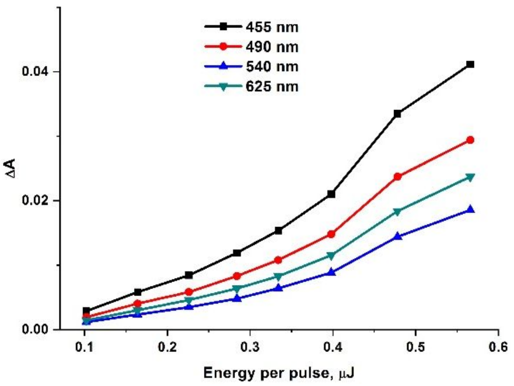
</td>
<td>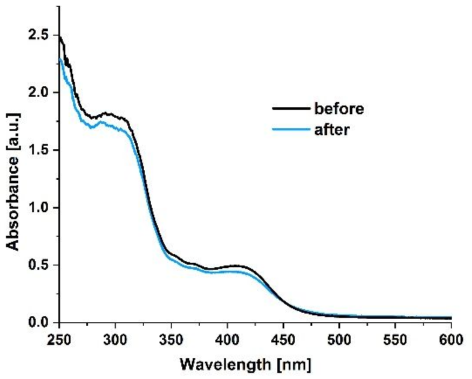
</td>
</tr>
</table>

<table>
<tr><th>Solvent</th><th>A</th><th>B</th><th>C</th></tr>
<tr><td colspan="4">1</td></tr>
<tr><td>$CHCl_3$</td><td>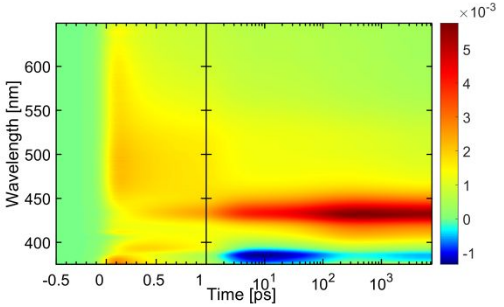
</td><td>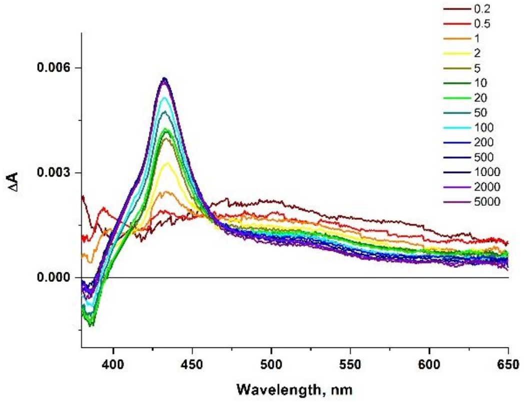
</td><td>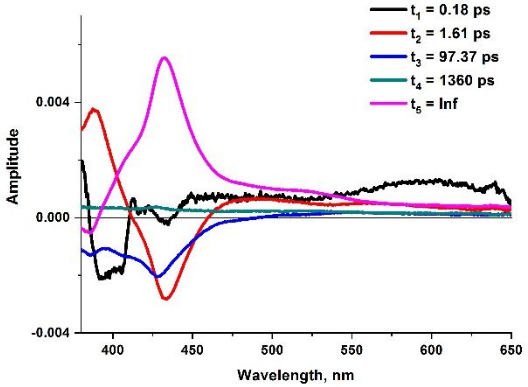
</td></tr>
<tr><td>MeCN</td><td>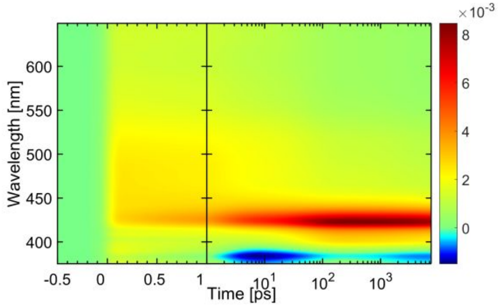
</td><td>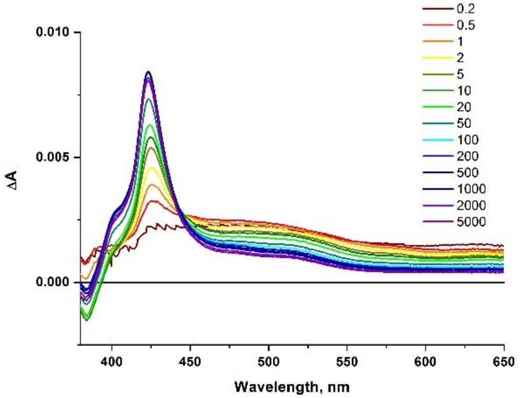
</td><td>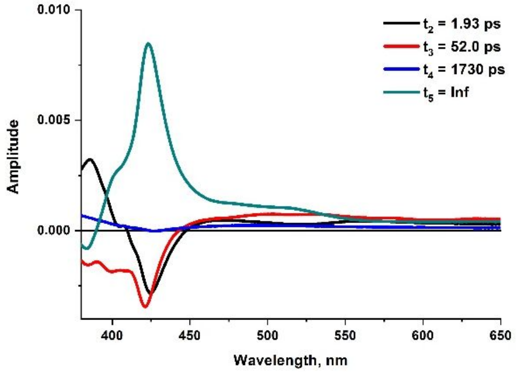
</td></tr>
</table>

<table>
<tr><td>DMSO</td><td>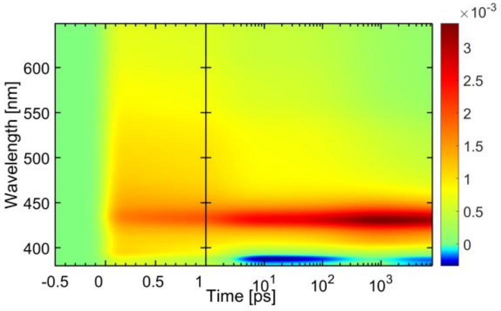
</td><td>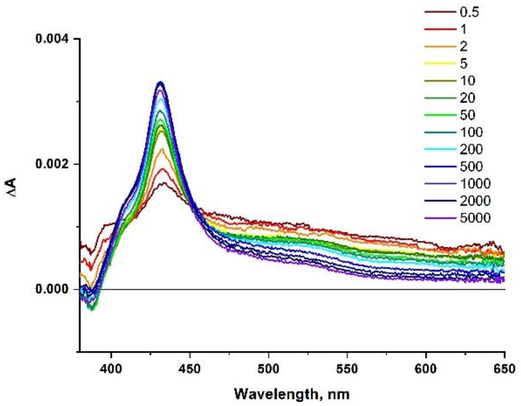
</td><td>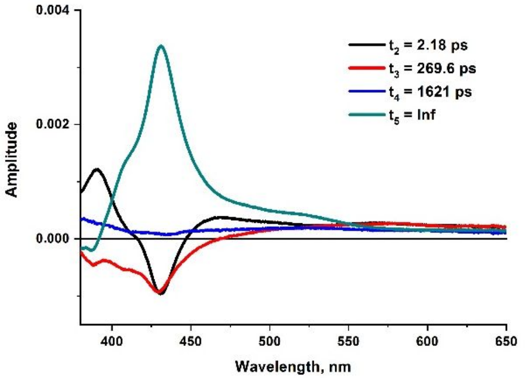
</td></tr>
<tr><td colspan="4">2</td></tr>
<tr><td>$CHCl_3$</td><td>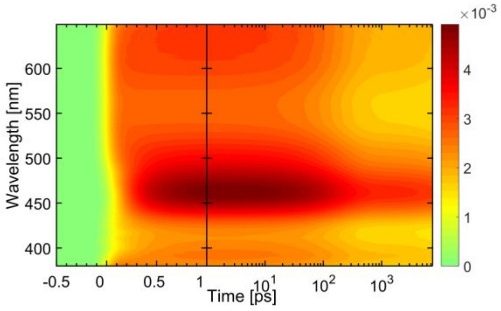
</td><td>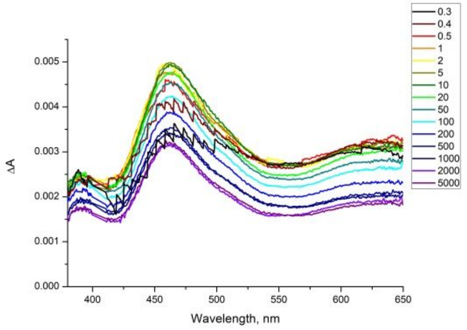
</td><td>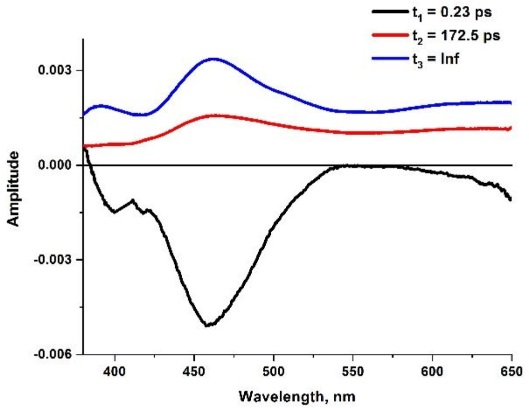
</td></tr>
</table>

MeCN

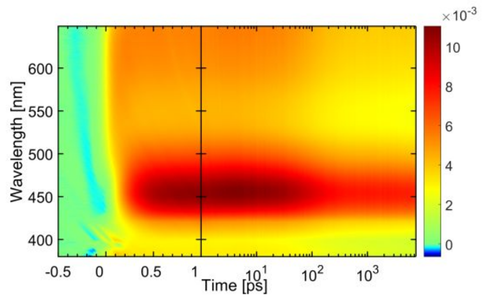

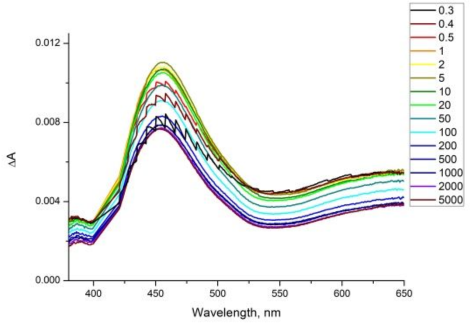

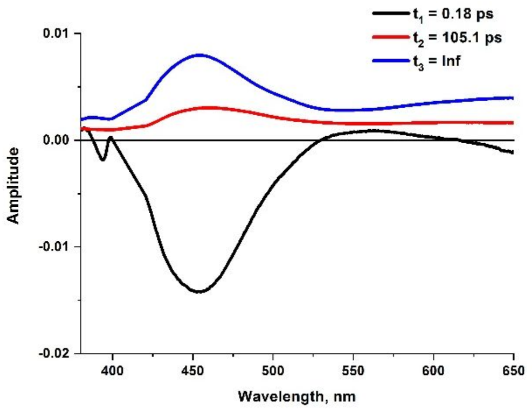


DMSO

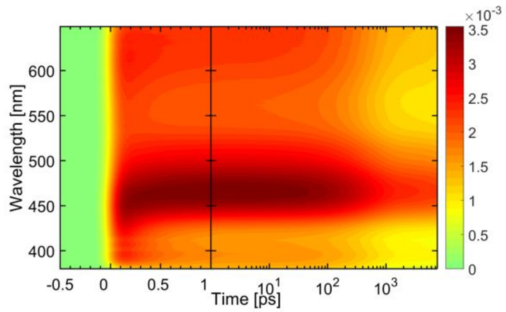

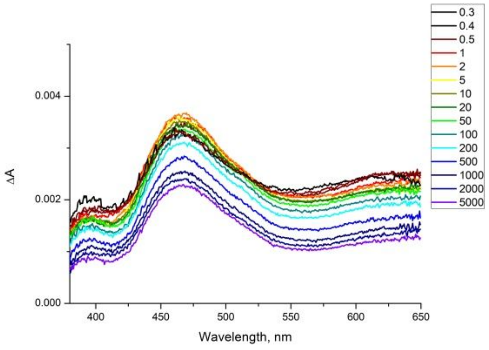

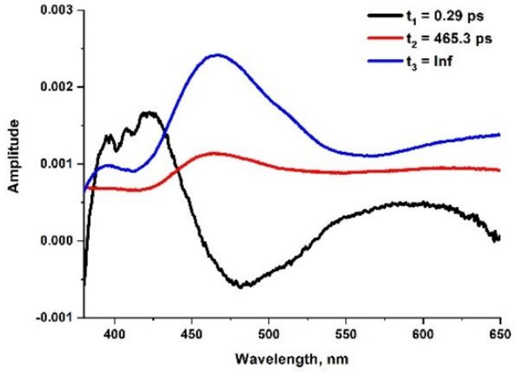


**Figure S21.** Summary of fsTA measurements for complexes **1** and **2** excited at 355 nm: fsTA 2D maps (panel A), TA spectra at selected time delays (panel B), and decay associated spectra, DAS (panel C).

<table>
<tr><td>Solvent</td><td>A</td><td>B</td><td>C</td></tr>
<tr><td colspan="4">1</td></tr>
<tr><td>$CHCl_3$</td><td>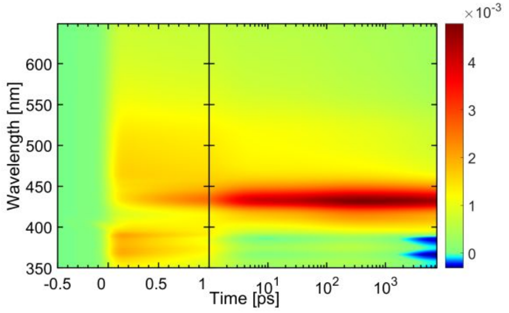
</td><td>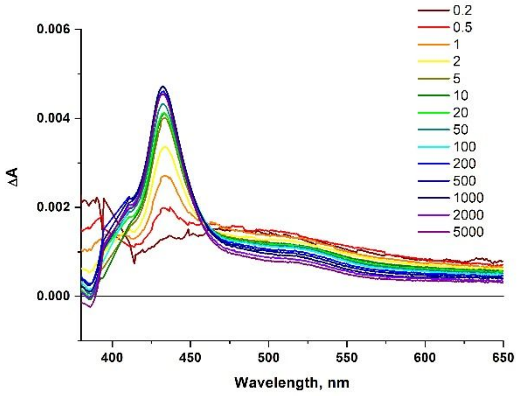
</td><td>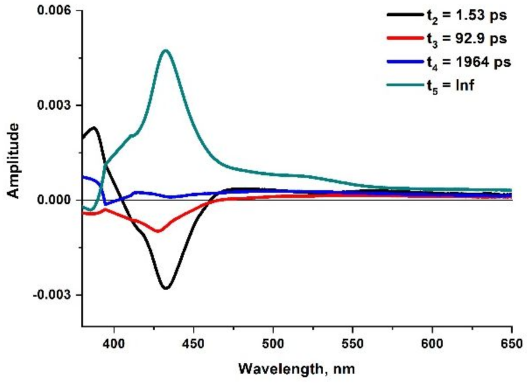
</td></tr>
<tr><td colspan="4">2</td></tr>
<tr><td>$CHCl_3$</td><td>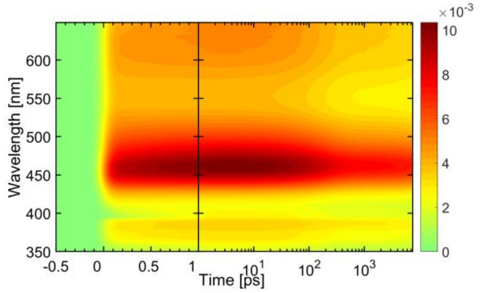
</td><td>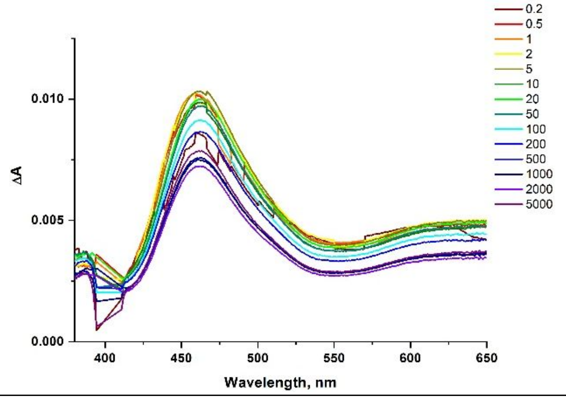
</td><td>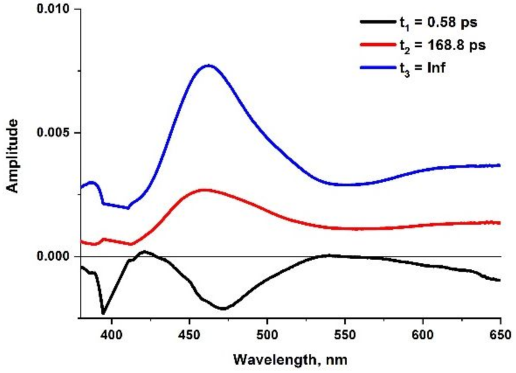
</td></tr>
</table>

**Figure S22**. Summary of fsTA measurements for complexes **1** and **2** excited at 405 nm: fsTA 2D maps (panel A), TA spectra at selected time delays (panel B), and decay associated spectra, DAS (panel C).

| | $CHCl_3$ | MeCN | DMSO |
|---|---|---|---|
| 1 | 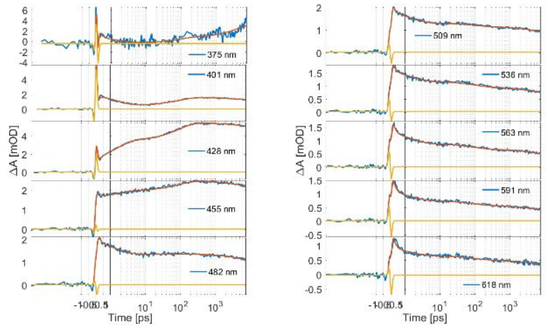 | 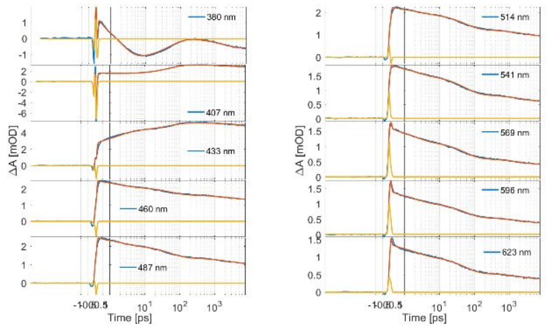 | 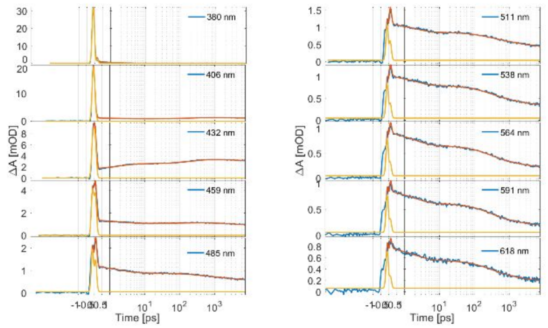 |
| 2 | 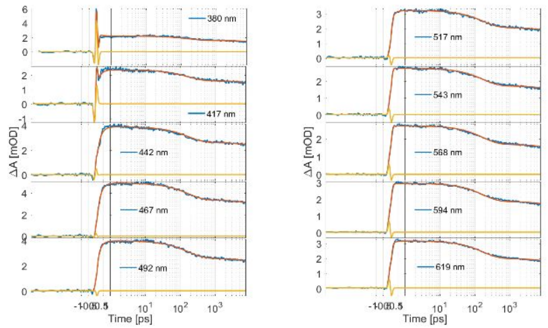 | 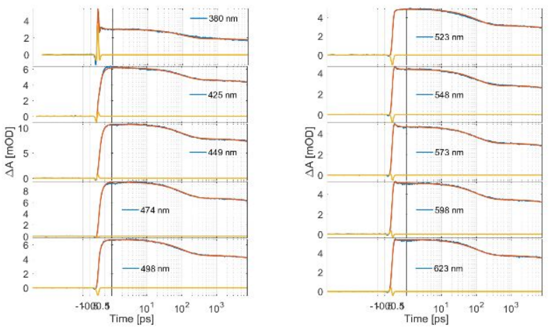 | 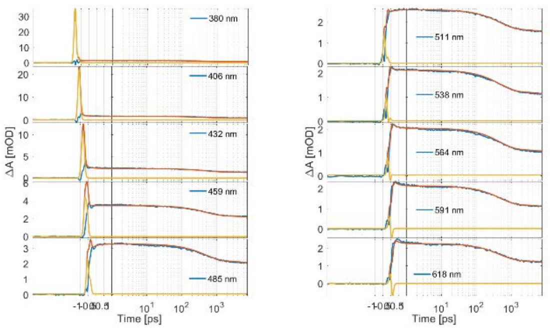 |

**Figure S23**. 2D maps presenting time traces along with the fitting curves from the global analysis for complexes **1** and **2** excited at 355 nm.

| 1 | 2 |
| --- | --- |
| 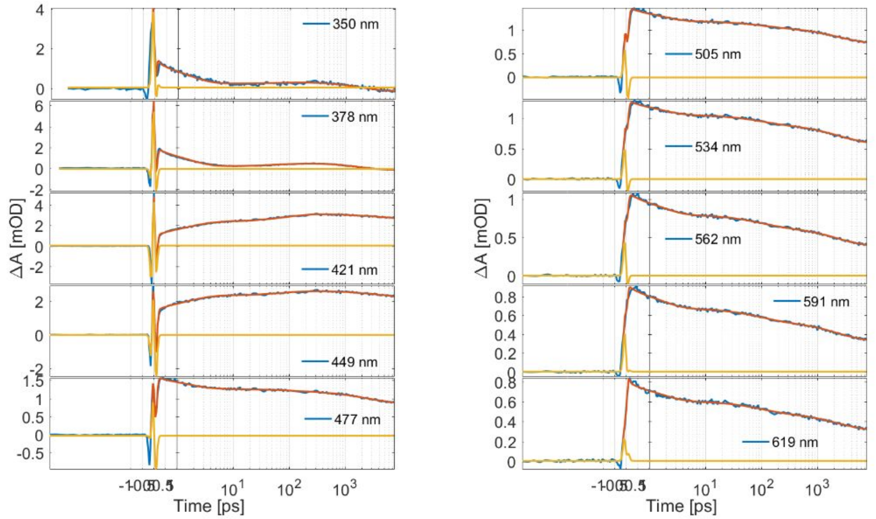 | 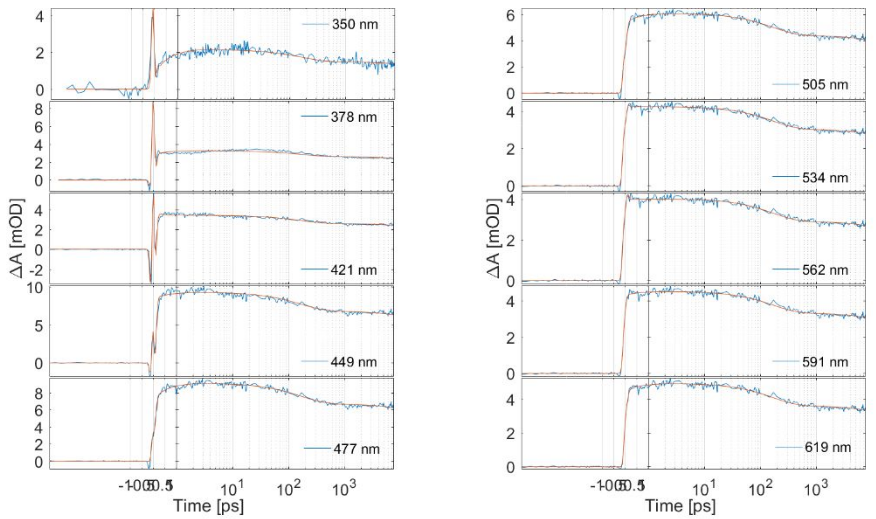 |



**Figure S24**. 2D maps presenting time traces along with the fitting curves from the global analysis for complexes **1** and **2** in $CHCl_3$ excited at 405 nm.

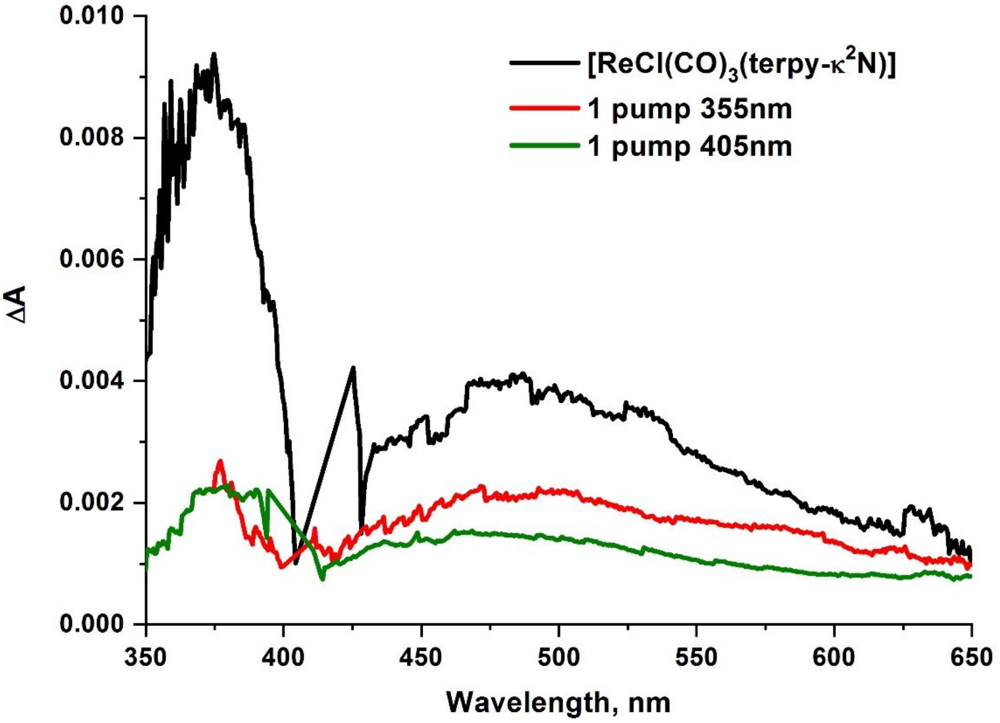


**Figure S25.** Comparison of fsTA spectra at early time delay (0.2 – 0.5 ps) of **1** and parent chromophore [ReCl(CO)$_3$(terpy-κ$^2$N)] [ref. 64 in manuscript]

## $^1O_2$ generation

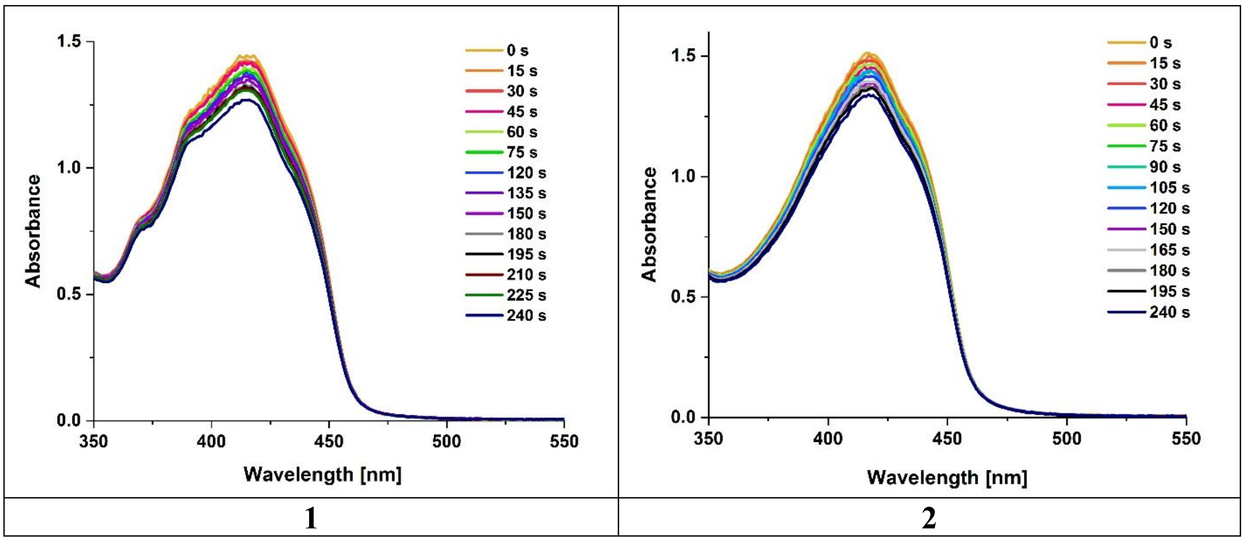


| | |
|---|---|
| **1** | **2** |

**Figure S26.** UV-vis absorption spectra of diphenylisobenzofuran (DPBF) in DMSO (c – 50 μM) treated with complexes **1** and **2** (c – 50 and 25 μM, respectively for **1** and **2**) upon exposure to visible light at 420 nm recorded over 240 s.

| | | |
|---|---|---|
| 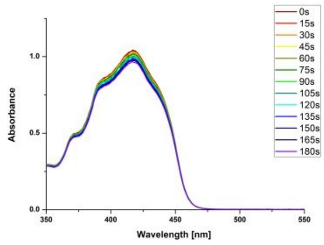 | 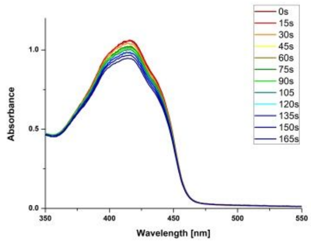 | 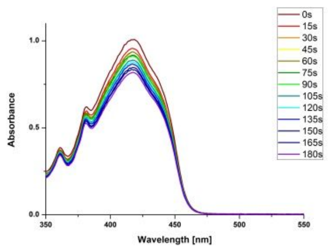 |
| **$L^1$ ($\Phi_\Delta$ - 0.21)** | **$L^2$ ($\Phi_\Delta$ - 0.23)** | **Anthracene ($\Phi_\Delta$ - 0.57)** |

**Figure S27.** UV-vis absorption spectra of diphenylisobenzofuran (DPBF) in DMSO (c – 50 μM) treated with free ligands and anthracene (c – 50 and 25 μM, respectively for **1** and **2**) upon exposure to visible light at 380 nm recorded over 240 s.

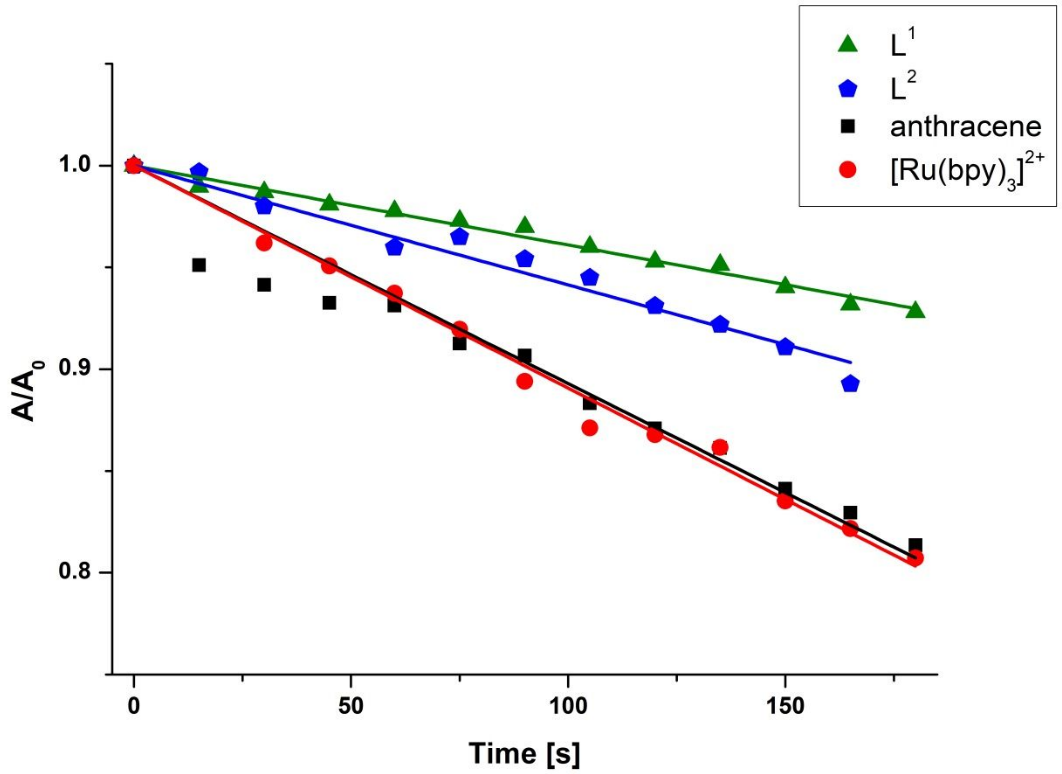


**Figure S28.** Relative changes in absorbance of DPBF at 417 nm ($A/A_0$) with time.
